# Data Analytics in Physical Activity Studies With Accelerometers: Scoping Review

**DOI:** 10.2196/59497

**Published:** 2024-09-11

**Authors:** Ya-Ting Liang, Charlotte Wang, Chuhsing Kate Hsiao

**Affiliations:** 1 Institute of Epidemiology and Preventive Medicine College of Public Health National Taiwan University Taipei Taiwan; 2 Institute of Health Data Analytics and Statistics College of Public Health National Taiwan University Taipei Taiwan; 3 Master of Public Health Program College of Public Health National Taiwan University Taipei Taiwan

**Keywords:** accelerometer, association, behavioral study, classification, digital biomarkers, digital health, physical activity, prediction, statistical method, wearable

## Abstract

**Background:**

Monitoring free-living physical activity (PA) through wearable devices enables the real-time assessment of activity features associated with health outcomes and provision of treatment recommendations and adjustments. The conclusions of studies on PA and health depend crucially on reliable statistical analyses of digital data. Data analytics, however, are challenging due to the various metrics adopted for measuring PA, different aims of studies, and complex temporal variations within variables. The application, interpretation, and appropriateness of these analytical tools have yet to be summarized.

**Objective:**

This research aimed to review studies that used analytical methods for analyzing PA monitored by accelerometers. Specifically, this review addressed three questions: (1) What metrics are used to describe an individual’s free-living daily PA? (2) What are the current analytical tools for analyzing PA data, particularly under the aims of classification, association with health outcomes, and prediction of health events? and (3) What challenges exist in the analyses, and what recommendations for future research are suggested regarding the use of statistical methods in various research tasks?

**Methods:**

This scoping review was conducted following an existing framework to map research studies by exploring the information about PA. Three databases, PubMed, IEEE Xplore, and the ACM Digital Library, were searched in February 2024 to identify related publications. Eligible articles were classification, association, or prediction studies involving human PA monitored through wearable accelerometers.

**Results:**

After screening 1312 articles, 428 (32.62%) eligible studies were identified and categorized into at least 1 of the following 3 thematic categories: classification (75/428, 17.5%), association (342/428, 79.9%), and prediction (32/428, 7.5%). Most articles (414/428, 96.7%) derived PA variables from 3D acceleration, rather than 1D acceleration. All eligible articles (428/428, 100%) considered PA metrics represented in the time domain, while a small fraction (16/428, 3.7%) also considered PA metrics in the frequency domain. The number of studies evaluating the influence of PA on health conditions has increased greatly. Among the studies in our review, regression-type models were the most prevalent (373/428, 87.1%). The machine learning approach for classification research is also gaining popularity (32/75, 43%). In addition to summary statistics of PA, several recent studies used tools to incorporate PA trajectories and account for temporal patterns, including longitudinal data analysis with repeated PA measurements and functional data analysis with PA as a continuum for time-varying association (68/428, 15.9%).

**Conclusions:**

Summary metrics can quickly provide descriptions of the strength, frequency, and duration of individuals’ overall PA. When the distribution and profile of PA need to be evaluated or detected, considering PA metrics as longitudinal or functional data can provide detailed information and improve the understanding of the role PA plays in health. Depending on the research goal, appropriate analytical tools can ensure the reliability of the scientific findings.

## Introduction

### Physical Activity

Physical activity (PA), defined as any body movement that results in energy expenditure [1], has been acknowledged as a crucial factor in health management and disease prevention [1-3]. The energy expenditure due to moderate and vigorous PAs, such as brisk walking and running, is greater than that due to sedentary behaviors such as rest. These moderate and vigorous PAs are beneficial for health [1]. Many studies, including clinical trials, have focused on their effectiveness in treating and preventing chronic diseases, as well as promoting general health, well-being, and longevity [4-8]. Information about PA can be collected through questionnaires and digital devices. While questionnaires may suffer from recall bias, digital devices offer a more objective evaluation. The quantified summary statistics of the values recorded by these devices are often called digital biomarkers [9]. In a scoping review of 31 systematic reviews of clinical evidence involving digital biomarkers, wearables were the most commonly applied measurement tool (22/31, 71%), and PA was the most prevalent intervention (16/31, 52%) [4].

### Wearable Devices

Wearable devices can continuously monitor an individual’s free-living PA patterns. With the growing emphasis on health consciousness, there has been a notable surge in studies focused on PA, driven by the popularity of activity-monitoring wearables [3]. Earlier studies commonly used uniaxial accelerometer devices that measure only vertical axis acceleration in *g*-units, corresponding to the acceleration due to gravity (9.81 m/s^2^) [10-13]. However, in recent decades, triaxial accelerometers operating in 3 orthogonal dimensions (*a_x_*, *a_y_*, and *a_z_*) have gained preference due to their ability to offer a broad coverage, thus providing a more comprehensive understanding of overall human activity [14]. Among triaxial accelerometers, wrist-worn wearables are predominant, as devices worn on other body parts such as waists, hips, or thighs may cause inconvenience during movement [3]. Table 1 lists only triaxial wearable accelerometers used in >1 article reviewed here, as well as the placement positions of each device. More than half of them are research-grade wearables (eg, ActiGraph [ActiGraph, LLC], GENEActiv [ActivInsights], Axivity [Axivity Ltd], and RT3 [Stayhealthy, Inc]), while some are commercial wearables (eg, Fitbit Watch [Fitbit Inc]). This review will not discuss the comparison of different devices. Readers interested in the practical use, reliability, and validity of accelerometers are referred to published reviews [15-20].

**Table 1:** Triaxial wearable accelerometers that appeared in >1 reviewed article among the 428 articles.

Brand and model	Frequency (Hz)	Software	Manufacturer	References, n (%)
ActiGraph GT3X, GT9X, wGT3X-BT, wGT3X+, GT3X+, and ActiTrainer	30-100	ActiLife research	ActiGraph, LLC	180 (42.1)
GENEActiv original	10-100	GENEActiv PC	ActivInsight	24 (5.6)
Axivity AX3	12.5-3200	OMGUI	Axivity Ltd	37 (8.6)
RT3	0.017-1	RT3 ASSIST	Stayhealthy, Inc	2 (0.5)
activPAL activPAL3 and activePAL4	20-80	activPAL software	PAL Technologies Ltd	7 (1.6)
Actical	32	Actical software	Mini-Mitter, Respironics	30 (7)
Actiwatch	0.25-32	Actiware	Philips Respironics	5 (1.2)
Xiaomi Mi Band 2	—^a^	Master for Mi Band app	Xiaomi Corp	2 (0.5)
TracmorD	0.3-20	DirectLife Activity Monitor software	Philips DirectLife	5 (1.2)
Hookie AM20	100	—	Traxmeet Ltd	6 (1.4)
UKK AM30 and RM42	100	—	UKK Institute	3 (0.7)
Kenz Lifecorder EX	32	Lifecorder transfer software	Suzuken Co, Ltd	3 (0.7)
Shimmer3	1-512	ShimmerCapture	Shimmer Research Ltd	2 (0.7)
Fitbit	1-256	Fitabase server	Fitbit Inc	6 (1.4)

^a^Not available.

Such individual-centered PA information is usually collected by wearables at a frequency of 30 to 100 observations per second (Hz). Most devices do not have built-in software to output the collected data. However, several external programs such as ActiLife and GGIR [21] are available for analysis. When each person is monitored for multiple days in a free-living environment, the data volume becomes enormous, leading to difficulties in analysis. Various digital variables that summarize daily activities have been proposed. Some are good for representing the total intensity, such as the summation or average of activity intensity (AI), and some are appropriate for the overall heterogeneity, such as variation and range, in either the time domain or the frequency domain [22]. These summary variables, however, do not catch the temporal patterns inherent in individual PAs. Other variables containing time information have been proposed and implemented in analyses. Part of this review will focus on these digital features.

Previous reviews have often emphasized the applications of wearables in health studies, focusing on the validity and reliability of these devices in evaluating PA and their use in clinical research [23-26]. While these reviews are comprehensive in terms of the practical use of PA metrics as digital data, they do not address the evolving trends in the analytical methods used to analyze such data. With the prevalence of wearables and the exponential increase in PA data volume, scientists now have the capacity to describe daily PA in a more sophisticated manner and examine whether PA trajectories vary across conditions or groups. Some advanced analytical tools have been proposed to address these tasks and will be discussed here.

In this review, we first describe the metrics currently used to summarize daily activities, particularly those transformed based on 3 acceleration axes. Common statistics derived from single-axis data, either in the time or frequency domain, are presented in Textbox 1 and will not be further discussed here. Second, we review the application of innovative PA metrics in statistical models aimed at different research goals, including the classification of activities, examination of the association of PA with health behavior or outcomes, and prediction of future events. Third, we discuss the limitations of current tools and propose directions for future analysis.

**Summary metrics and features calculated in time or frequency domain.**

**Domains, metrics, and features**

- Time, uniaxial: mean, variance, SD, percentile, minimum, maximum, range
- Time, between 2 uniaxials: correlation between 2 axials, covariance between 2 axials
- Frequency: dominant frequency, peak power, spectral energy, entropy, and spectrum centroid

## Methods

### Protocol and Registration

The methods used in this review align with the PRISMA-ScR (Preferred Reporting Items for Systematic Reviews and Meta-Analyses extension for Scoping Reviews) checklist shown in Multimedia Appendix 1 [27]. The Arksey and O’Malley [28] framework was used after making methodological adaptations for this review. Protocols used in this scoping review are detailed in the following sections.

### Eligibility Criteria

The eligibility criteria for article reviews have been established (Multimedia Appendix 1). The inclusion criteria were as follows: (1) the PA data reported on were obtained from human participants (adults or children, with disease or healthy) through a wearable accelerometer and (2) the purpose of the study containing the analysis of accelerometer data was to capture human activity patterns and influence on health.

The exclusion criteria included (1) articles not written in English; (2) articles incorporating other physiological activities detected by electrical signals (eg, electrocardiography); (3) articles conducting only statistical testing or containing no statistical model; (4) articles not focusing on human activity patterns; (5) articles not of original research, including systematic reviews, scoping reviews, books, and book chapters; (6) articles focusing on the reliability of wearable devices; and (7) articles addressing the upstream preprocessing of accelerometer data or measurement design (eg, the choice of epoch length of acceleration data or the placement of devices).

### Information Sources

To collect the potentially relevant articles, the search was conducted in each of the following 3 publication databases: PubMed, IEEE Xplore, and ACM Digital Library. The final search was performed on February 29, 2024. As the main objective of this scoping review was to provide an overview of analytical methods used for the analysis of PA data in recent years, the search was constrained to articles published between 2000 and 2024. The full search strategies used in the 3 databases can be found in Multimedia Appendix 2.

## Results

### Selection of Evidence Sources and Data Extraction

**Figure 1:**
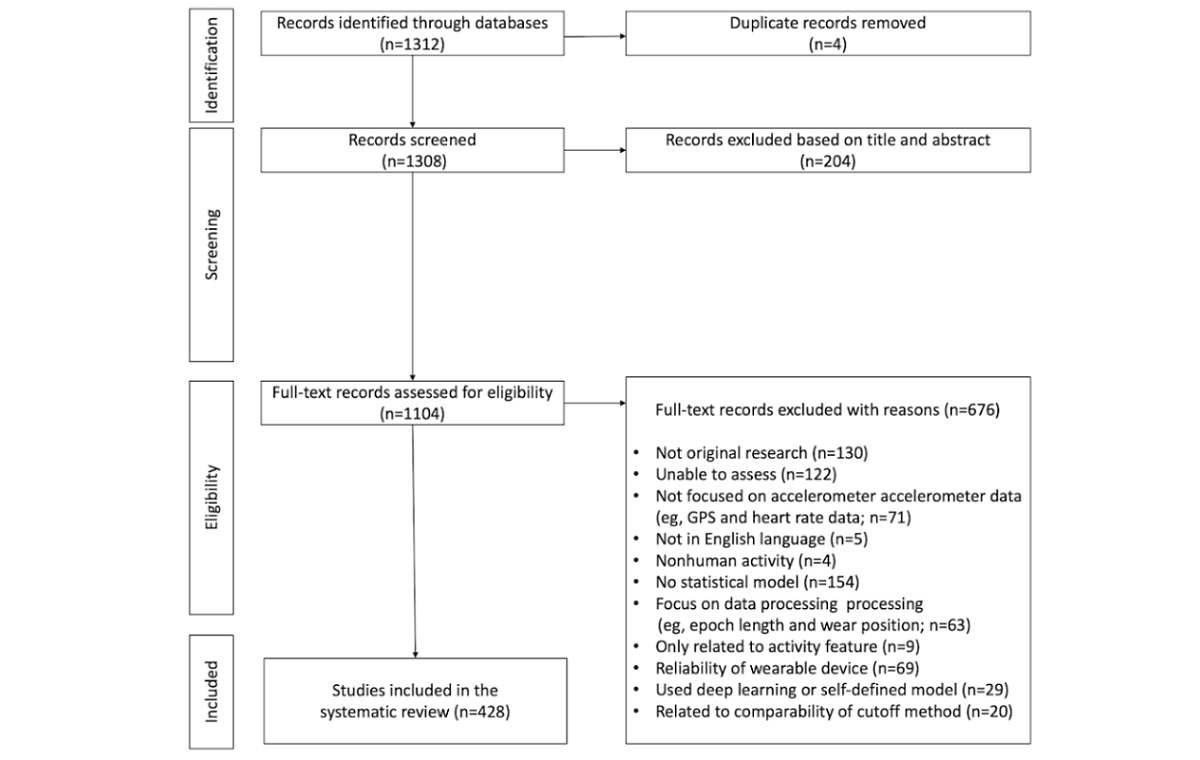
Flowchart of the selection strategy.

After removing duplicate articles from the initial 1312 articles obtained by searching the 3 databases, screening for study goals based on title and abstract, and assessing eligibility (Figure 1), a final set of 428 articles was included in this review (Multimedia Appendix 3 [5,13,29-454]). Of them, 407 (95.1%) articles were from PubMed, 20 (4.7%) articles were from IEEE Xplore, and 1 (0.2%) article was from the ACM Digital Library. A total of 2 independent reviewers examined the content of each paper to determine which of the 3 categories of studies (activity classification, association, and prediction) it fit into and recorded the analytical tools used. Any inconsistencies in judgment were discussed and resolved through consensus. Following this, other study variables such as data domain, PA metrics, and summary statistics were examined.

The extracted data included brands and other details of the accelerometers used, research focus, context, analytic methods, and outcome variables. The extraction process was conducted using a Microsoft Excel (Microsoft Corp) sheet designed by the reviewer YTL. This sheet consolidated the context of each article, capturing information on the brand of the device, PA variables, research issues addressed, activity features examined, statistical methodologies applied, and study outcomes.

### Measurements From Accelerometers

Most wearable accelerometers measure acceleration in 3 dimensions (*a_x_, a_y_, a_z_*), with some also equipped with gyroscopes to assess body angular rates in degrees per second across the 3 axes. The reliability and accuracy of various brands, including ActiGraph, GENEActive, and Axtivity, have been compared and reviewed [15-20]. Because acceleration measurements are more common than gyroscope measurements in most devices, they have become the primary variable for evaluating body movement.

These accelerometer devices provide either raw accelerations in 3 dimensions or a combined metric as activity count (AC) per epoch produced by the manufacturer’s proprietary software (Table 1) or by other free programs such as GGIR [21]. Raw accelerations have been favored over AC for 3 reasons. First, it is uncertain whether the definition of AC across brands is the same. Second, the development of free software such as GGIR [21] has relieved the computational difficulty and burden on researchers of analyzing acceleration measurements. Third, providing raw acceleration data makes them available for reuse in other types of studies.

**Figure 2:**
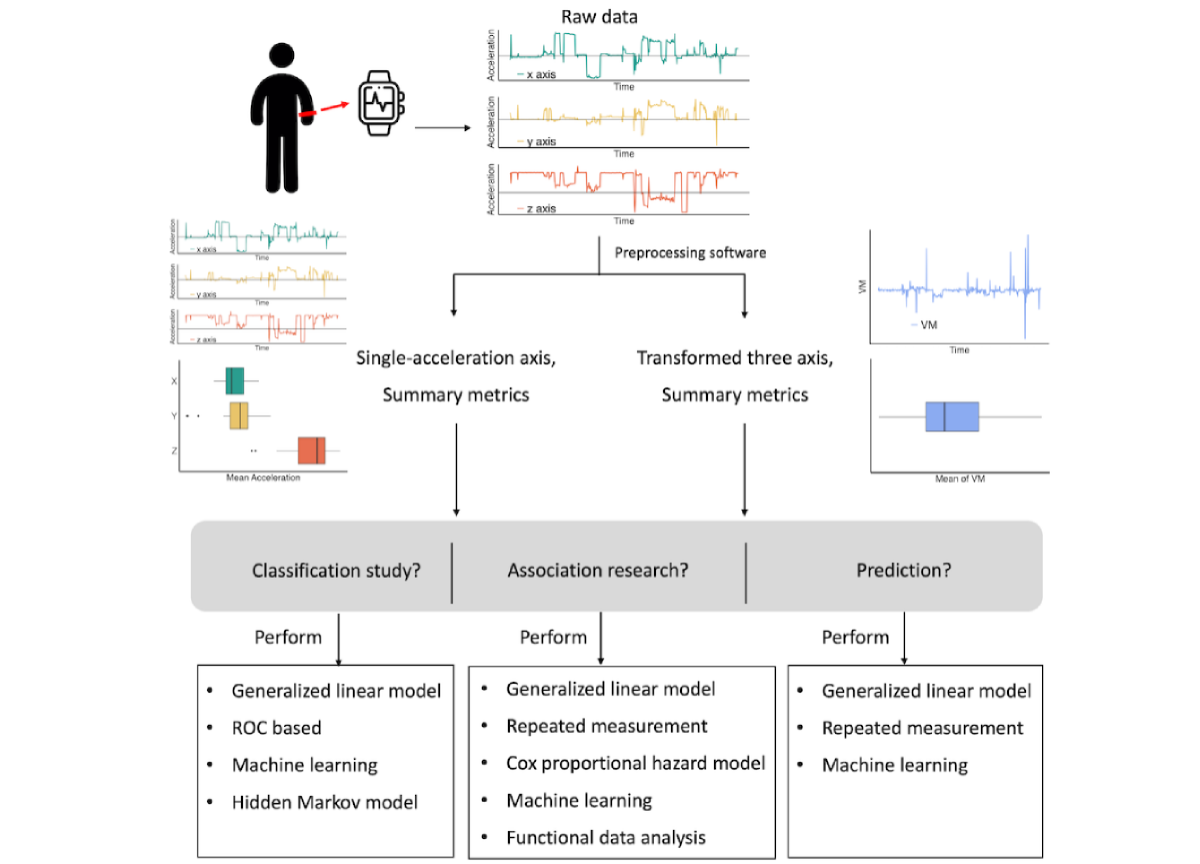
Outline of procedures from the collection of raw data to the application of analytical methods. PA: physical activity; ROC: receiver operating characteristic; VM: vector magnitude.

**Figure 3:**
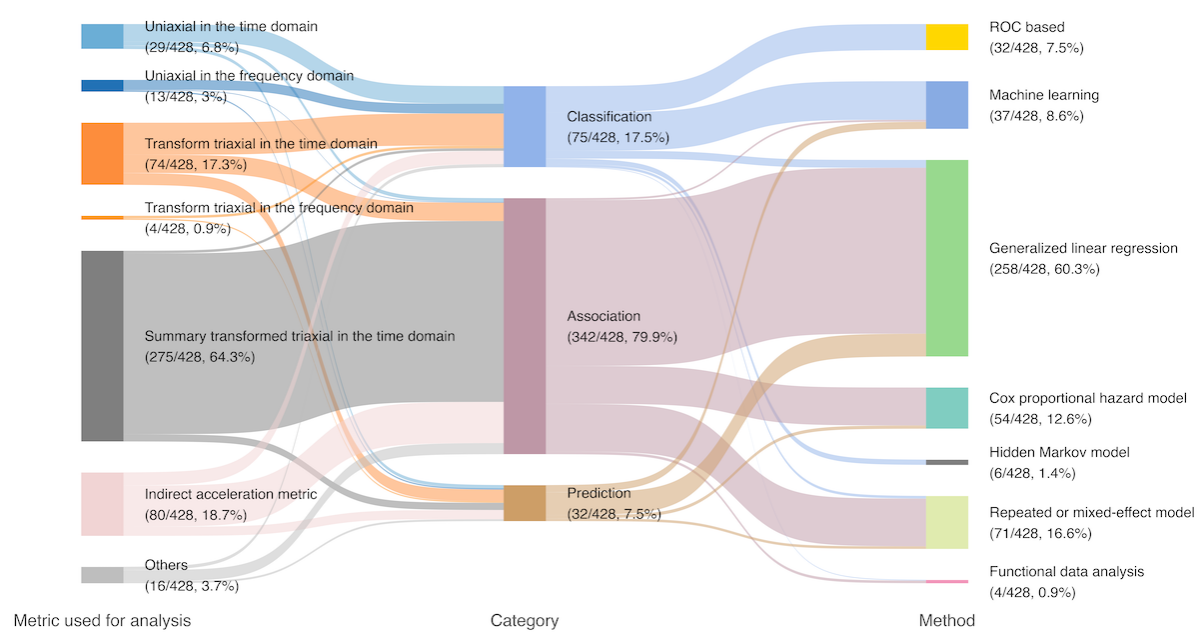
The left column lists the number of articles that used the corresponding physical activity (PA) metrics in analysis, the middle lists the number of articles focusing on each topic, and the right column lists the analytical tools adopted in studies. Note that an article may be counted more than once if it qualified >1 criterion. ROC: receiver operating characteristic.

Accelerations in 3 dimensions can be analyzed either separately or after being transformed into a single variable, as shown in Figure 2. Both uniaxial acceleration and transformed triaxial metric data can be used in analysis to classify activity types, explore association, and predict health outcomes. Figure 2 outlines the procedures from the collection of raw data to analysis of uniaxial or triaxial acceleration data. These analyses can be categorized further, as displayed in Figure 3. In both the time and frequency domains, 29 (6.8%; 29 uniaxial in time + 13 uniaxial in frequency – 13 duplicates) of 428 studies examined the uniaxial acceleration separately for its simplicity and straightforwardness. A total of 340 (79.4%; 74 transform triaxial in time + 4 transform triaxial in frequency + 275 summary of transform triaxial in time – 13 duplicates) of 428 studies used a triaxial acceleration metric combining the acceleration data from 3 dimensions in the time domain or frequency domain, or used a summary-type metric based on these transformations. These include the length of the acceleration vector (*a_x_, a_y_, a_z_*), functions of the vector, amplitudes of Fourier-transformed acceleration waves, and summary statistics of these metrics. Analyses based on transformed variables have become prevalent in recent years because they combine information from all 3 acceleration axes. We refer to the variables transformed from the 3 raw accelerations as *transformed metrics*, which can be represented in the time domain or frequency domain. Some articles (80/428, 18.7%) used indirect acceleration metrics such as step count, AC, and walking time.

### Metrics in Analysis: Transformed Metrics in the Time Domain

Several transformed metrics have been developed as functions of the 3 accelerations (*a_x_, a_y_, a_z_*), including the Euclidean norm minus one (ENMO), signal magnitude area, angle-z, and PA index (Table 2). These metrics either focus on the vector magnitude (VM) of the acceleration (magnitude type) or the variability in the acceleration. For instance, the first developed metric was the straightforward VM based on the 3 uniaxial accelerations at time *t*:

VM = [ *a_x_*(*t*)^2^ + *a_y_*(*t*)^2^ + *a_z_*(*t*)^2^ ]^1/2^  **(1)**

**Table 2:** Transformed metrics in time domain and their summary quantities calculated from 3 accelerations (*a_x_*, *a_y_*, *a_z_*).

Metric, feature, and summary	Definition	Reference
ENMO^a^	max{[ *a_x_*(*t*)^2^ + *a_y_*(*t*)^2^ + *a_z_*(*t*)^2^]^1/2^ – 1, 0}	[455]
Angle-z	tan^-1^{ *a_z_*(*t*) / [ *a_x_*(*t*)^2^ + *a_y_*(*t*)^2^]^1/2^} × 180 / π	[8]
PAI or AI^b^	AI(*t,H*) = [max{ (var(*a_x_*(*t,H*)) + var(*a_y_*(*t,H*)) + var(*a_z_*(*t,H*)) – sum.var) / 3, 0}]^1/2^ sum.var = var(*a_x_*) + var(*a_y_*) + var(*a_z_*)	[456]
SMA^c^	sum of integration of |*a_x_*(*t*)|, |*a_y_*(*t*)| and |*a_z_*(*t*)| from *t* = *T* to *T* + 1 seconds	[29]
VM or SVM^d^	[ *a_x_*(*t*)^2^ + *a_y_*(*t*)^2^ + *a_z_*(*t*)^2^ ]^1/2^	[30,31]
MVPA^e^	number of times when the transformed metrics > threshold	[34-36]
L5^f^	The means of the 5 least active hours during the day	[457]
M10^g^	The means of the 10 most active hours during the day	[457]
IV^h^	{mean of (*x_h_* – mean(*x_h_*))^2^ from *h* = 1 to *p*} / {mean of (*x_i_* – mean(*x_i_*))^2^ from *i* = 1 to *N*} *N*: the total number of data points *p*: the number of data points per day *x_h_*: values of each hour from the mean 24-hour profile *x_i_*: each given hour of raw data	[457]
IS^i^	{mean of (*x_i_* – *x_i-1_*)^2^ from *i* = 1 to *N*} / {mean of (*x_i_* – mean(*x_i_*))^2^ from *i* = 1 to *N*} *N*: the total number of data points *x_i_*: the value of a given hour	[457]
Acrophase	The time at which the peak of a rhythm occurs	[458]

^a^ENMO: Euclidean norm minus one.

^b^PAI or AI: physical activity index or activity intensity.

^c^SMA: signal magnitude area.

^d^SVM: vector magnitude or signal vector magnitude.

^e^MVPA: moderate-to-vigorous physical activity.

^f^L5: least active 5-hour period.

^g^M10: most active 10-hour period.

^h^IV: intradaily variability.

^i^IS: interdaily stability.

The second developed metric, ENMO, is intuitive because it combines the strength of acceleration in gravity in the 3 axes, where the effect of gravity is removed from the formula by subtracting 1 [455]:

ENMO = max{[ *a_x_*(*t*)^2^ + *a_y_*(*t*)^2^ + *a_z_*(*t*)^2^ ]^1/2^ – 1, 0} **(2)**

Its calculation can be performed with the R (R Foundation) package *GGIR* [21]. ENMO does not depend on the sampling frequency and is noted for its higher sensitivity to vertical and horizontal accelerations [455]. It has been widely used in studies for classifying specific movements and sleep stages [459,460]. Studies have showed that ENMO performs better at indicating moderate to heavy activities but is less robust at indicating sedentary and light activities compared to other measurements [456].

Other metrics similar to ENMO in calculating the “average” or “degree” of accelerations are signal magnitude area [29] and signal VM [30,31]. These metrics are all representations of the average type. These metrics can be further transformed to imply certain levels of activity. For instance, the moderate-to-vigorous PA (MVPA) measure contains cutoff values for ENMO to determine whether the activity is light, moderate, or vigorous [32,33]. In studies where the classification of an individual’s activity is of interest, the cutoff values need to be determined because the values may be person specific.

Another magnitude-type transformed metric is the heuristic algorithm looking at the distribution of change in z-angle proposed by van Hees et al [8]:

Angle-z = tan^-1^{ *a_z_*(*t*) / [ *a_x_*(*t*)^2^ + *a_y_*(*t*)^2^]^1/2^} × 180 / π **(3)**

As the name suggests, this transformed metric emphasizes the movement in the direction vertical to the x-y plane, and the use of *a_z_* increases the sensitivity for the detection of activities along this direction [460]. The heuristic algorithm looking at the distribution of change in z-angle was originally used to detect the sleep period time window. Its reliability has been demonstrated through comparisons with polysomnography [8], and it has been commonly used to investigate sleep conditions. For example, it was used by Jones et al [461,462] to differentiate sleep status, and it was combined with genomic data to explore significant sleep factors.

In contrast, PA index (PAI), also known as AI, examines the variability of accelerations, rather than their magnitude. The PAI metric combines the individual-specific variation in each dimension in a time interval of length *H* starting at time *t* as a summary metric [456]:

PAI(*t*,*H*) = [max{ (var(*a_x_*(*t*,*H*)) + var(*a_y_*(*t*,*H*)) + var(*a_z_*(*t*,*H*)) − sum.var) / 3, 0}]^1/2^  **(4)**

Here, the normalization quantity sum.var = var(*a_x_*) + var(*a_y_*) + var(*a_z_*), summation of 3 variances, is the systematic noise variance that can be computed from raw accelerations when the device is not moving. This PAI can be further modified by dividing the normalization quantity, becoming the relative AI, as opposed to the original version in the absolute scale. This AI is advantageous because of its additive and rotational invariance properties, indicating that PAI is a constant when an individual performs the same activity during the period. PAI can be applied in, among other areas, the surgical recovery process of women who have undergone mastectomies and evaluation of physical intensity during powered wheelchair mobility in people with severe dyskinetic cerebral palsy [463,464].

### Metrics in Analysis: Metrics Summarizing PA in the Time Domain

Metrics such as those discussed earlier are all computed in the time domain. In other words, they preserve time information and can be used further to decipher longitudinal patterns. These repeated time-series measurements can be easily included in the generalized linear mixed-effects models (GLMMs) for additional analysis, or they can be summarized or categorized to simplify the time effect. One such summary statistic is the threshold that differentiates light activity from MVPA, with the time spent in MVPA also considered as a summary PA feature.

Examples of MVPA include dancing, gardening, walking briskly, jogging, and running. To be categorized in this group, an activity usually needs to reach a certain intensity evaluated by metabolic equivalent of task (MET), such as the maximum or peak oxygen uptake or heart rate [34-36]. For example, one unit of MET is the amount of oxygen consumed or energy used per kg per minute while sitting at rest. Activities corresponding to MET values falling within a specified interval are categorized as MVPAs. The boundaries or thresholds of this interval need to be determined first. The concept of MVPA is easy to understand and often referenced when exercise is prescribed to people for health benefits; the definition of its thresholds, however, can be individual specific because the thresholds are related to individualized homeostasis disturbance [465].

Other summary metrics compare PA data within the same person to indicate a person’s rest-activity rhythm, including nonparametric circadian rhythm measures such as interdaily stability (IS), intradaily variability (IV), least active 5-hour period, most active 10-hour period, and the acrophase in cosinor analysis [458]. Both IS and IV are useful in describing rest and activity rhythms: IS provides information on how stable the rest-activity pattern is between different days, and IV quantifies the stability of the fragmentation in the activity pattern between time intervals [457].

These summary metrics effectively capture and quantify the characteristics of AI and circadian rhythms, enhancing the understanding of their relation to health and behavior [37,462]. However, defining thresholds for PA intensity can be both population and person specific [465]. In addition, these summary statistics may suffer from information loss because daily PA is a continuum [38]. They focus on activities in fragments of the day rather than the overall PA profile throughout the day.

### Metrics in Analysis: Metrics Summarizing Temporal Patterns in the Frequency Domain

One way to explore daily continuous temporal trends and trajectories is via the Fourier transformation, allowing metrics to be decomposed into periodic curves expressed by both frequency and amplitude. These longitudinal waves in the frequency domain are threshold free. For instance, ENMO, angle-z, and AI can be represented in the frequency domain to examine their patterns over time. Among the 428 reviewed articles, this frequency representation was considered in 4 (0.9%) articles for triaxial accelerations, while 13 (3%) adopted frequency domain representation in uniaxial acceleration.

We use *x*(*t*) to denote the transformed metric in the time domain, where *t* indexes the time. For example, *x*(0), *x*(1),..., *x*(*t*),..., and *x*(*T*_1_) represent the ENMO value observed throughout the day. Then, these *x*(*t*)s can be converted to *X^F^*(0), *X^F^*(1), *X^F^*(2),..., and *X^F^*(*T*_1_), where each one is a combination of sinusoid functions. In other words, these *X^F^*(*k*)s are all periodic curves with frequency *k* and amplitude ||*X^F^*(*k*)||. These new curves are in the same unit as the original *x*(*t*), and in the case of ENMO, the unit is *g*:

*X^F^*(*k*) = sum{*x*(*t*) × exp(−2* πikt* / *T*)} over *t* from 0 to *T*−1 **(5)**

For example, *X^F^*(2) implies that this individual performed an activity of amplitude ||*X^F^*(2)|| with frequency 2. Usually, the waves with large strength indicate dominant activities in a day, while the waves with low frequencies can imply regular rhythms such as commuting to and from work or napping habits. The computation of this transformation can be made easy with the fast Fourier transformation (FFT) in R with the package *stats*.

The advantages of the FFT are 3-fold. First, it simplifies the interpretation of periodic activities using frequency and strength. Second, as the PA curve is dominated by a few low-frequency waves, each curve can be represented with a small number of waves, significantly reducing data dimension. Third, these dominant waves can be considered as digital features of an individual’s PA and incorporated into further analyses, such as classification [39,40].

The FFT-based variables are useful for periodic activities, especially when activity metrics from multiple days are collected to establish an individual’s daily cycles. However, the FFT approach can be inappropriate if the original PA lacks periodic patterns. An alternative way would be to relax this constraint when summarizing the PA trajectory.

### Metrics in Analysis: Metrics of Temporal Patterns in the Time Domain

An alternative threshold free approach to capture the temporal dynamics inherent in accelerometer-based measures involves considering these measures as curves within infinite-dimensional spaces, treating these collected measures as functional data. Functional data analysis (FDA) has emerged as a powerful tool for analyzing PA data, enabling the continuous variation of PA patterns throughout the monitoring period, without the need to assume cycles. Furthermore, considering PA as a function of time facilitates the assessment of time-varying effects of covariates such as BMI, expanded disability status scale, and lung function [38,41,42,466-469]. Among the 428 screened articles, 4 (0.9%) used such features in PA analysis.

In FDA, the observed transformed metrics in a day from the same individual, *x*(0), *x*(1),..., *x*(*t*),..., and *x*(*T*1) are considered the discretized observations from an unknown continuous curve, *Y*(*t*). The patterns inherent among the multiple curves, *Y*(*t*)s, from multiple individuals can be explored via functional principal component analysis (FPCA), an analogy to principal component analysis for multivariate data [470,471]. These extracted principal component functions are called eigenfunctions and can indicate the decomposed daily temporal patterns. Each of the original curves is now associated with these eigenfunctions through a score. For instance, the *i*-th score of a daily curve now corresponds to its representation along with the *i*-th function. A large positive score indicates greater similarity in pattern and magnitude with this function. In other words, these scores become indications of the temporal trajectories.

In addition, similar to FFT, the top few principal component functions in FPCA can explain most of the variability in the data. This greatly reduces the original data size to a few dominant functions, which can describe the major activity patterns in the population. In other words, the original data set can be approximated with only a few functions and scores with this FPCA approach. FPCA has previously been used in modeling yearly temperature curves and constructing growth curves of children; however, few studies have considered the application of FPCA and FDA in PA studies [43,469]. Another group of articles incorporated within-person and between-person heterogeneity into their analysis using 2-level FPCA or multilevel FPCA [470,472]. One level of scores represents the person-to-person variation, while the second level represents the day-to-day variation [470,472]. FDA involves smoothing techniques, FPCA, functional linear regression, and clustering or classification methods for analyzing data observed over continuous domains. It has been adopted mostly in classification research, which we will discuss in the next sections.

### Activity Classification Studies With Threshold-Based Methods

**Figure 4:**
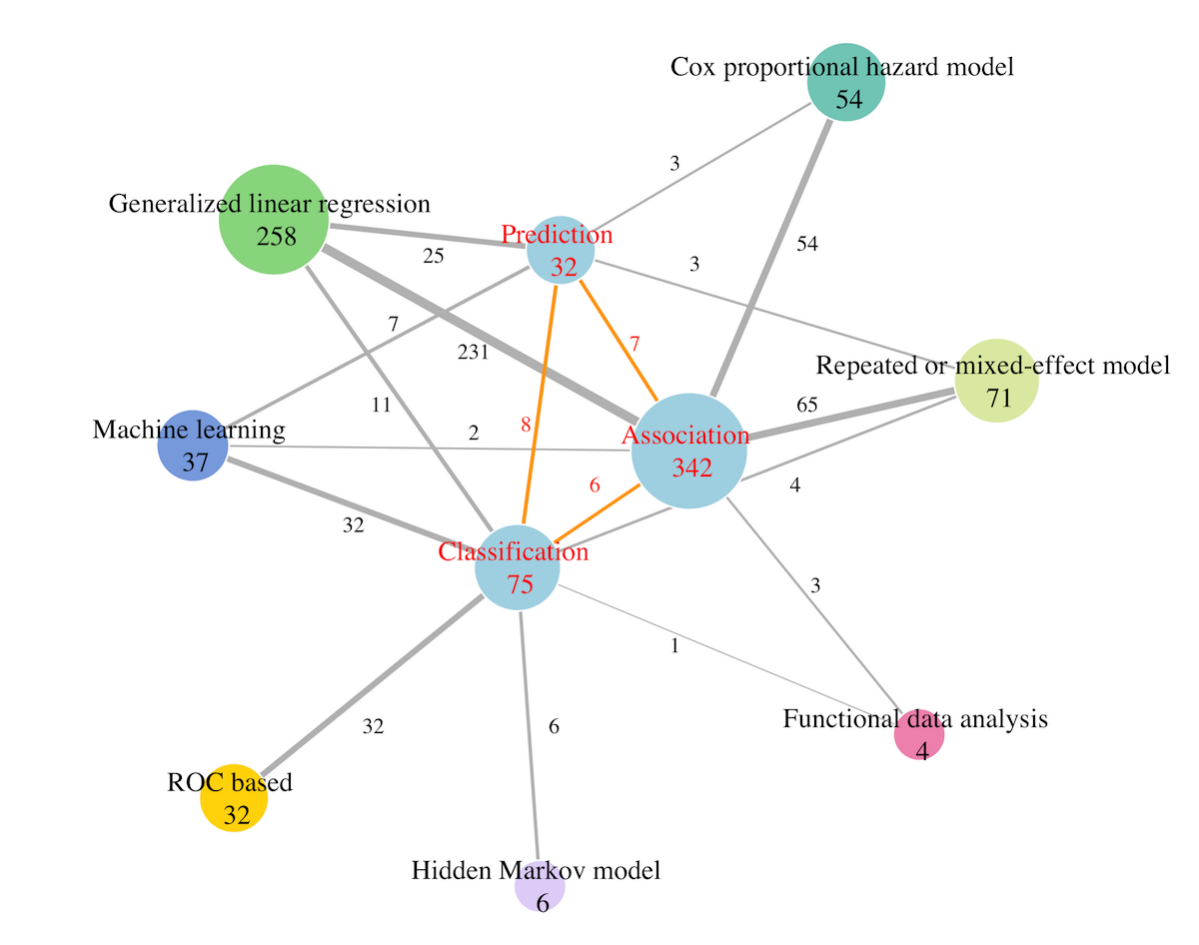
Network association plot of the articles. The number inside the node indicates the number of articles, and the number by the edge indicates the number of articles containing terms in both connecting nodes. ROC: receiver operating characteristic.

Most early studies involving PA measured by accelerometers were classification studies that classified activity types or intensities, such as sleep-wake or moderate or vigorous activities. Of the 428 reviewed studies, 75 (17.5%) fall into this category (middle panel in Figure 3 and Multimedia Appendix 3). The main 2 topics in these classification studies are the classification of specific activities and sleep-wake stages. Specific activities for classification include daily activities (sitting, walking, and lying) [13,44,45], housework (washing dishes and dust mopping) [46,456], fall detection [473,474], and rehabilitation activities [475,476], while sleep stages for classification include wake, light sleep, and deep sleep stages [8,460,477]. Each activity type corresponds to a range of AI that must be determined first. In other words, the threshold values of these intensity intervals are necessary to classify activities. Once the cutoff values of the PA metrics are selected, several models are available for classification. Among the 75 classification studies reviewed, researchers opted for the threshold-based method (32/75, 43%), machine learning (ML; 32/75, 43%), regression (11/75, 15%), and the hidden Markov model (HMM; 6/75, 8%). These numbers are indicated above the edges connecting the corresponding nodes in Figure 4.

Threshold-based methods are commonly used to identify activities with different intensities after specific cutoff points of PA metrics are determined. These cutoff values are often established by the receiver operating characteristic (ROC) curves based on metrics transformed from the triaxial accelerations and aggregated over a specific period, such as a week. The AI values are then categorized into multiple levels or ranges. For instance, this approach was applied in studies monitoring the daily behaviors of children who were instructed to engage in specific activities [47-49]. The intensity level of each activity is determined based on its characteristics, followed by the use of an ROC curve to evaluate the differentiation between target behaviors.

Threshold-based classification offers the advantage of easy calculation and interpretation. However, this method may lack robustness because AI levels can be affected by various factors, such as the placement of the device on the body and differences among populations, and are highly dependent on the individual, limiting their generalizability [50,51,465]. If a large amount of data is collected from each individual during specific activities, individual-specific thresholds can be established and used to classify this individual’s behavior. This generalization can be particularly useful when considering PA as a form of treatment.

### Activity Classification Studies With Regression Models

Rather than determining thresholds using ROC curves, several studies opt for linear regression models [44,50,51]. Such models allow for individualized threshold values, selected to correspond to a predetermined energy expenditure through a linear relationship established in the regression model. These values are then used in classification tasks, particularly beneficial for those considering PA for older adults or individuals with injuries or diseases [44,50,51]. The rationale behind this design is that the range of physical intensity may differ in populations with specific conditions, necessitating the determination of thresholds tailored to their characteristics. Therefore, these studies first apply linear regression to establish the threshold values between light activity and MVPA in settings such as wheelchair use or treadmill exercise and subsequently evaluate classification using these derived threshold values.

The use of the study-specific thresholds for PA metrics helps mitigate the influence of sampling variation and allows for the incorporation of confounding factors such as age, gender, and health status when establishing the cutoff values. Consequently, the conclusions drawn from such studies are more reliable and applicable to research involving similar populations. However, conducting this type of study requires a larger sample size and repeated measurements of PA to establish, calibrate, and validate the threshold values.

### Activity Classification Studies With ML Models

To avoid the determination of thresholds a priori, other studies have formulated activity classification as a task of activity recognition and resolved this task with supervised ML approaches [13,44,52,53,478-480] or unsupervised methods such as those using k-means and Gaussian mixture models [54]. Almost half (32/75, 43%) of the classification studies reviewed here adopted the ML approach (Figure 4). ML tools, including decision trees, random forest, logistic regression, naive Bayes, and multilayer neural networks, have been used to perform recognition among walking, jogging, going upstairs, going downstairs, sitting, standing, and lying at rest [13,52]. Except for 2 (2/32, 6%) studies based on the National Health and Nutrition Examination Survey [54,317], 2 (6%) on children or adults [53,480], 2 (6%) on older adults (aged >60 and >70 years, respectively [55,56]), and 2 (6%) on participants with diseases (spinal cord injury [51] and cognitive impairment [57]), the remaining articles (24/32, 75%) either used public data sets, such as the experimental data collected during the DARPA’98 Intrusion Detection Evaluation program [478], or recruited convenient samples, such as community residents aged between 20 and 89 years [52], to investigate whether ML algorithms can differentiate between activity types and intensities across groups. In addition, in 7 (22%) out of the 32 articles, participants were asked to wear the device for 1 to 14 days to record their free-living activities, and the data were used to cluster activity types, intensities, or sleep-wake stages. In another 23 (72%) articles, participants were asked to perform specific movements for 30 to 60 minutes, and the data were used to differentiate specific activities.

The performance of these tools was compared using criteria such as *F*_1_-scores, confusion matrices, and accuracy rates. No consistent conclusion of the best tool could be drawn. A logistic regression model with or without cross-validation offered the best performance in some studies [478,480], while multilayer neural networks performed the best in others [479].

Several factors may have affected the inconsistency in performance. First, the sample sizes in these studies were not large, potentially resulting in greater sampling variation and susceptibility to individual variability. Moreover, the ML approaches typically assumed independence among activity data for each epoch, overlooking potential correlations that may have existed. Such interdependencies and associations within the data can be essential when tackling classification problems [481]. In addition, it is worth noting that when identifying specific activities, using individual’s directional accelerations may yield better results compared to using the transformed metric of the triaxial accelerations [482].

### Activity Classification Studies With HMMs

Another classification approach that does not rely on predetermined thresholds is the HMM. Widely used in analyzing time series or correlated data, the HMM originated in the field of speech recognition, where it demonstrated effectiveness in handling sequences of feature vectors. On the basis of augmenting the Markov chain, the fundamental assumption of the HMM is that the observed events depend on hidden states that are not directly observable. A typical HMM consists of essential components, including distinct observations, hidden states, a state transition model, emission probabilities, and an initial state distribution. These components collectively contribute to the modeling and analysis of underlying processes captured by the HMM [29,58-60,483,484].

The application steps for the HMM can be summarized as follows. The first step involves conducting a study to collect labeled data consisting of accelerometer counts and their corresponding activities. These labeled data are then used to estimate the parameters in the HMM classification model. Next, the trained model is applied to unlabeled activity data to estimate the activity type with a probability [29,58-60,483,484]. In the context of activity recognition, the HMM is constructed in 2 different ways. The first one involves a single HMM model for all types of activities to be classified [29,58-60,483,484]. The second one builds an HMM for each specific activity, taking each activity as a distinct entity [483]. These 2 approaches represent different strategies in activity recognition.

The use of the HMM comes with both advantages and limitations. One of the advantages is its ability to leverage the inherent autocorrelation in temporally close activities, which enhances estimation strength in classification tasks [59]. Consequently, the HMM tends to achieve a higher accuracy in classifying types of activities compared to threshold-based methods [484]. Second, HMM provides an estimated probability for each activity label, offering a quantification of uncertainty in estimation. However, parametric HMMs can be computationally expensive due to the large number of parameters involved. In addition, a substantial amount of training data is typically required to ensure stability in parameter estimation. Furthermore, determining the optimal hyperparameter settings before training the model can pose challenges [485].

### Studies on the Association Between PA and Health Conditions With Regression Models

**Figure 5:**
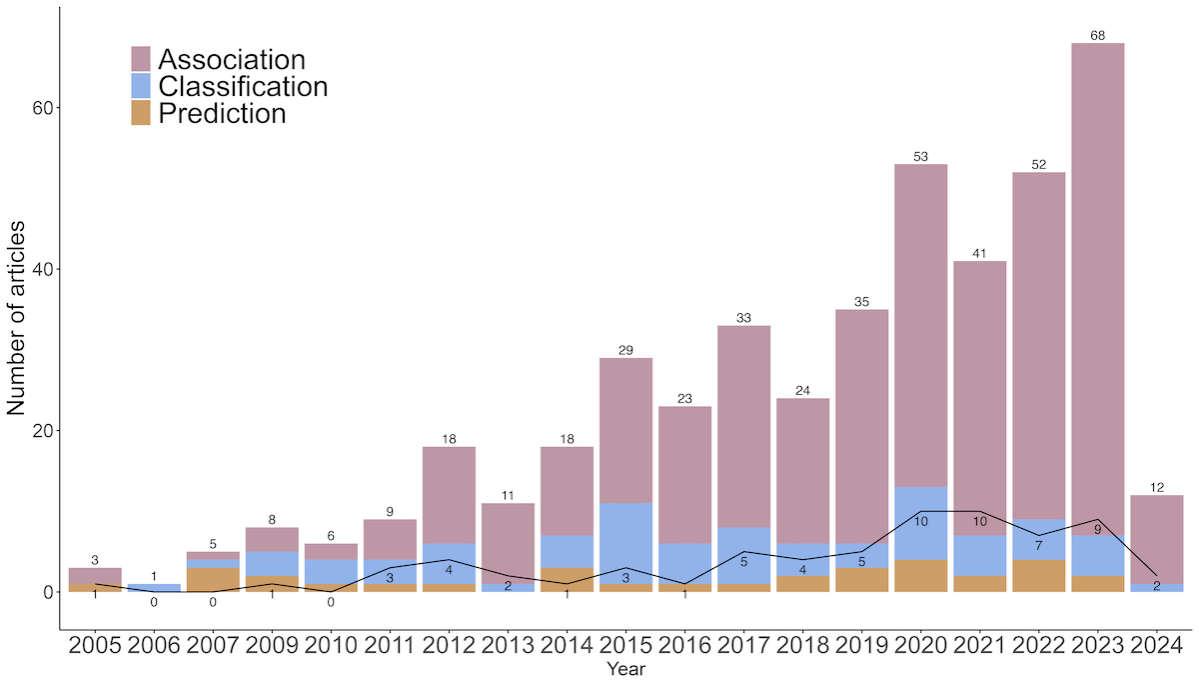
The value on the top of each bar is the sum of the number of articles per topic per year. The solid black line indicates the number of articles considering longitudinal patterns in physical activity (PA).

In addition to classifying activities, there has been a growing emphasis on researching the relationship between PA and health outcomes. Figure 5 illustrates the distribution of articles over the years, with association research consistently comprising the majority (Figure 5). Specifically, association research accounts for 79.9% (342/428) of the reviewed literature (Figure 3 and Multimedia Appendix 3). The studied health outcomes encompass a wide range, including cardiovascular health [61,62], metabolic markers [486], mental well-being [63,64], chronic disease risk [5], and overall mortality [65,66]. By examining the relationship between PA and health outcomes, scientists can gain insights into the potential benefits and mechanisms through which PA impacts overall well-being and disease prevention. Such studies highlight the broader implications and significance of PA in promoting optimal health while controlling confounding factors.

When investigating and interpreting the association between PA and health conditions, regression models are commonly used as powerful tools. The results can provide evidence to identify protective or risk factors of health conditions. In addition, the regression approach can be directly generalized to health outcomes with either discrete or continuous values. Depending on whether the PA metrics are summary statistics over a period such a week or are included as time series in analysis, these regression models can be grouped into the ones using summary-type PA metrics as longitudinal PA and ones emphasizing PA temporal trajectories in FDA, as discussed in subsequent sections.

### Regression Models With Summary PA Metrics

In regression models of this type, the primary aim is to evaluate the impact of overall PA on specific health outcomes. Therefore, summary PA metrics are frequently adopted as explanatory variables. One commonly used summary statistic is the time spent in MVPA per day or week. This metric represents the duration of activities with higher intensity. With this time of MVPA included as a predictor in regression models, studies have revealed beneficial effects of PA on various health conditions, such as healthy aging [487] and reduced frailty [67]. Similar predictors include the times spent in each of the different specified levels of activity (such as low PA, vigorous PA, and sedentary time). Because these variables are correlated with each other, multivariate pattern analysis to handle the multicollinearity was used to examine their association with metabolic health measures such as the cardiometabolic variables [68,69]. Another study used a quantile regression model with the Pittsburgh Fatigability Scale score as the outcome variable and considered the 24-hour rest-activity rhythm as a summary of PA to examine its association with physical fatigability in older adults [70].

Depending on the outcome variable, either a linear regression model or a generalized linear model can be included for analysis, with or without random effects for individual influence. When the health outcome is a time-to-event measurement, a Cox regression model would be used to assess the association. Cox regression models are used for survival analysis focusing on the estimated hazard ratios of predictor variables (eg, PA levels) and the time-to-event outcomes (eg, disease occurrence or mortality). Examples include the incidence of cardiovascular disease [71,72] and all-cause mortality [65,73]. By adjusting for covariates in the model, such as age, sex, smoking status, and comorbidities, researchers can better evaluate the impact of PA on the survival time of the outcome.

The regression models described earlier provide a direct interpretation of the relationship between overall PA patterns and health conditions. However, they do not account for temporal variations, such as longitudinal trends, as the variable can indicate PA changes across time. In other words, these models overlook the variability in time inherent in the original PA metrics such as ENMO, angle-z, and AI. To address this limitation, alternative regression models designed for longitudinal or functional data would offer a more suitable approach.

### Regression Models With PA Metrics as Repeated Measurements

When the study objective is to examine the impact of PA temporal patterns on health outcomes, the identification of trends across time and the use of PA metrics indexed by time are essential. Extracting information from repeatedly measured PA is crucial for achieving this goal. Therefore, the modeling approach must account for correlation in the longitudinally measured metrics. Various statistical models for longitudinal data analysis have been developed to incorporate within-person variation, including repeated-measures ANOVA and mixed-effects models. These models were used in 65 (19%) out of the 342 association studies reviewed, as indicated by the line connecting these 2 nodes in Figure 4.

Repeated-measures ANOVA is a statistical method primarily used to analyze differences among ≥3 dependent samples, with the assumption that different individuals are independent from each other, while different visits or time points for the same individual exhibit correlation. For instance, in a school-based behavior intervention study, 2-way ANOVA was applied to analyze the effect of treatment, time, and their interaction on various PA variables, such as the intensity, frequency, and duration of PA [488]. These PA variables were considered repeated measurements, as they were collected before and after the intervention. This study concluded that the intervention positively impacted the psychological constructs and the PA levels of the adolescents involved. Similarly, in a study on children’s motor skills, repeated-measures multivariate ANOVA (MANOVA) was used to explore skill competence and PA [489].

The calculation of the covariance matrix in repeated-measures MANOVA assumes compound symmetry, implying that variances are equal across time points, and the correlations between any 2 time points are constant. However, this assumption may not hold true if the study spans a long period. Other requirements for repeated-measures MANOVA include complete cases, where no data are missing, and covariates are time-independent. Both conditions may be compromised due to factors such as loss to follow-up, device malfunction or misplacement, or variations in human PA rhythms.

The linear mixed-effects model and GLMM can accommodate continuous or discrete longitudinal response variables. The response variable can be the time spent in various levels of PA, in MVPAs across seasons, or in categories of sleep [74,75]. In a bidirectional association study on adolescents, the linear mixed-effects model was used to assess the effect of night sleep on the PA performed the following day, and the GLMM investigated the effect of daytime PA on night sleep [74]. Both models used the random intercept to account for the day-to-day association at the individual level while adjusting for demographic variables as the fixed effects. When modeling the within-person correlation, some studies chose to model via the correlation matrix, rather than the random effect. For instance, a generalized linear model was used in a cohort study of aging workers to assess the effect of neighborhood socioeconomic disadvantages and greenness on leisure-time PA [76]. The time spent was monitored during working and nonworking days. The within-person variation in the repeatedly measured PA time was included in the covariance structure, in contrast to the person-specific or cluster-specific random effect in mixed-effects models. The quantified association can then be estimated by generalized estimating equations, as demonstrated in studies of frailty and daily PA [77,490].

### Regression Models With PA Metrics as Functions of Time

An alternative approach to modeling PA recognizes it as a continuum and considers the collected PA measurements as discretized observations from a continuous curve. This involves applying FDA in regression models to establish association. Unlike the amplitudes from the FFTs or the scores from FPCA discussed earlier, FDA-based regression models can capture time-varying relationships between 2 variables, both of which can be functions of time. The use of this approach has gained much attention (3, 0.9% out of 342 association studies in Figure 4). Functional regression models can be categorized into three groups, depending on whether the response or covariates are functional or vector data: (1) functional responses with functional covariates, (2) functional responses with scalar covariates, and (3) scalar responses with functional covariates [491].

Function-on-function regression is the model for a functional response involving ≥1 functional covariates [38]. The formula for this model can be expressed as follows:

*y_i_*(*t*) = β_0_(*t*) +*∫*β(*t*)*x_i_*(*t*)*dt* + ε*_i_*(*t*) **(6)**

Here, the response, covariate, and coefficient are all time-varying functions. In a study investigating the association between PA and lung function, both the flow-volume curve (FVC) and PA of recruited smokers and nonsmokers were monitored through time [38]. The FVC, measured in mL, indicates the volume of air one can blow out after full inspiration. The FVC was denoted as *y_i_*(*t*), and the PA of the *i*-th person monitored through time was denoted as *x_i_*(*t*). This model allows researchers to investigate how the functional response changes in relation to ≥1 functional covariates, providing valuable insights into the complex associations between functional data.

Function-on-scalar regression (FoSR) allows for the analysis of the relationship between a functional response and scalar covariate. It enables researchers to examine how functional predictors, such as activity patterns, relate to scalar covariates, such as health indicators or disease risk [78,466,468,492]. The model equation of FoSR can be expressed as follows:

*y_i_*(*t*) = β_0_(*t*) + β_1_(*t*)*x_i_* + ε*_i_*(*t*) **(7)**

Here, the only scalar is the covariate *x_i_*_._ FoSR was applied in studies investigating the relationships between 24-hour rest or activity rhythms (the response function *y*) and scalar covariates (*x*) such as clinical characteristics and demographics [42]. In addition, FoSR was used in a study of children’s behavior [468] and a study of activity patterns in individuals with different mental statuses [466]. By leveraging FoSR, these studies evaluated the dynamic relationships between functional responses and scalar covariates over time, providing important findings for understanding various health and behavioral outcomes.

Scalar-on-function regression studies the relationship between a scalar response and functional covariates [493]:

*y_i_* = β_0_ +*∫*β(*t*)*x_i_*(*t*)*dt* + ε*_i_*  **(8)**

Different from the above-mentioned FoSR, the association of interest is now between the scalar response *y_i_* and the functional covariate *x_i_*(*t*). For instance, the percentage of time spent at or above a certain intensity was used as a functional predictor *x_i_*(*t*) to investigate the influence of this PA profile on the same person’s biological aging *y_i_*, a scalar response [41].

The concept of scalar-on-function regression has been extended for types of accelerometer data analysis. For example, the scalar-on-time-by-distribution regression model was proposed to study the association between the scalar response and temporally local distributional information in wearable data [467]. In another study, researchers introduced the scalar-on-quantile-function regression model, which used the user-specific quantile function as a predictor to analyze the distributional nature of accelerometer data [494].

### Prediction of Health Conditions With PA Variables

Of the 428 studies reviewed, 32 (7.5%) were prediction studies (Multimedia Appendix 3), involving the prediction of health-related outcomes or types of PAs [79-82]. Prediction can be derived based on statistical regression models or ML approaches. Once the association between covariates and response is established with the regression models discussed in earlier sections, new responses can be predicted based on newly observed values of predictors [495]. To predict all-cause mortality, a study applied sparse partial least square regression as the ML tool to determine the importance of 21 PA and sedentary behavior features and to construct a composite score based on their relevance [83]. Similarly, PA measures such as the total AC and active-to-sedentary transition probability were identified in logistic regression models as strong stand-alone predictors of 5-year all-cause mortality based on the National Health and Nutrition Examination Survey study, and these PA features can improve the prediction performance of traditional risk factors [43]. The scores derived from FPCA on PA counts were also considered in predicting mortality, sleep efficiency, and decreases in cognition [43,471].

Prediction studies forecasting activity-related energy expenditure are also prevalent [496-499]. Many studies of this category have relied on the explained variance *R*^2^ from linear Pearson correlation analysis or the nonlinear Spearman rank correlation to determine the interpretability of PA variables such as step counts per day or VM counts per day [496]. It is important to note that such predictions are often population specific [84,497-500], and caution is warranted when generalizing these results to children, adults, and patients.

## Discussion

### Principal Findings

There has been a notable increase in the use of accelerometer-based wearable devices to study the association between PA and health. These devices, worn in a free-living environment, offer objective and dynamic information about an individual’s activity patterns, allowing researchers to delve into various health issues. Selecting appropriate activity metrics and analytical approaches is crucial once study aims are established. Our review indicates that these aims typically involve classification, association, and prediction, while commonly used PA metrics often comprise VM values. These metrics can be computed as summary statistics to indicate the overall information regarding PA frequency, intensity, and duration. Alternatively, these metrics can be constructed as longitudinal or functional data, offering insights into PA profiles and time-varying effects. This perspective has attracted attention in recent literature, where the emphasis has shifted toward predominant PA trajectories and temporal variation. We anticipate a continued surge in the use of accelerometer-based wearable devices for PA monitoring, alongside the development of more advanced analytical tools to tackle increasingly complex scientific inquiries.

### Limitations

This review did not include comparison and validation studies between different brands of devices and various PA metrics. Such studies may be crucial in determining whether data collected from different sources can be merged and provide information on data calibration methods. The findings from these studies may be essential when integrating various databases is needed. However, this task extends beyond the current scope of this review. Future research could initiate by gathering studies that compare different brands and subsequently apply network meta-analysis methodologies if the number of studies is not small.

Studies about the validation of PA metrics are few because this requires a gold standard that may not be easy to derive or set up. Studies that relied on self-report questionnaires may draw criticisms of recall bias and lack of reliability. The comparison between different PA metrics is not straightforward either. One solution would be to wear multiple devices that record different metrics and examine their consistency. Nevertheless, as long as the same device is worn by all participants in the study and the number of participants is large, the analysis of PA metrics can still offer insights into the effect of PA on outcomes of interest.

Other limitations of this review pertain to the choice of keywords used to screen articles. Because “physical activity” was a required phrase, articles containing only the word “activity” were excluded. For instance, studies focusing on sleep quality variation or sleep disturbances monitored by wearables may not be included if they did not emphasize the pattern or intensity of PA. However, research using accelerometers to detect sleep quality and efficiency, particularly for evaluating the association between sleep and cognition functions or chronic diseases, has garnered significant interest in recent years [471,492,501,502]. In addition, this review did not categorize articles based on study designs. Further categorizing them into cross-sectional, observational, or clinical studies could provide insights into the necessity and application of the analytical tools adopted in these studies. Similarly, this review did not focus on the use of wearables in clinical settings. However, a quick comparison of the selected articles from 2005 to 2009 with those from 2022 to 2023 revealed that only 2 (15%) out of 13 studies in the former period used accelerometers in clinical settings, while 23 (19.7%) out of 117 studies in the latter period did so. This difference demonstrates the increasing acceptance and adoption of accelerometers within the clinical community.

### Conclusions

PA has been shown to benefit human health and prevent diseases, and wearable accelerometers offer a cost-efficient means to monitor an individual’s daily PA. Data generated by these wearable devices can be used as either summary-type PA metrics or continuous PA functions in ML algorithms or statistical models to address scientific questions regarding the effects of various PAs in digital health studies. Such studies can focus on increasing or maintaining cognitive functions in participants with neurodegenerative conditions, decreasing the risk of chronic diseases such as cardiovascular diseases, and maintaining physical fitness in general populations. Moreover, research on the use of PA for personalized health is expected to increase. For instance, studies leveraging biobank databases that contain both PA and genetic information have been conducted to investigate their impacts on health outcomes such as psychiatric disorders and circadian rhythms [30,85,458] As analytical tools continue to evolve alongside technological advancements, this review delineates the dimensions, developments, and transformations within methodological frameworks providing foundational guidelines for future research on PA and health.

## Appendix

Multimedia Appendix 1 PRISMA (Preferred Reporting Items for Systematic Reviews and Meta-Analyses) checklist.

Multimedia Appendix 2 Key literature search terms and search results.

Multimedia Appendix 3 References of articles included in the review.

## Abbreviations

AC: activity count
AI: activity intensity
ENMO: Euclidean norm minus one
FDA: functional data analysis
FFT: fast Fourier transformation
FoSR: function-on-scalar regression
FPCA: functional principal component analysis
FVC: flow-volume curve
GLMM: generalized linear mixed-effects model
HMM: hidden Markov model
IS: interdaily stability
IV: intradaily variability
MANOVA: multivariate ANOVA
MET: metabolic equivalent of task
ML: machine learning
MVPA: moderate-to-vigorous physical activity
PA: physical activity
PAI: physical activity index
PRISMA-ScR: Preferred Reporting Items for Systematic Reviews and Meta-Analyses extension for Scoping Reviews
ROC: receiver operating characteristic
VM: vector magnitude
