# Supplementary material for: Data Analytics in Physical Activity Studies With Accelerometers: Scoping Review
Source: J Med Internet Res. 2024 Sep 11;26:e59497. doi: 10.2196/59497 (PMC11425027; doi:10.2196/59497)
Supplement: Multimedia Appendix 2 [file jmir_v26i1e59497_app2.docx]

**Search Strategy**

| **Database** | **Search Terms** |
| --- | --- |
| Pubmed | (("physical activity"[Title] NOT questionnaire[Title]) AND (accelerometer*[Title] OR accelerometry[Title] OR "wearable device"[Title] NOT questionnaire[Title/Abstract]))  NOTES: time filter **from 2000 - 2024** |
| IEEE Xplore | ("Document Title": "physical activity" AND (accelerometer* OR "wearable device"))  NOTES: time filter **from 2000 - 2024** |
| ACM Digital Library | ([[Title: "physical activity"]] AND [[Title: "wearable device"] OR [Title: , acceleromet*]] AND [E-Publication Date: (01/01/2000 TO 02/29/2024)] |
