# Supplementary material for: Data Analytics in Physical Activity Studies With Accelerometers: Scoping Review
Source: J Med Internet Res. 2024 Sep 11;26:e59497. doi: 10.2196/59497 (PMC11425027; doi:10.2196/59497)
Supplement: Multimedia Appendix 3 [file jmir_v26i1e59497_app3.docx]

**This file contains four lists:**

**articles included in the review (n=428),**

**articles categorized as classification studies (n=75),**

**articles categorized as association studies (n=342), and**

**articles categorized as prediction studies (n=32).**

**Articles included in the Review (n=428):**

1. Aadland E, Andersen LB, Resaland GK, Kvalheim OM. Interpretation of multivariate association patterns between multicollinear physical activity accelerometry data and cardiometabolic health in children—a tutorial. Metabolites. 2019 Jul;9(7):129. PMID: 31269708. doi: 10.3390/metabo9070129.

2. Aadland E, Kvalheim OM, Anderssen SA, Resaland GK, Andersen LB. Multicollinear physical activity accelerometry data and associations to cardiometabolic health: challenges, pitfalls, and potential solutions. Int J Behav Nutr Phys Act. 2019 Dec;16(1):74. PMID: 31455305. doi: 10.1186/s12966-019-0836-z.

3. Aadland E, Kvalheim OM, Hansen BH, Kriemler S, Ried-Larsen M, Wedderkopp N, et al. The multivariate physical activity signature associated with metabolic health in children and youth: an international children’s accelerometry database (ICAD) analysis. Prev Med. 2020 Dec;141:106266. PMID:33022325. doi: 10.1016/j.ypmed.2020.106266.

4. Aadland E, Steene-Johannessen J. The use of individual cut points from treadmill walking to assess free-living moderate to vigorous physical activity in obese subjects by accelerometry: is it useful? BMC Med Res Methodol. 2012 Nov;12(1):172. PMID:23152980. doi: 10.1186/1471-2288-12-172.

5. Agarwal V, Smuck M, Shah NH. Quantifying the relative change in physical activity after total knee arthroplasty using accelerometer based measurements. AMIA Jt Summits Transl Sci Proc. 2017 Jul;2017:463–472. PMID:28815146.

6. Agbaje AO. Associations of accelerometer‐based sedentary time, light physical activity and moderate‐to‐vigorous physical activity with resting cardiac structure and function in adolescents according to sex, fat mass, lean mass, BMI, and hypertensive status. Scand Med Sci Sports. 2023 Aug;33(8):1399–1411. PMID: 37035905. doi: 10.1111/sms.14365.

7. Agbaje AO. Mediating effect of fat mass, lean mass, blood pressure and insulin resistance on the associations of accelerometer-based sedentary time and physical activity with arterial stiffness, carotid IMT and carotid elasticity in 1574 adolescents. J Hum Hypertens. 2024 Feb. PMID: 38409590. doi: 10.1038/s41371-024-00905-6.

8. Agbaje AO, Perng W, Tuomainen TP. Effects of accelerometer-based sedentary time and physical activity on DEXA-measured fat mass in 6059 children. Nat Commun. 2023 Dec;14(1):8232. PMID: 38086810. doi: 10.1038/s41467-023-43316-w.

9. Agiovlasitis S, Ballenger BK, Schultz EE, Du Q, Motl RW. Calibration of hip accelerometers for measuring physical activity and sedentary behaviours in adults with down syndrome. J intellect Disabil Res. 2023 Feb;67(2):172–181. PMID: 36514268. doi: 10.1111/jir.13002.

10. Aguiar B, Silva J, Rocha T, Carneiro S, Sousa I. Monitoring physical activity and energy expenditure with smartphones. IEEE-EMBS International Conference on Biomedical and Health Informatics (BHI). 2014 Jun 01-04; Valencia, Spain. New York: IEEE; 2014. doi: 10.1109/BHI.2014.6864451.

11. Ahn HJ, Choi EK, Rhee TM, Choi J, Lee KY, Kwon S, et al. Accelerometer-derived physical activity and the risk of death, heart failure, and stroke in patients with atrial fibrillation: a prospective study from UK Biobank. Br J Sports Med. 2024 Feb;bjsports-2023-106862. PMID: 38418213. doi: 10.1136/bjsports-2023-106862.

12. Li A, Ji L, Wang S, Wu J. Physical activity classification using a single triaxial accelerometer based on HMM. IET International Conference on Wireless Sensor Network 2010 (IET-WSN 2010). 2010 Nov 15-17; Beijing, China. London: IET; 2010. doi: 10.1049/cp.2010.1045.

13. Aittasalo M, Vähä-Ypyä H, Vasankari T, Husu P, Jussila AM, Sievänen H. Mean amplitude deviation calculated from raw acceleration data: a novel method for classifying the intensity of adolescents’ physical activity irrespective of accelerometer brand. BMC Sports Sci Med Rehabil. 2015 Aug;7(1):18. PMID: 26251724. doi: 10.1186/s13102-015-0010-0.

14. Ajja R, Wikkeling-Scott LF, Brazendale K, Hijazi R, Abdulle A. Accelerometer measured physical activity patterns of children during segmented school day in Abu Dhabi. BMC Pediatr. 2021 Apr;21(1):182. PMID: 33865344. doi: 10.1186/s12887-021-02639-7.

15. Albrecht BM, Stalling I, Recke C, Bammann K. Accelerometer-assessed outdoor physical activity is associated with meteorological conditions among older adults: cross-sectional results from the OUTDOOR ACTIVE study. PLoS One. 2020 Jan;15(1):e0228053. PMID: 31978178. doi: 10.1371/journal.pone.0228053.

16. Ali H, Messina E, Bisiani R. Subject-dependent physical activity recognition model framework with a semi-supervised clustering approach. 2013 European Modelling. 2013 Nov 20-22; Symposium Manchester, United Kingdom. New York: IEEE; 2013. doi: 10.1109/EMS.2013.7.

17. Allen CL, Montes E, Hoang T, Romo T, Peña J, Navarro J. Can stereotype threat and lift visual messages affect subsequent physical activity? Evidence from a controlled experiment using accelerometers. Health Commun. 2023 Nov;1–12. PMID: 37941378.

18. Alsamman RA, Shousha TM, Faris ME, Abdelrahim DN, Arumugam A. Association of sociodemographic, anthropometric, and sleep quality factors with accelerometer-measured sitting and physical activity times among Emirati working women during the COVID-19 pandemic: A cross-sectional study. Womens Health. 2024 Jan-Dec;20:17455057231225539. PMID: 38279820. doi: 10.1177/17455057231225539.

19. Anastasopoulou P, Tansella M, Stumpp J, Shammas L, Hey S. Classification of human physical activity and energy expenditure estimation by accelerometry and barometry. 2012 Annual International Conference of the IEEE Engineering in Medicine and Biology Society. 2012 Aug 28- Sep 01; San Diego, CA. New York: IEEE; 2012. PMID: 23367406. doi: 10.1109/EMBC.2012.6347471.

20. Andersen MB, Ostenfeld EB, Fuglsang J, Møller M, Daugaard M, Ovesen PG. Maternal prepregnancy body mass index and physical activity during pregnancy assessed by accelerometer. Am J Obstet Gynecol MFM. 2020 Nov;2(4):100182. PMID: 33345908. doi: 10.1016/j.ajogmf.2020.100182.

21. Andersson C, Lyass A, Larson MG, Spartano NL, Vita JA, Benjamin EJ, et al. Physical activity measured by accelerometry and its associations with cardiac structure and vascular function in young and middle‐aged adults. J Am Heart Assoc. 2015 Mar;4(3):e001528. PMID: 25792127. doi: 10.1161/JAHA.114.001528.

22. Appelqvist-Schmidlechner K, Raitanen J, Vasankari T, Kyröläinen H, Häkkinen A, Honkanen T, et al. Relationship between accelerometer-based physical activity, sedentary behavior, and mental health in young Finnish men. Front Public Health. 2022 Feb;10:820852. PMID: 35252097. doi: 10.3389/fpubh.2022.820852.

23. Armstrong B, Covington LB, Hager ER, Black MM. Objective sleep and physical activity using 24-hour ankle-worn accelerometry among toddlers from low-income families. Sleep Health. 2019 Oct;5(5):459–465. PMID: 31171491. doi: 10.1016/j.sleh.2019.04.005.

24. Arteaga D, Donnelly T, Crum K, Markham L, Killian M, Burnette WB, et al. Assessing physical activity using accelerometers in youth with duchenne muscular dystrophy. J Neuromuscul Dis. 2020 Jun;7(3):331–342. PMID: 32417792. doi: 10.3233/JND-200478.

25. Assah FK, Ekelund U, Brage S, Corder K, Wright A, Mbanya JC, et al. Predicting physical activity energy expenditure using accelerometry in adults from Sub‐Sahara Africa. Obesity. 2009 Aug;17(8):1588–1595. PMID: 19247268. doi: 10.1038/oby.2009.39.

26. Augustin NH, Mattocks C, Cooper AR, Ness AR, Faraway JJ. Modelling fat mass as a function of weekly physical activity profiles measured by Actigraph accelerometers. Physiol Meas. 2012 Nov;33(11):1831–1839. PMID: 23110964. doi: 10.1088/0967-3334/33/11/1831.

27. Ayabe M, Kumahara H. Effect of handling breaks on estimation of heart rate responses to bouts of physical activity among young women: An accelerometer research issue. Gait Posture. 2020 Sep;81:1–6. PMID: 32645577. doi: 10.1016/j.gaitpost.2020.06.032.

28. Baldanzi G, Sayols-Baixeras S, Ekblom-Bak E, Ekblom Ö, Dekkers KF, Hammar U, et al. Accelerometer-based physical activity is associated with the gut microbiota in 8416 individuals in SCAPIS. EBioMedicine. 2024 Feb;100:104989. PMID: 38301483. doi: 10.1016/j.ebiom.2024.104989.

29. Ballin M, Antonsson O, Rosenqvist V, Nordström P, Nordström A. Association of dog ownership with accelerometer-measured physical activity and daily steps in 70-year-old individuals: a population-based cross-sectional study. BMC Public Health. 2021 Dec;21(1):2313. PMID: 34933682. doi: 10.1186/s12889-021-12401-4.

30. Bammann K, Thomson NK, Albrecht BM, Buchan DS, Easton C. Generation and validation of ActiGraph GT3X+ accelerometer cut-points for assessing physical activity intensity in older adults. The OUTDOOR ACTIVE validation study. PLoS One. 2021 Jun;16(6):e0252615. PMID: 34081715. doi: 10.1371/journal.pone.0252615.

31. Bangen KJ, Calcetas AT, Thomas KR, Wierenga C, Smith CN, Bordyug M, et al. Greater accelerometer-measured physical activity is associated with better cognition and cerebrovascular health in older adults. J Int Neuropsychol Soc. 2023 Nov;29(9):859–869. PMID: 36789631. doi: 10.1017/S1355617723000140.

32. Banker M, Song PXK. Supervised learning of physical activity features from functional accelerometer data. IEEE J Biomed Health Inform. 2023 Dec;27(12):5710–5721.PMID: 37738184. doi: 10.1109/JBHI.2023.3318205.

33. Baque E, Sakzewski L, Trost SG, Boyd RN, Barber L. Validity of accelerometry to measure physical activity intensity in children with an acquired brain injury. Pediatr Phys Ther. 2017 Oct;29(4):322–329. PMID: 28953176. doi: 10.1097/PEP.0000000000000439.

34. Barker J, Smith Byrne K, Doherty A, Foster C, Rahimi K, Ramakrishnan R, et al. Physical activity of UK adults with chronic disease: cross-sectional analysis of accelerometer-measured physical activity in 96 706 UK Biobank participants. Int J Epidemiol. 2019 Aug;48(4):1167-1174. PMID: 30721947. doi: 10.1093/ije/dyy294.

35. Barrett S, Begg S, O′Halloran P, Kingsley M. The effect of a physical activity coaching intervention on accelerometer-measured sedentary behaviours in insufficiently physically active ambulatory hospital patients. Int J Environ Res Public Health. 2021 May;18(11):5543. PMID: 34067292. doi: 10.3390/ijerph18115543.

36. Baumann S, Groß S, Voigt L, Ullrich A, Weymar F, Schwaneberg T, et al. Pitfalls in accelerometer‐based measurement of physical activity: The presence of reactivity in an adult population. Scand J Med Sci Sports. 2018 Mar;28(3):1056–1063. PMID: 28921747. doi: 10.1111/sms.12977.

37. Baumann S, Guertler D, Weymar F, Bahls M, Dörr M, Van Den Berg N, et al. Do accelerometer-based physical activity patterns differentially affect cardiorespiratory fitness? a growth mixture modeling approach. J Behav Med. 2020 Feb;43(1):99–107. PMID: 31190167. doi: 10.1007/s10865-019-00069-6.

38. Bayartai ME, Määttä J, Karppinen J, Oura P, Takatalo J, Auvinen J, et al. Association of accelerometer-measured physical activity, back static muscular endurance and abdominal obesity with radicular pain and non-specific low back pain. Sci Rep. 2023 May;13(1):7736. PMID: 37173344. doi: 10.1038/s41598-023-34733-4.

39. Bayartai ME, Taulaniemi A, Tokola K, Vähä-Ypyä H, Parkkari J, Husu P, et al. Role of the interaction between lumbar kinematics and accelerometer-measured physical activity in bodily pain, physical functioning and work ability among health care workers with low back pain. J Electromyogr Kinesiol. 2023 Apr;69:102744. PMID: 36680881. doi: 10.1016/j.jelekin.2023.102744.

40. Benadjaoud MA, Menai M, Van Hees VT, Zipunnikov V, Regnaux JP, Kivimäki M, et al. The association between accelerometer-assessed physical activity and respiratory function in older adults differs between smokers and non-smokers. Sci Rep. 2019 Jul;9(1):10270. PMID: 31311982. doi: 10.1038/s41598-019-46771-y.

41. Bergh IH, Van Stralen MM, Grydeland M, Bjelland M, Lien N, Andersen LF, et al. Exploring mediators of accelerometer assessed physical activity in young adolescents in the HEalth In Adolescents study – a group randomized controlled trial. BMC Public Health. 2012 Sep;12(1):814. PMID: 22995043. doi: 10.1186/1471-2458-12-814.

42. Berninger N, Knell G, Gabriel KP, Plasqui G, Crutzen R, Ten Hoor G. Bidirectional day-to-day associations of reported sleep duration with accelerometer measured physical activity and sedentary time among Dutch adolescents: an observational study. J Meas Physical Behav. 2020 Dec;3(4):304–314. PMID: 35665029. doi: 10.1123/jmpb.2020-0010.

43. Biswas A, Chen C, Prince SA, Smith PM, Mustard CA. Daily accelerometer-measured physical activity patterns and associations with cardiometabolic health among Canadian working adults. Health Rep. 2023 Mar;34(3):15-29. PMID: 36921073. doi: 10.25318/82-003-X202300300002-eng.

44. Bonomi AG, Plasqui G, Goris AH, Westerterp KR. Improving assessment of daily energy expenditure by identifying types of physical activity with a single accelerometer. J Appl Physiol. 2009 Sep;107(3):655–661. PMID: 19556460. doi: 10.1152/japplphysiol.00150.2009.

45. Bonomi AG, Goris AH, Yin B, Westerterp KR. Detection of type, duration, and intensity of physical activity using an accelerometer. Med Sci Sports Exerc. 2009 Sep;41(9):1770–1777. PMID: 19657292. doi: 10.1249/MSS.0b013e3181a24536.

46. Browne RAV, Macêdo GAD, Cabral LLP, Oliveira GTA, Vivas A, Fontes EB, et al. Initial impact of the COVID-19 pandemic on physical activity and sedentary behavior in hypertensive older adults: An accelerometer-based analysis. Exp Gerontol. 2020 Dec;142:111121. PMID: 33132145. doi: 10.1016/j.exger.2020.111121.

47. Carlson RH, Huebner DR, Hoarty CA, Whittington J, Haynatzki G, Balas MC, et al. Treadmill gait speeds correlate with physical activity counts measured by cell phone accelerometers. Gait Posture. 2012 Jun;36(2):241–248. PMID: 22475727. doi: 10.1016/j.gaitpost.2012.02.025.

48. Carvalho EV, Reboredo MM, Gomes EP, Teixeira DR, Roberti NC, Mendes JO, et al. Physical activity in daily life assessed by an accelerometer in kidney transplant recipients and hemodialysis patients. Transplant Proc. 2014 Jul-Aug;46(6):1713–1717. PMID: 25131019. doi: 10.1016/j.transproceed.2014.05.019.

49. Cassidy S, Fuller H, Chau J, Catt M, Bauman A, Trenell MI. Accelerometer-derived physical activity in those with cardio-metabolic disease compared to healthy adults: a UK Biobank study of 52,556 participants. Acta Diabetol. 2018 Sep;55(9):975–979. PMID: 29808390. doi: 10.1007/s00592-018-1161-8.

50. Chan JA, Bosma H, Drosinou C, Timmermans EJ, Savelberg H, Schaper N, et al. Association of perceived and objective neighborhood walkability with accelerometer‐measured physical activity and sedentary time in the Maastricht study. Scand Med Sci Sports. 2023 Nov;33(11):2313–2322. PMID: 37489093. doi: 10.1111/sms.14455.

51. Chandler JL, Brazendale K, Beets MW, Mealing BA. Classification of physical activity intensities using a wrist‐worn accelerometer in 8–12‐year‐old children. Pediatr Obes. 2016 Apr;11(2):120–127. PMID: 25893950. doi: 10.1111/ijpo.12033.

52. Chen GC, Qi Q, Hua S, Moon JY, Spartano NL, Vasan RS, et al. Accelerometer-assessed physical activity and incident diabetes in a population covering the adult life span: the Hispanic community health study/study of Latinos. Am J Clin Nutr. 2020 Nov;112(5):1318–1327. PMID: 32910816. doi: 10.1093/ajcn/nqaa232.

53. Chen M, Landré B, Marques-Vidal P, Van Hees VT, Van Gennip ACE, Bloomberg M, et al. Identification of physical activity and sedentary behaviour dimensions that predict mortality risk in older adults: development of a machine learning model in the Whitehall II accelerometer sub-study and external validation in the CoLaus study. EClinicalMedicine. 2023 Jan;55:101773. PMID: 36568684. doi: 10.1016/j.eclinm.2022.101773.

54. Chen T, Honda T, Chen S, Narazaki K, Kumagai S. Dose–response association between accelerometer-assessed physical activity and incidence of functional disability in older Japanese adults: a 6-year prospective study. J Geront A Biol Sci Med Sci. 2020 Sep;75(9):1763–1770. PMID: 32134454. doi: 10.1093/gerona/glaa046.

55. Cheng TS, Brage S, Van Sluijs EMF, Ong KK. Pre-pubertal accelerometer-assessed physical activity and timing of puberty in British boys and girls: the Millennium cohort study. Int J Epidemiol. 2023 Oct;52(5):1316–1327. PMID: 37208864. doi: 10.1093/ije/dyad063.

56. Choe EY, He D, Sun G. Trading-off transit and non-transit physical activity among older people: evidence from longitudinal accelerometer data of a natural experiment study. J Urban Health. 2023 Apr;100(2):408–417. PMID: 36656439. doi: 10.1007/s11524-022-00709-4.

57. Cochrane SK, Chen S, Fitzgerald JD, Dodson JA, Fielding RA, King AC, et al. Association of accelerometry‐measured physical activity and cardiovascular events in mobility‐Limited older adults: The LIFE (lifestyle interventions and independence for elders) study. J Am Heart Assoc. 2017 Dec;6(12):e007215. PMID: 29197830. doi: 10.1161/JAHA.117.007215.

58. Cohn-Schwartz E, Khalaila R. Accelerometer-assessed physical activity and cognitive performance among European adults aged 50+: the mediating effects of social contacts and depressive symptoms. Healthcare. 2022 Nov;10(11):2279. PMID: 36421603. doi: 10.3390/healthcare10112279.

59. Cooper AR, Goodman A, Page AS, Sherar LB, Esliger DW, Van Sluijs EM, et al. Objectively measured physical activity and sedentary time in youth: the international children’s accelerometry database (ICAD). Int J Behav Nutr Phys Act. 2015 Sep;12(1):113. PMID: 26377803. doi: 10.1186/s12966-015-0274-5.

60. Corcoran MP, Chui KK, White DK, Reid KF, Kirn D, Nelson ME, et al. Accelerometer assessment of physical activity and its association with physical function in older adults residing at assisted care facilities. J Nutr Health Aging. 2016 Jul;20(7):752–758. PMID: 27499309. doi: 10.1007/s12603-015-0640-7.

61. Corder K, Sharp SJ, Atkin AJ, Andersen LB, Cardon G, Page A, et al. Age-related patterns of vigorous-intensity physical activity in youth: the international children’s accelerometry database. Prev Med Rep. 2016 May;4:17–22. PMID: 27413656. doi: 10.1016/j.pmedr.2016.05.006.

62. Crouter SE, Flynn JI, Bassett DR. Estimating physical activity in youth using a wrist accelerometer. Med Sci Sports Exerc. 2015 May;47(5):944–951. PMID: 25207928. doi: 10.1249/MSS.0000000000000502.

63. Crouter SE, Oody JF, Bassett DR. Estimating physical activity in youth using an ankle accelerometer. J Sports Sci. 2018 Oct;36(19):2265–2271. PMID: 29517959. doi: 10.1080/02640414.2018.1449091.

64. D’Silva A, Bebb G, Boyle T, Johnson ST, Vallance JK. Demographic and clinical correlates of accelerometer assessed physical activity and sedentary time in lung cancer survivors. Psychooncology. 2018 Mar;27(3):1042–1049. PMID: 29226994. doi: 10.1002/pon.4608.

65. Da Silva IC, Van Hees VT, Ramires VV, Knuth AG, Bielemann RM, Ekelund U, et al. Physical activity levels in three Brazilian birth cohorts as assessed with raw triaxial wrist accelerometry. Int J Epidemiol. 2014 Dec;43(6):1959–1968. PMID: 25361583. doi: 10.1093/ije/dyu203.

66. Da Silva SG, Evenson KR, Da Silva ICM, Mendes MA, Domingues MR, Da Silveira MF, et al. Correlates of accelerometer‐assessed physical activity in pregnancy—the 2015 Pelotas (Brazil) birth cohort study. Scand Med Sci Sports. 2018 Aug;28(8):1934–1945. PMID: 29542188. doi: 10.1111/sms.13083.

67. Dankel SJ, Loenneke JP, Loprinzi PD. Combined associations of muscle-strengthening activities and accelerometer-assessed physical activity on multimorbidity: findings from NHANES. Am J Health Promot. 2017 Jul;31(4):274–277. PMID: 26730562. doi: 10.4278/ajhp.150520-QUAN-894.

68. DasMahapatra P, Chiauzzi E, Bhalerao R, Rhodes J. Free-living physical activity monitoring in adult US patients with multiple sclerosis using a consumer wearable device. Digit Biomark. 2018 Apr;2(1):47–63. PMID: 32095756. doi: 10.1159/000488040.

69. Davoudi A, Ozrazgat-Baslanti T, Tighe PJ, Bihorac A, Rashidi P. Pain and physical activity association in Critically Ill patients. 2020 42nd Annual International Conference of the IEEE Engineering in Medicine & Biology Society (EMBC) Montreal, QC, Canada: IEEE; 2020. p. 5696–5699. doi: 10.1109/EMBC44109.2020.9176227.

70. De Vries EA, Heijenbrok-Kal MH, Van Kooten F, Giurgiu M, Ribbers GM, Van Den Berg-Emons RJG, et al. Unraveling the interplay between daily life fatigue and physical activity after subarachnoid hemorrhage: an ecological momentary assessment and accelerometry study. J Neuroeng Rehabil. 2023 Sep;20(1):127. PMID: 37752550. doi: 10.1186/s12984-023-01241-5.

71. Del Pozo Cruz B, Ahmadi M, Inan‐Eroglu E, Huang B, Stamatakis E. Prospective associations of accelerometer‐assessed physical activity with mortality and incidence of cardiovascular disease among adults with hypertension: the UK Biobank study. J Am Heart Assoc. 2022 Mar;11(6):e023290. PMID: 35253444. doi: 10.1161/JAHA.121.023290.

72. Delgado-Gonzalo R, Celka P, Renevey Ph, Dasen S, Sola J, Bertschi M, et al. Physical activity profiling: activity-specific step counting and energy expenditure models using 3D wrist acceleration. 2015 37th Annual International Conference of the IEEE Engineering in Medicine and Biology Society (EMBC); 2015 Nov 25-29; Milan, Italy. New York: IEEE; 2015. doi: 10.1109/EMBC.2015.7320271.

73. Dennison CA, Legge SE, Bracher-Smith M, Menzies G, Escott-Price V, Smith DJ, et al. Association of genetic liability for psychiatric disorders with accelerometer-assessed physical activity in the UK Biobank. PLoS One. 2021 Mar;16(3):e0249189. PMID: 33770123. doi: 10.1371/journal.pone.0249189.

74. Diaz KM, Krupka DJ, Chang MJ, Kronish IM, Moise N, Goldsmith J, et al. Wrist-based cut-points for moderate- and vigorous-intensity physical activity for the Actical accelerometer in adults. J Sports Sci. 2018 Jan;36(2):206–212. PMID: 28282744. doi: 10.1080/02640414.2017.1293279.

75. Dibben GO, Gandhi MM, Taylor RS, Dalal HM, Metcalf B, Doherty P, et al. Physical activity assessment by accelerometry in people with heart failure. BMC Sports Sci Med Rehabil. 2020 Aug;12(1):47. PMID: 32817798. doi: 10.1186/s13102-020-00196-7.

76. Dijk-Huisman HCV, Senden R, Smeets MHH, Marcellis RGJ, Magdelijns FJH, Lenssen AF. The effect of a smartphone app with an accelerometer on the physical activity behavior of hospitalized patients: a randomized controlled trial. Sensors. 2023 Oct;23(21):8704. PMID: 37960404. doi: 10.3390/s23218704.

77. Diniz-Sousa F, Veras L, Ribeiro JC, Boppre G, Devezas V, Santos-Sousa H, et al. Accelerometry calibration in people with class II-III obesity: energy expenditure prediction and physical activity intensity identification. Gait Posture. 2020 Feb;76:104–109. PMID: 31756665. doi: 10.1016/j.gaitpost.2019.11.008.

78. Do B, Zink J, Mason TB, Belcher BR, Dunton GF. Physical activity and sedentary time among mothers of school-aged children: differences in accelerometer-derived pattern metrics by demographic, employment, and household factors. Women Health Issues. 2022 Sep-Oct;32(5):490–498. PMID: 35491346. doi: 10.1016/j.whi.2022.03.005.

79. Doherty A, Jackson D, Hammerla N, Plötz T, Olivier P, Granat MH, et al. Large scale population assessment of physical activity using wrist worn accelerometers: the UK Biobank study. PLoS One 2017 Feb;12(2):e0169649. PMID: 28146576. doi: 10.1371/journal.pone.0169649.

80. Dohrn IM, Sjöström M, Kwak L, Oja P, Hagströmer M. Accelerometer-measured sedentary time and physical activity—A 15 year follow-up of mortality in a Swedish population-based cohort. J Sci Med Sport. 2018 Jul;21(7):702–707. PMID: 29128418. doi: 10.1016/j.jsams.2017.10.035.

81. Dohrn IM, Welmer AK, Hagströmer M. Accelerometry-assessed physical activity and sedentary time and associations with chronic disease and hospital visits - a prospective cohort study with 15 years follow-up. Int J Behav Nutr Phys Act. 2019 Dec;16(1):125. PMID: 31818303. doi: 10.1186/s12966-019-0878-2.

82. Domazet SL, Tarp J, Thomsen RW, Højlund K, Stidsen JV, Brønd JC, et al. Accelerometer-derived physical activity and sedentary behaviors in individuals with newly diagnosed type 2 diabetes: a cross-sectional study from the Danish nationwide DD2 cohort. Front Sports Act Living. 2023 Jan;4:1089579. PMID: 36761371. doi: 10.3389/fspor.2022.1089579.

83. Domingos C, Picó-Pérez M, Magalhães R, Moreira M, Sousa N, Pêgo JM, et al. Free-living physical activity measured with a wearable device is associated with larger hippocampus volume and greater functional connectivity in healthy older adults: an observational, cross-sectional study in northern Portugal. Front Aging Neurosci. 2021 Nov;13:729060. PMID: 34916921. doi: 10.3389/fnagi.2021.729060.

84. Dougherty EN, Randall I, Haedt-Matt AA, Pila E, Smith K, Wang S, et al. Accelerometer-based physical activity and shape and weight concerns among youth with overweight and obesity: a pilot exploratory ecological momentary assessment study. Child Obes. 2023 May. PMID: 37253094. doi: 10.1089/chi.2022.0236.

85. Douma JAJ, Verdonck-de Leeuw IM, Leemans CR, Jansen F, Langendijk JA, Baatenburg De Jong RJ, et al. Demographic, clinical and lifestyle-related correlates of accelerometer assessed physical activity and fitness in newly diagnosed patients with head and neck cancer. Acta Oncol. 2020 Mar;59(3):342–350. PMID: 31608747. doi: 10.1080/0284186X.2019.1675906.

86. Dugas LR, Kliethermes S, Plange-Rhule J, Tong L, Bovet P, Forrester TE, et al. Accelerometer-measured physical activity is not associated with two-year weight change in African-origin adults from five diverse populations. PeerJ. 2017 Jan;5:e2902. PMID: 28133575. doi: 10.7717/peerj.2902.

87. Duncan MJ, Roscoe CMP, Faghy M, Tallis J, Eyre ELJ. Estimating physical activity in children aged 8–11 years using accelerometry: contributions from fundamental movement skills and different accelerometer placements. Front Physiol. 2019 Mar;10:242. PMID: 30936837. doi: 10.3389/fphys.2019.00242.

88. Edwards MK, Loprinzi PD. Associations between accelerometer-assessed sedentary behavior, physical activity and objectively-measured cardiorespiratory fitness with red blood cell distribution width. Int J Cardiol. 2016 Oct;221:755–758. PMID: 27428316. doi: 10.1016/j.ijcard.2016.07.137.

89. Ehakeem A, Gregson CL, Tobias JH, Lawlor DA. Age at puberty and accelerometer-measured physical activity: findings from two independent UK cohorts. Ann of Hum Biol. 2020 Jun;47(4):391–399. PMID: 32380867. doi: 10.1080/03014460.2019.1707284.

90. Ekblom-Bak E, Börjesson M, Ekblom Ö, Angerås O, Bergman F, Berntsson C, et al. Accelerometer derived physical activity and subclinical coronary and carotid atherosclerosis: cross-sectional analyses in 22 703 middle-aged men and women in the SCAPIS study. BMJ Open. 2023 Nov;13(11):e073380. PMID: 37996228. doi: 10.1136/bmjopen-2023-073380.

91. Ekblom‐Bak E, Börjesson M, Bergman F, Bergström G, Dahlin‐Almevall A, Drake I, et al. Accelerometer derived physical activity patterns in 27.890 middle‐aged adults: the SCAPIS cohort study. Scand Med Sci Sports. 2022 May;32(5):866–880. PMID: 35080270. doi: 10.1111/sms.14131.

92. Ekstedt M, Nyberg G, Ingre M, Ekblom Ö, Marcus C. Sleep, physical activity and BMI in six to ten-year-old children measured by accelerometry: a cross-sectional study. Int J Behav Nutr Phys Act. 2013 Jun;10(1):82. PMID: 23800204. doi: 10.1186/1479-5868-10-82.

93. Elhakeem A, Hannam K, Deere KC, Hartley A, Clark EM, Moss C, et al. Associations of lifetime walking and weight bearing exercise with accelerometer-measured high impact physical activity in later life. Prev Med Rep. 2017 Dec;8:183–189. PMID: 29134173. doi: 10.1016/j.pmedr.2017.10.011.

94. Ellis K, Kerr J, Godbole S, Lanckriet G, Wing D, Marshall S. A random forest classifier for the prediction of energy expenditure and type of physical activity from wrist and hip accelerometers. Physiol Meas. 2014 Dec;35(11):2191–2203. PMID: 25340969. doi: 10.1088/0967-3334/35/11/2191.

95. Evenson KR, Herring AH, Wen F. Accelerometry-assessed latent class patterns of physical activity and sedentary behavior with mortality. Am J Prev Med. 2017 Feb;52(2):135–143. PMID: 28109457. doi: 10.1016/j.amepre.2016.10.033.

96. Evenson KR, Wen F, Herring AH, Di C, LaMonte MJ, Tinker LF, et al. Calibrating physical activity intensity for hip-worn accelerometry in women age 60 to 91years: the women’s health initiative OPACH calibration study. Prev Med Rep. 2015;2:750–756. PMID: 26527313. doi: 10.1016/j.pmedr.2015.08.021.

97. Ezeugwu V, Klaren RE, A Hubbard E, Manns PT, Motl RW. Mobility disability and the pattern of accelerometer-derived sedentary and physical activity behaviors in people with multiple sclerosis. Prev Med Rep. 2015 Apr;2:241–246. PMID: 26844077. doi: 10.1016/j.pmedr.2015.03.007.

98. Fairclough SJ, Rowlands AV, Del Pozo Cruz B, Crotti M, Foweather L, Graves LEF, et al. Reference values for wrist-worn accelerometer physical activity metrics in England children and adolescents. Int J Behav Nutr Phys Act. 2023 Mar;20(1):35. PMID: 36964597. doi: 10.1186/s12966-023-01435-z.

99. Chuang FC, Wang JS, Yang YT, Kao TP. A wearable activity sensor system and its physical activity classification scheme. The 2012 International Joint Conference on Neural Networks (IJCNN); 2012 Jun 10-15; Brisbane, Australia. New York: IEEE; 2012. doi: 10.1109/IJCNN.2012.6252581.

100. Farooq M, Sazonov E. A novel wearable device for food intake and physical activity recognition. Sensors. 2016 Jul;16(7):1067. PMID: 27409622. doi: 10.3390/s16071067.

101. Feinglass J, Lee J, Semanik P, Song J, Dunlop D, Chang R. The effects of daily weather on accelerometer-measured physical activity. J Phys Act Health. 2011 Sep;8(7):934–943. PMID: 21885884. doi: 10.1123/jpah.8.7.934.

102. Fernández-Verdejo R, Alcantara JMA, Galgani JE, Acosta FM, Migueles JH, Amaro-Gahete FJ, et al. Deciphering the constrained total energy expenditure model in humans by associating accelerometer-measured physical activity from wrist and hip. Sci Rep. 2021 Jun;11(1):12302. PMID: 34112912. doi: 10.1038/s41598-021-91750-x.

103. Foldager F, Jørgensen PB, Tønning LU, Petersen ET, Jakobsen SS, Vainorius D, et al. The relationship between muscle power, functional performance, accelerometer-based measurement of physical activity and patient-reported outcomes in patients with hip osteoarthritis: A cross-sectional study. Musculoskele Sci Pract. 2022 Dec;62:102678. PMID: 36335851. doi: 10.1016/j.msksp.2022.102678.

104. Foong YC, Chherawala N, Aitken D, Scott D, Winzenberg T, Jones G. Accelerometer‐determined physical activity, muscle mass, and leg strength in community‐dwelling older adults. J Cachexia Sarcopenia Muscle. 2016 Jun;7(3):275–283. PMID: 27239404. doi: 10.1002/jcsm.12065.

105. Fox FAU, Diers K, Lee H, Mayr A, Reuter M, Breteler MMB, et al. Association between accelerometer-derived physical activity measurements and brain structure: a population-based cohort study. Neurology. 2022 Sep;99(11). PMID: 35918154. doi: 10.1212/WNL.0000000000200884.

106. Francis SL, Simmering JE, Polgreen LA, Evans NJ, Hosteng KR, Carr LJ, et al. Gamifying accelerometer use increases physical activity levels of individuals pre-disposed to type II diabetes. Prev Med Rep. 2021 May;23:101426. PMID: 34178586. doi: 10.1016/j.pmedr.2021.101426.

107. Frehlich L, Turin TC, Doyle-Baker PK, Lang JJ, McCormack GR. Mediation analysis of the associations between neighbourhood walkability and greenness, accelerometer-measured physical activity, and health-related fitness in urban dwelling Canadians. Prev Med. 2024 Jan;178:107792. PMID: 38052331. doi: 10.1016/j.ypmed.2023.107792.

108. Fridolfsson J, Arvidsson D, Ekblom-Bak E, Ekblom Ö, Bergström G, Börjesson M. Accelerometer-measured absolute versus relative physical activity intensity: cross-sectional associations with cardiometabolic health in midlife. BMC Public Health. 2023 Nov;23(1):2322. PMID: 37996871. doi: 10.1186/s12889-023-17281-4.

109. Fridolfsson J, Börjesson M, Ekblom-Bak E, Ekblom Ö, Arvidsson D. Stronger association between high intensity physical activity and cardiometabolic health with improved assessment of the full intensity range using accelerometry. Sensors. 2020 Feb;20(4):1118. PMID: 32085652. doi: 10.3390/s20041118.

110. Frith E, Loprinzi PD. Accelerometer-assessed light-intensity physical activity and mortality among those with mobility limitations. Disabil Health J. 2018 Apr;11(2):298–300. PMID: 28877860. doi: 10.1016/j.dhjo.2017.08.007.

111. Full KM, Moran K, Carlson J, Godbole S, Natarajan L, Hipp A, et al. Latent profile analysis of accelerometer-measured sleep, physical activity, and sedentary time and differences in health characteristics in adult women. PLoS One, 2019 Jun;14(6):e0218595. PMID: 31247051. doi: 10.1371/journal.pone.0218595.

112. Full KM, Whitaker KM, Pettee Gabriel K, Lewis CE, Sternfeld B, Sidney S, et al. Cardiovascular risk and functional burden at midlife: prospective associations of isotemporal reallocations of accelerometer-measured physical activity and sedentary time in the CARDIA study. Prev Med. 2021 Sep;150:106626. PMID: 34019927. doi: 10.1016/j.ypmed.2021.106626.

113. Gába A, Dygrýn J, Mitáš J, Jakubec L, Frömel K. Effect of Accelerometer Cut-Off Points on the Recommended Level of Physical Activity for Obesity Prevention in Children. PLoS One. 2016 Oct;11(10):e0164282. PMID: 27723835. doi: 10.1371/journal.pone.0164282.

114. Gába A, Mitáš J, Jakubec L. Associations between accelerometer-measured physical activity and body fatness in school-aged children. Environ Health Prev Med. 2017 Apr;22(1):43. PMID: 29165128. doi: 10.1186/s12199-017-0629-4.

115. Gao N, Shao W, Salim FD. Predicting personality traits from physical activity intensity. Comput. 2019 Jul;52(7):47–56. doi: 10.1109/MC.2019.2913751.

116. Gao Y, Hua S, Mok Y, Salameh M, Qi Q, Chen G, et al. Joint associations of peripheral artery disease and accelerometry-based physical activity with mortality: the Hispanic community health study/study of Latinos (HCHS/SOL). Atherosclerosis. 2022 Apr;347:55–62. PMID: 35334347. doi: 10.1016/j.atherosclerosis.2022.03.008.

117. Garay JL, Barreira TV, Wang Q, Brutsaert TD. Size at birth and accelerometer‐measured physical activity or sedentary behavior in healthy term‐born adults. Am J Hum Biol. 2022 Jun;34(6):e23717. PMID: 34978113. doi: 10.1002/ajhb.23717.

118. García-Massó X, Serra-Añó P, Gonzalez LM, Ye-Lin Y, Prats-Boluda G, Garcia-Casado J. Identifying physical activity type in manual wheelchair users with spinal cord injury by means of accelerometers. Spinal Cord. 2015 Oct;53(10):772–777. PMID: 25987002. doi: 10.1038/sc.2015.81.

119. Garnotel M, Bastian T, Romero-Ugalde HM, Maire A, Dugas J, Zahariev A, et al. Prior automatic posture and activity identification improves physical activity energy expenditure prediction from hip-worn triaxial accelerometry. J Appl Physiol. 2018 Mar;124(3):780–790. PMID: 29191980. doi: 10.1152/japplphysiol.00556.2017.

120. Ghorbani S, Afshari M, Eckelt M, Dana A, Bund A. Associations between physical activity and mental health in Iranian adolescents during the COVID-19 pandemic: an accelerometer-based study. Children. 2021 Nov;8(11):1022. PMID: 34828736. doi: 10.3390/children8111022.

121. Glass NL, Bellettiere J, Jain P, LaMonte MJ, LaCroix AZ, Women’s Health Initiative. Evaluation of light physical activity measured by accelerometry and mobility disability during a 6-year follow-up in older women. JAMA Netw Open. 2021 Feb;4(2):e210005. PMID: 33620446. doi: 10.1001/jamanetworkopen.2021.0005.

122. Gråstén A, Huhtiniemi M, Hakonen H, Jaakkola T. Development of accelerometer‐based light to vigorous physical activity in fitness profiles of school‐aged children. Scand Med Sci Sports. 2021 Dec;31(12):2343–2355. PMID: 34536979. doi: 10.1111/sms.14056.

123. Gråstén A, Huhtiniemi M, Kolunsarka I, Jaakkola T. Developmental associations of accelerometer measured moderate-to-vigorous physical activity and sedentary time with cardiorespiratory fitness in schoolchildren. J Sci Med Sport. 2022 Nov;25(11):884–889. PMID: 36137921. doi: 10.1016/j.jsams.2022.08.015.

124. Graves JL, Qiao Y, Moored KD, Boudreau RM, Venditti EM, Krafty RT, et al. Profiles of accelerometry-derived physical activity are related to perceived physical fatigability in older adults. Sensors. 2021 Mar 2;21(5):1718. PMID: 33801352. doi: 10.3390/s21051718.

125. Gupta N, Dencker-Larsen S, Lund Rasmussen C, McGregor D, Rasmussen CDN, Thorsen SV, et al. The physical activity paradox revisited: a prospective study on compositional accelerometer data and long-term sickness absence. Int J Behav Nutr Phys Act. 2020 Jul;17(1):93. PMID: 32690043. doi: 10.1186/s12966-020-00988-7.

126. Gupta N, Heiden M, Aadahl M, Korshøj M, Jørgensen MB, Holtermann A. What is the effect on obesity indicators from replacing prolonged sedentary time with brief sedentary bouts, standing and different types of physical activity during working days? a cross-sectional accelerometer-based study among blue-collar workers. PLoS One. 2016 May;11(5):e0154935. PMID: 27187777. doi: 10.1371/journal.pone.0154935.

127. Guthrie N, Bradlyn A, Thompson SK, Yen S, Haritatos J, Dillon F, et al. Development of an accelerometer-linked online intervention system to promote physical activity in adolescents. PLoS One. 2015 May;10(5):e0128639. PMID: 26010359. doi: 10.1371/journal.pone.0128639.

128. Gutiérrez-Hervás A, Cortés-Castell E, Juste-Ruíz M, Palazón-Bru A, Gil-Guillén V, Rizo-Baeza M. Physical activity values in two-to-seven-year-old children measured by accelerometer over five consecutives 24-hour days. Nutr Hosp. 2018 Jun 27;35(3):527-532. PMID: 29974757. doi: 10.20960/nh.1403.

129. Ha L, Wakefield CE, Diaz C, Mizrahi D, Signorelli C, Yacef K, et al. Patterns of physical activity and sedentary behavior in child and adolescent cancer survivors assessed using wrist accelerometry: A cluster analysis approach. Health Informatics J. 2023 Oct-Dec;29(4):14604582231212525. PMID: 37903362. doi: 10.1177/14604582231212525.

130. Haapala EA, Rantalainen T, Hesketh KD, Rodda CP, Duckham RL. Accelerometer-based osteogenic indices, moderate-to-vigorous and vigorous physical activity, and bone traits in adolescents. J Musculoskelet Neuronal Interact. 2022 Dec;22(4):514–523. PMID:36458389.

131. Hachenberger J, Teuber Z, Li YM, Abkai L, Wild E, Lemola S. Investigating associations between physical activity, stress experience, and affective wellbeing during an examination period using experience sampling and accelerometry. Sci Rep. 2023 May;13(1):8808. PMID: 37258597.doi: 10.1038/s41598-023-35987-8.

132. Hager ER, Gormley CE, Latta LW, Treuth MS, Caulfield LE, Black MM. Toddler physical activity study: laboratory and community studies to evaluate accelerometer validity and correlates. BMC Public Health. 2016 Sep;16(1):936. PMID: 27600404. doi: 10.1186/s12889-016-3569-9.

133. Hagströmer M, Kwak L, Oja P, Sjöström M. A 6 year longitudinal study of accelerometer-measured physical activity and sedentary time in Swedish adults. J Sci Med Sport. 2015 Sep;18(5):553–557. PMID: 25277849. doi: 10.1016/j.jsams.2014.07.012.

134. Hajna S, Brage S, Dalton A, Griffin SJ, Jones AP, Khaw KT, et al. Cross-sectional and prospective associations between active living environments and accelerometer-assessed physical activity in the EPIC-Norfolk cohort. Health Place. 2021 Jan;67:102490. PMID: 33321456. doi: 10.1016/j.healthplace.2020.102490.

135. Hale JL, Knell G, Swartz MD, Shiroma EJ, Ellis T, Lee IM, et al. Association of Parkinson’s disease status with accelerometer-derived physical activity and sedentary behavior in older women: The Women’s Health Study (WHS). Prev Med Rep. 2023 Oct;35:102361. PMID: 37584064. doi: 10.1016/j.pmedr.2023.102361.

136. Halloway S, Arfanakis K, Wilbur J, Schoeny ME, Pressler SJ. Accelerometer physical activity is associated with greater gray matter volumes in older adults without Dementia or mild cognitive impairment. J Gerontol B Psychol Sci Soc Sci. 2019 Sep;74(7):1142–1151. PMID: 29432610. doi: 10.1093/geronb/gby010.

137. Halonen JI, Pulakka A, Pentti J, Kallio M, Koskela S, Kivimäki M, et al. Cross-sectional associations of neighbourhood socioeconomic disadvantage and greenness with accelerometer-measured leisure-time physical activity in a cohort of ageing workers. BMJ Open. 2020 Aug;10(8):e038673. PMID: 32801206. doi: 10.1136/bmjopen-2020-038673.

138. Hamasaki H, Noda M, Moriyama S, Yoshikawa R, Katsuyama H, Sako A, et al. Daily physical activity assessed by a triaxial accelerometer is beneficially associated with waist circumference, serum triglycerides, and insulin resistance in Japanese patients with prediabetes or untreated early type 2 diabetes. J Diabetes Res. 2015;2015:526201. PMID: 26064983. doi: 10.1155/2015/526201.

139. Hansen BH, Kolle E, Dyrstad SM, Holme I, Anderssen SA. Accelerometer-determined physical activity in adults and older people. Med Sci Sports Exerc. 2012 Feb;44(2):266–272. PMID: 21796052. doi: 10.1249/MSS.0b013e31822cb354.

140. Hazizi AS, Aina MB, Mohd NM, Zaitun Y, Hamid JJ, Tabata I. Accelerometer-determined physical activity level among government employees in Penang, Malaysia. Malays J Nutr. 2012 Apr;18(1):57-66. PMID: 23713230.

141. Healy GN, Winkler EAH, Brakenridge CL, Reeves MM, Eakin EG. Accelerometer-derived sedentary and physical activity time in overweight/obese adults with type 2 diabetes: cross-sectional associations with cardiometabolic biomarkers. PLoS One. 2015 Mar;10(3):e0119140. PMID: 25775249. doi: 10.1371/journal.pone.0119140.

142. Heitmann KA, Løchen ML, Hopstock LA, Stylidis M, Welde B, Schirmer H, et al. Cross-sectional associations between accelerometry-measured physical activity, left atrial size, and indices of left ventricular diastolic dysfunction: The Tromsø Study. Prev Med Rep. 2021 Mar;21:101290. PMID: 33425668. doi: 10.1016/j.pmedr.2020.101290.

143. Helgadóttir B, Forsell Y, Ekblom Ö. Physical activity patterns of people affected by depressive and anxiety disorders as measured by accelerometers: a cross-sectional study. PLoS One. 2015 Jan;10(1):e0115894. PMID: 25585123. doi: 10.1371/journal.pone.0115894.

144. Hernando C, Hernando C, Collado EJ, Panizo N, Martinez-Navarro I, Hernando B. Establishing cut-points for physical activity classification using triaxial accelerometer in middle-aged recreational marathoners. PLoS One. 2018 Aug;13(8):e0202815. PMID: 30157271. doi: 10.1371/journal.pone.0202815.

145. Hesketh KR, Evenson KR, Stroo M, Clancy SM, Østbye T, Benjamin-Neelon SE. Physical activity and sedentary behavior during pregnancy and postpartum, measured using hip and wrist-worn accelerometers. Prev Med Rep. 2018 Jun;10:337–345. PMID: 29868389. doi: 10.1016/j.pmedr.2018.04.012.

146. Hikihara Y, Tanaka C, Oshima Y, Ohkawara K, Ishikawa-Takata K, Tanaka S. Prediction models discriminating between nonlocomotive and locomotive activities in children using a triaxial accelerometer with a gravity-removal physical activity classification algorithm. PLoS One. 2014 Apr;9(4):e94940. PMID: 24755646. doi: 10.1371/journal.pone.0094940.

147. Ho EC, Hawkley L, Dale W, Waite L, Huisingh-Scheetz M. Social capital predicts accelerometry-measured physical activity among older adults in the U.S.: a cross-sectional study in the National Social Life, Health, and Aging Project. BMC Public Health. 2018 Jun;18(1):804. PMID: 29945588. doi: 10.1186/s12889-018-5664-6.

148. Hooker SA, Masters KS. Purpose in life is associated with physical activity measured by accelerometer. J Health Psychol. 2016 Jun;21(6):962–971. PMID: 25104777. doi: 10.1177/1359105314542822.

149. Hooker SP, Diaz KM, Blair SN, Colabianchi N, Hutto B, McDonnell MN, et al. Association of accelerometer-measured sedentary time and physical activity with risk of stroke among US adults. JAMA Netw Open. 2022 Jun;5(6):e2215385. PMID: 35657625. doi: 10.1001/jamanetworkopen.2022.15385.

150. Hossain SS, Lazar DM, Begum M. Ordinal statistical models of physical activity levels from accelerometer data. Int J Exerc Sci. 2021 Apr;14(7):338–357. PMID:34055179.

151. Hsiao C, Wen CJ, Yen HY, Hsueh MC, Liao Y. Association between accelerometer-measured light-intensity physical activity and cognitive function in older adults. J Nutr Health Aging. 2022 Mar;26(3):230–235. PMID: 35297464. doi: 10.1007/s12603-022-1749-0.

152. Huang W, Hao L, Wu X, Yu X, Cui E, Leroux A. Gender difference in “second-shift” physical activity: new insights from analyzing accelerometry data in a nationally representative sample. SSM Popul Health. 2023 Dec;24:101536. PMID: 37927817. doi: 10.1016/j.ssmph.2023.101536.

153. Hughes RL, Pindus DM, Khan NA, Burd NA, Holscher HD. Associations between accelerometer-measured physical activity and decal microbiota in adults with overweight and obesity. Med Sci Sport Exerc. 2023 Apr;55(4):680–689. PMID: 36728974. doi: 10.1249/MSS.0000000000003096.

154. Huisingh-Scheetz M, Wroblewski K, Kocherginsky M, Huang E, Dale W, Waite L, et al. The relationship between physical activity and frailty among U.S. older adults based on hourly accelerometry data. J Gerontol A Biol Sci Med Sci. 2018 Apr;73(5):622–629. PMID: 29106478. doi: 10.1093/gerona/glx208.

155. Hyde ET, LaCroix AZ, Evenson KR, Howard AG, Anuskiewicz B, Di C, et al. Accelerometer‐measured physical activity and postmenopausal breast cancer incidence in the women’s health accelerometry collaboration. Cancer. 2023 May;129(10):1579–1590. PMID: 36812131. doi: 10.1002/cncr.34699.

156. Hylkema TH, Brouwer S, Kooijman CM, De Vries AJ, Breukelman F, Dekker H, et al. Accelerometer measured sedentary and physical activity behaviors of working patients after total knee arthroplasty, and their compensation between occupational and leisure time. J Occup Rehabil. 2021 Jun;31(2):350–359. PMID: 32946009. doi: 10.1007/s10926-020-09924-9.

157. Ikeda E, Guagliano JM, Atkin AJ, Sherar LB, Ekelund U, Hansen B, et al. Cross-sectional and longitudinal associations of active travel, organised sport and physical education with accelerometer-assessed moderate-to-vigorous physical activity in young people: the international children’s accelerometry database. Int J Behav Nutr Phys Act. 2022 Apr;19(1):41. PMID: 35366914. doi: 10.1186/s12966-022-01282-4.

158. Ishikawa-Takata K, Kaneko K, Koizumi K, Ito C. Comparison of physical activity energy expenditure in Japanese adolescents assessed by EW4800P triaxial accelerometry and the doubly labelled water method. Br J Nutr. 2013 Oct;110(7):1347–1355. PMID: 23544366. doi: 10.1017/S0007114513000603.

159. Izawa KP, Watanabe S, Hiraki K, Morio Y, Kasahara Y, Takeichi N, et al. Determination of the effectiveness of accelerometer use in the promotion of physical activity in cardiac patients: a randomized controlled trial. Arch Phys Med Rehabil. 2012 Jul;93(11):1896–1902. PMID: 22750166. doi: 10.1016/j.apmr.2012.06.015.

160. Jaeschke L, Steinbrecher A, Boeing H, Gastell S, Ahrens W, Berger K, et al. Factors associated with habitual time spent in different physical activity intensities using multiday accelerometry. Sci Rep. 2020 Jan;10(1):774. PMID: 31964962. doi: 10.1038/s41598-020-57648-w.

161. Jago R, Salway R, House D, Walker R, Emm-Collison L, Sansum K, et al. Short and medium-term effects of the COVID-19 lockdowns on child and parent accelerometer-measured physical activity and sedentary time: a natural experiment. Int J Behav Nutr Phys Act. 2023 Apr 27;20(1):42. PMID: 37101270. doi: 10.1186/s12966-023-01441-1.

162. Jain RK, Vokes T. Physical activity as measured by accelerometer in NHANES 2005–2006 is associated with better bone density and trabecular bone score in older adults. Arch Osteoporos. 2019 Mar;14(1):29. PMID: 30826896. doi: 10.1007/s11657-019-0583-4.

163. Janney CA, Fagiolini A, Swartz HA, Jakicic JM, Holleman RG, Richardson CR. Are adults with bipolar disorder active? Objectively measured physical activity and sedentary behavior using accelerometry. J Affect Disord. 2014 Jan;152–154:498–504. PMID: 24095103. doi: 10.1016/j.jad.2013.09.009.

164. Jeng B, Cederberg KLJ, Lai B, Sasaki JE, Bamman MM, Motl RW. Wrist-based accelerometer cut-points for quantifying moderate-to-vigorous intensity physical activity in Parkinson’s disease. Gait Posture. 2022 Jan;91:235–239. PMID: 34749075. doi: 10.1016/j.gaitpost.2021.10.027.

165. Jeran S, Steinbrecher A, Haas V, Mähler A, Boschmann M, Westerterp KR, et al. Prediction of activity-related energy expenditure under free-living conditions using accelerometer-derived physical activity. Sci Rep. 2022 Oct 4;12(1):16578. PMID: 36195647. doi: 10.1038/s41598-022-20639-0.

166. Jin X, Chen Y, Feng H, Zhou M, Chan JWY, Liu Y, et al. Association of accelerometer-measured sleep duration and different intensities of physical activity with incident type 2 diabetes in a population-based cohort study. J Sport Health Sci. 2024 Mar;13(2):222–232. PMID: 36871624. doi: 10.1016/j.jshs.2023.03.001.

167. Jinhyuk Kim, Nakamura T, Kikuchi H, Yoshiuchi K, Yamamoto Y. Co-variation of depressive mood and spontaneous physical activity evaluated by ecological momentary assessment in major depressive disorder. 2014 36th Annual International Conference of the IEEE Engineering in Medicine and Biology Society; 2014 Aug 26-

168. Júdice PB, Hetherington-Rauth M, Northstone K, Andersen LB, Wedderkopp N, Ekelund U, et al. Changes in physical activity and sedentary patterns on cardiometabolic outcomes in the transition to adolescence: international children’s accelerometry database 2.0. J Pediatr. 2020 Oct;225:166-173.e1. PMID: 32553870. doi: 10.1016/j.jpeds.2020.06.018.

169. Jung ME, Locke SR, Bourne JE, Beauchamp MR, Lee T, Singer J, et al. Cardiorespiratory fitness and accelerometer-determined physical activity following one year of free-living high-intensity interval training and moderate-intensity continuous training: a randomized trial. Int J Behav Nutr Phys Act. 2020 Feb;17(1):25. PMID: 32102667. doi: 10.1186/s12966-020-00933-8.

170. Junno JA, Keisu A, Niemelä M, Modarress Julin M, Korpelainen R, Jämsä T, et al. Accelerometer-measured physical activity is associated with knee breadth in middle-aged Finns – a population-based study. BMC Musculoskelet Disord. 2022 May;23(1):517. PMID: 35642051. doi: 10.1186/s12891-022-05475-7.

171. Junttila HE, Vaaramo MM, Huikari SM, Kari JT, Leinonen A, Farrahi V, et al. Association of accelerometer‐measured physical activity and midlife income: a northern Finland birth cohort 1966 study. Scand Med Sci Sports. 2023 Sep;33(9):1765–1778. PMID: 37272147. doi: 10.1111/sms.14421.

172. Jussila AM, Husu P, Vähä-Ypyä H, Tokola K, Kokko S, Sievänen H, et al. Accelerometer-measured physical activity levels and patterns vary in an age- and sex-dependent fashion among Finnish children and adolescents. Int J Environ Res Public Health. 2022 Jun;19(11):6950. PMID: 35682533. doi: 10.3390/ijerph19116950.

173. Kanai M, Izawa KP, Kobayashi M, Onishi A, Kubo H, Nozoe M, et al. Effect of accelerometer-based feedback on physical activity in hospitalized patients with ischemic stroke: a randomized controlled trial. Clin Rehabil. 2018 Aug;32(8):1047–1056. PMID: 29400070. doi: 10.1177/0269215518755841.

174. Kantomaa MT, Tikanmäki M, Kankaanpää A, Vääräsmäki M, Sipola-Leppänen M, Ekelund U, et al. Accelerometer-measured physical activity and sedentary time differ according to education level in young adults. PLoS One. 2016 Jul;11(7):e0158902. PMID: 27403958. doi: 10.1371/journal.pone.0158902.

175. Keller JL, Tian F, Fitzgerald KC, Mische L, Ritter J, Costello MG, et al. Using real-world accelerometry-derived diurnal patterns of physical activity to evaluate disability in multiple sclerosis. J Rehabil Assist Technol Eng. 2022 Jan;9:205566832110673. PMID: 35070348. doi: 10.1177/20556683211067362.

176. Ketels M, Rasmussen CL, Korshøj M, Gupta N, De Bacquer D, Holtermann A, et al. The relation between domain-specific physical behaviour and cardiorespiratory fitness: a cross-sectional compositional data analysis on the physical activity health paradox using accelerometer-assessed data. Int J Environ Res Public Health. 2020 Oct;17(21):7929. PMID: 33137943. doi: 10.3390/ijerph17217929.

177. Khurshid S, Al-Alusi MA, Churchill TW, Guseh JS, Ellinor PT. Accelerometer-derived “weekend warrior” physical activity and incident cardiovascular disease. JAMA. 2023 Jul;330(3):247. PMID: 37462704. doi: 10.1001/jama.2023.10875.

178. Khurshid S, Weng LC, Al-Alusi MA, Halford JL, Haimovich JS, Benjamin EJ, et al. Accelerometer-derived physical activity and risk of atrial fibrillation. Euro Heart J. 2021 Jul;42(25):2472–2483. PMID: 34037209. doi: 10.1093/eurheartj/ehab250.

179. Khurshid S, Weng LC, Nauffal V, Pirruccello JP, Venn RA, Al-Alusi MA, et al. Wearable accelerometer-derived physical activity and incident disease. NPJ Digit Med. 2022 Sep;5(1):131. PMID: 36056190. doi: 10.1038/s41746-022-00676-9.

180. Kijima T, Akai K, Amagasa S, Inoue S, Yamagata S, Ishibashi Y, et al. Accelerometer-measured physical activity and posture among older adults in assisted-living residences. SAGE Open Med. 2024 Jan;12:20503121231220798. PMID: 38186563. doi: 10.1177/20503121231220798.

181. Kim Y, Wijndaele K, Sharp SJ, Strain T, Pearce M, White T, et al. Specific physical activities, sedentary behaviours and sleep as long-term predictors of accelerometer-measured physical activity in 91,648 adults: a prospective cohort study. Int J Behav Nutr Phys Act. 2019 May;16(1):41. PMID: 31064403. doi: 10.1186/s12966-019-0802-9.

182. Kinnunen TI, Tennant PW, McParlin C, Poston L, Robson SC, Bell R. Agreement between pedometer and accelerometer in measuring physical activity in overweight and obese pregnant women. BMC Public Health. 2011 Jun;11(1):501. PMID: 21703033. doi: 10.1186/1471-2458-11-501.

183. Kong S, Park HY, Kang D, Lee JK, Lee G, Kwon OJ, et al. Seasonal variation in physical activity among preoperative patients with lung cancer determined using a wearable Device. J Clin Med. 2020 Jan;9(2):349. PMID: 32012720. doi: 10.3390/jcm9020349.

184. Kos M, Bogdan M, Glynn NW, Harezlak J. Classification of human physical activity based on raw accelerometry data via spherical coordinate transformation. Stat Med. 2020 Sep;39(22):2901–2920. PMID: 32478905. doi: 10.1002/sim.8582.

185. Kretzschmar M, Lin W, Nardo L, Joseph GB, Dunlop DD, Heilmeier U, et al. Association of physical activity measured by accelerometer, knee joint abnormalities, and cartilage T2 measurements obtained from 3T magnetic resonance imaging: data from the osteoarthritis initiative. Arthritis Care Res (Hoboken). 2015 Sep;67(9):1272–1280. PMID: 25777255. doi: 10.1002/acr.22586.

186. Kruisdijk F, Deenik J, Tenback D, Tak E, Beekman AJ, Van Harten P, et al. Accelerometer-measured sedentary behaviour and physical activity of inpatients with severe mental illness. Psychiatry Res. 2017 Aug;254:67–74. PMID: 28456024. doi: 10.1016/j.psychres.2017.04.035.

187. Kulinski JP, Khera A, Ayers CR, Das SR, De Lemos JA, Blair SN, et al. Association between cardiorespiratory fitness and accelerometer-derived physical activity and sedentary time in the general population. Mayo Clin Proc. 2014 Aug;89(8):1063–1071. PMID: 25012770. doi: 10.1016/j.mayocp.2014.04.019.

188. Kumar D, Thanikkal A, Krishnamurthy P, Chen X, Zhang P. Accelerometer-based alcohol consumption detection from physical activity. 2021 17th International Conference on Wireless and Mobile Computing, Networking and Communications (WiMob); 2021 Oct 11-13; Bologna, Italy. New York: IEEE; 2021. doi: 10.1109/WiMob52687.2021.9606257

189. Kuritz A, Mall C, Schnitzius M, Mess F. Physical activity and sedentary behavior of children in afterschool programs: an accelerometer-based analysis in full-day and half-day elementary schools in Germany. Front Public Health. 2020 Sep;8:463. PMID: 32984249. doi: 10.3389/fpubh.2020.00463.

190. Kwon S, Andersen LB, Grøntved A, Kolle E, Cardon G, Davey R, et al. A closer look at the relationship among accelerometer-based physical activity metrics: ICAD pooled data. Int J Behav Nutr Phys Act. 2019 Apr;16(1):40. PMID: 31036032. doi: 10.1186/s12966-019-0801-x.

191. Kwon S, Janz KF, International Children’s Accelerometry Database (ICAD) Collaborators. Tracking of accelerometry-measured physical activity during childhood: ICAD pooled analysis. Int J Behav Nutr Phys Act. 2012 Jun;9:68. PMID: 22676230. doi: 10.1186/1479-5868-9-68.

192. LaCroix AZ, Bellettiere J, Rillamas-Sun E, Di C, Evenson KR, Lewis CE, et al. Association of light physical activity measured by accelerometry and incidence of coronary heart disease and cardiovascular disease in older women. JAMA Netw Open. 2019 Mar;2(3):e190419. PMID: 30874775. doi: 10.1001/jamanetworkopen.2019.0419.

193. Ladlow P, Nightingale TE, McGuigan MP, Bennett AN, Phillip R, Bilzon JLJ. Impact of anatomical placement of an accelerometer on prediction of physical activity energy expenditure in lower-limb amputees. PLoS One. 2017 Oct;12(10):e0185731. PMID: 28982199. doi: 10.1371/journal.pone.0185731.

194. LaMonte MJ, Buchner DM, Rillamas‐Sun E, Di C, Evenson KR, Bellettiere J, et al. Accelerometer‐measured physical activity and mortality in women aged 63 to 99. J Am Geriatr Soc. 2018 May;66(5):886–894. PMID: 29143320. doi: 10.1111/jgs.15201.

195. LaMonte MJ, LaCroix AZ, Nguyen S, Evenson KR, Di C, Stefanick ML, et al. Accelerometer-measured physical activity, sedentary time, and heart failure risk in women aged 63 to 99 years. JAMA Cardiol. 2024 Feb;e235692 PMID: 38381446. doi: 10.1001/jamacardio.2023.5692.

196. LaMonte MJ, Lewis CE, Buchner DM, Evenson KR, Rillamas‐Sun E, Di C, et al. Both light intensity and moderate‐to‐vigorous physical activity measured by accelerometry are favorably associated with cardiometabolic risk factors in older women: the objective physical activity and cardiovascular health (OPACH) study. J Am Heart Assoc. 2017 Oct;6(10):e007064. PMID: 29042429. doi: 10.1161/JAHA.117.007064.

197. Länsitie M, Kangas M, Jokelainen J, Venojärvi M, Vaaramo E, Härkönen P, et al. Association between accelerometer-measured physical activity, glucose metabolism, and waist circumference in older adults. Diabetes Res Clin Pract. 2021 Aug;178:108937. PMID: 34217770. doi: 10.1016/j.diabres.2021.108937.

198. Länsitie M, Kangas M, Jokelainen J, Venojärvi M, Timonen M, Keinänen-Kiukaanniemi S, et al. Cardiovascular disease risk and all-cause mortality associated with accelerometer-measured physical activity and sedentary time ‒ a prospective population-based study in older adults. BMC Geriatr. 2022 Sep;22(1):729. PMID: 36064345. doi: 10.1186/s12877-022-03414-8.

199. Lawman HG, Horn MLV, Wilson DK, Pate RR. A multilevel approach to examining time-specific effects in accelerometer-assessed physical activity. J Sci Med Sport. 2015 Nov;18(6):667–672. PMID: 25245427. doi: 10.1016/j.jsams.2014.09.003.

200. Le Cornu Q, Chen M, Van Hees V, Léger D, Fayosse A, Yerramalla MS, et al. Association of physical activity, sedentary behaviour, and daylight exposure with sleep in an ageing population: findings from the Whitehall accelerometer sub-study. Int J Behav Nutr Phys Act. 2022 Dec;19(1):144. PMID: 36494722. doi: 10.1186/s12966-022-01391-0.

201. Lee IM, Shiroma EJ, Evenson KR, Kamada M, LaCroix AZ, Buring JE. Accelerometer-measured physical activity and sedentary behavior in relation to all-cause mortality: the women’s health study. Circulation. 2018 Jan;137(2):203–205. PMID: 29109088. doi: 10.1161/CIRCULATIONAHA.117.031300.

202. Lee J, Walker ME, Gabriel KP, Vasan RS, Xanthakis V. Associations of accelerometer-measured physical activity and sedentary time with chronic kidney disease: the Framingham heart study. PLoS One. 2020 Jun;15(6):e0234825. PMID: 32542048. doi: 10.1371/journal.pone.0234825.

203. Lee PH. Examining non-linear associations between accelerometer-neasured physical activity, sedentary behavior, and all-cause mortality using segmented cox regression. Front Physiol. 2016 Jun;7:272. PMID: 27445859. doi: 10.3389/fphys.2016.00272.

204. Lee SY, Kim SC, Gim JA, Park SJ, Seo SH, Kim SJ, et al. Accelerometer-derived physical activity analysis of elderly osteoarthritis patients. Musculoskelet Sci Practi. 2023 Aug;66:102808. PMID: 37352763. doi: 10.1016/j.msksp.2023.102808.

205. Leininger B, Schulz C, Gao Z, Bronfort G, Evans R, Pope Z, et al. Accelerometer-determined physical activity and clinical low back pain measures in adolescents with chronic or subacute recurrent low back pain. J Orthop Sports Phys Ther. 2017 Oct;47(10):769–774. PMID: 28898136. doi: 10.2519/jospt.2017.7345.

206. Lemberg GM, Riso EM, Fjørtoft I, Kjønniksen L, Kull M, Mäestu E. School children’s Physical activity and preferred activities during outdoor recess in Estonia: using accelerometers, recess observation, and schoolyard mapping. Children. 2023 Apr;10(4):702. PMID: 37189951. doi: 10.3390/children10040702.

207. Li L, Nakamura T. An epidemiological sleep study based on a large-scale physical activity database. 2019 IEEE 1st Global Conference on Life Sciences and Technologies (LifeTech); 2019 Mar 12-14; Osaka, Japan. New York: IEEE; 2019. doi: 10.1109/LifeTech.2019.8883989.

208. Li S, Howard JT, Sosa ET, Cordova A, Parra-Medina D, Yin Z. Calibrating wrist-worn accelerometers for physical activity assessment in preschoolers: machine learning approaches. JMIR Form Res. 2020 Aug;4(8):e16727. PMID: 32667893. doi: 10.2196/16727.

209. Li T, Zong G, Peng P, Wang S, Cheng B. Accelerometer-measured physical activity and sample-based frailty in older women: does pattern really matter? Front Public Health. 2024 Jan;11:1304279. PMID: 38332942. doi: 10.3389/fpubh.2023.1304279.

210. Li X, Kearney PM, Keane E, Harrington JM, Fitzgerald AP. levels and sociodemographic correlates of accelerometer-based physical activity in Irish children: a cross-sectional study. J Epidemiol Community Health. 2017 Jun;71(6):521–527. PMID: 28130391. doi: 10.1136/jech-2016-207691

211. Liang YY, Feng H, Chen Y, Jin X, Xue H, Zhou M, Ma H, Ai S, Wing YK, Geng Q, Zhang J. Joint association of physical activity and sleep duration with risk of all-cause and cause-specific mortality: a population-based cohort study using accelerometry. Eur J Prev Cardiol. 2023 Jul;30(9):832-843. PMID: 36990109. doi: 10.1093/eurjpc/zwad060.

212. Lin CY, Lin KP, Hsueh MC, Liao Y. Associations of accelerometer-measured sedentary behavior and physical activity with sleep in older adults. J Formos Med Assoc. 2023 Aug;S0929664623002966. PMID: 37586972. doi: 10.1016/j.jfma.2023.08.001.

213. Lin H, Hartley P, Forsyth F, Pilling M, Hobbs FDR, Taylor CJ, et al. Clinical and demographic correlates of accelerometer-measured physical activity in participants enrolled in the OPTIMISE HFpEF study. Eur J Cardiovasc Nurs 2022 Jan;21(1):67–75. PMID: 33837414. doi: 10.1093/eurjcn/zvab028.

214. Lin HP, Lynk N, Moore LL, Cabral HJ, Heffernan KS, Dumas AK, et al. A pragmatic approach to the comparison of wrist-based cutpoints of physical activity intensity for the MotionWatch8 accelerometer in children. PLoS One. 2020 Jun;15(6):e0234725. PMID: 32559207. doi: 10.1371/journal.pone.0234725.

215. Liu M, Gan X, Ye Z, Zhang Y, He P, Zhou C, et al. Association of accelerometer-measured physical activity intensity, sedentary time, and exercise time with incident Parkinson’s disease. NPJ Digit Med 2023 Nov;6(1):224. PMID: 38017114. doi: 10.1038/s41746-023-00969-7.

216. Liu M, Zhang Y, Zhang Y, He P, Zhou C, Ye Z, et al. Association of accelerometer-measured physical activity and its change with progression to chronic kidney disease in adults with type 2 diabetes and overweight/obesity. Br J Sports Med. 2024 Mar;58(6):313–319. PMID: 38320851. doi: 10.1136/bjsports-2023-107564.

217. Long MT, Pedley A, Massaro JM, Hoffmann U, Esliger DW, Vasan RS, et al. Hepatic steatosis is associated with lower levels of physical activity measured via accelerometry. Obesity. 2015 Jun;23(6):1259–1266. PMID: 25959049. doi: 10.1002/oby.21058.

218. Long, Xi, Bin Yin, Ronald M. Single-accelerometer-based daily physical activity classification. 2009 Annual International Conference of the IEEE Engineering in Medicine and Biology Society Minneapolis; 2009 Sep 03-06; Minneapolis, MN, USA New York: IEEE; 2009. PMID: 19965261. doi: 10.1109/IEMBS.2009.5334925.

219. Loprinzi PD. Accelerometer-determined physical activity and mortality in a national prospective cohort study of adults at high risk of a first atherosclerotic cardiovascular disease event. Int J Cardiol. 2016 Jan;202:417–418. PMID: 26432494. doi: 10.1016/j.ijcard.2015.09.061.

220. Loprinzi PD. Accelerometer-determined physical activity and all-cause mortality in a national prospective cohort study of hypertensive adults. J Hypertens. 2016 May;34(5):848–852. PMID: 26828782. doi: 10.1097/HJH.0000000000000869.

221. Loprinzi PD, Addoh O. Accelerometer-determined physical activity and all-cause mortality in a national prospective cohort study of adults post-acute stroke. Am J Health Promot 2018 Jan;32(1):24–27. PMID: 28718295. doi: 10.1177/0890117117720061.

222. Loprinzi PD, Frith E. Accelerometer-assessed physical activity and school absenteeism due to illness or injury among children and adolescents: NHANES 2003 to 2006. Am J Health Promot. 2018 Mar;32(3):571–577. PMID: 29431510. doi: 10.1177/0890117116684241.

223. Loprinzi PD, Gilham B, Cardinal BJ. Association between accelerometer-assessed physical activity and objectively measured hearing sensitivity among U.S. adults with diabetes. Res Q Exerc Sport. 2014 Sep;85(3):390–397. PMID: 25141090. doi: 10.1080/02701367.2014.930404.

224. Loprinzi PD, Joyner C. Accelerometer-determined physical activity and mortality in a national prospective cohort study: Considerations by visual acuity. Prev Med. 2016 Jun;87:18–21. PMID: 26861750. doi: 10.1016/j.ypmed.2016.02.005.

225. Loprinzi PD, Lee H, Gilham B, Cardinal BJ. Association between accelerometer-assessed physical activity and tinnitus, NHANES 2005–2006. Res Q Exerc Sport. 2013 Jun;84(2):177–185. PMID: 23930543. doi: 10.1080/02701367.2013.784840.

226. Loprinzi PD, Sheffield J, Tyo BM, Fittipaldi-Wert J. Accelerometer-determined physical activity, mobility disability, and health. Disabil Health J. 2014 Oct;7(4):419–425. PMID: 25224982. doi: 10.1016/j.dhjo.2014.05.005.

227. Loprinzi PD, Smit E, Lin FR, Gilham B, Ramulu PY. Accelerometer-assessed physical activity and objectively determined dual sensory impairment in US adults. Mayo Clinic Proc. 2013 Jul;88(7):690–696. PMID: 23751983. doi: 10.1016/j.mayocp.2013.04.008.

228. Loprinzi P, Smit E, Lee H, Crespo C, Andersen R, Blair S. The “Fit but Fat” paradigm addressed using accelerometer-determined physical activity data. N Am J Med Sci. 2014 Jul;6(7):295. PMID: 25077076. doi: 10.4103/1947-2714.136901.

229. Loyen A, Clarke-Cornwell AM, Anderssen SA, Hagströmer M, Sardinha LB, Sundquist K, et al. Sedentary time and physical activity surveillance through accelerometer pooling in four European countries. Sports Med. 2017 Jul;47(7):1421–1435. PMID: 27943147. doi: 10.1007/s40279-016-0658-y.

230. Lund K, Larsen MD, Knudsen T, Kjeldsen J, Nielsen RG, Brage S, et al. Physical activity measured by accelerometry in paediatric and young adult patients with inflammatory bowel disease. BMC Gastroenterol. 2022 Jun;22(1):290. PMID: 35672666. doi: 10.1186/s12876-022-02358-y.

231. Lund Rasmussen C, Palarea-Albaladejo J, Korshøj M, Gupta N, Nabe-Nielsen K, Holtermann A, et al. Is high aerobic workload at work associated with leisure time physical activity and sedentary behaviour among blue-collar workers? A compositional data analysis based on accelerometer data. PLoS One. 2019 Jun;14(6):e0217024. PMID: 31170169. doi: 31170169. 10.1371/journal.pone.0217024.

232. Luo M, Yu C, Del Pozo Cruz B, Chen L, Ding D. Accelerometer-measured intensity-specific physical activity, genetic risk and incident type 2 diabetes: a prospective cohort study. Br J Sports Med. 2023 Oct;57(19):1257–1264. PMID: 37277158. doi: 10.1136/bjsports-2022-106653.

233. Ma J, Kim H, Kim J. Isotemporal substitution analysis of accelerometer-derived sedentary behavior and physical activity on cardiometabolic health in Korean adults: a population-based cross-sectional study. Int J Environ Res Public Health. 2021 Oct;18(21):11102. PMID: 34769622. doi: 10.3390/ijerph182111102.

234. Machado-Rodrigues AM, Rodrigues D, Gama A, Nogueira H, Silva MRG, Mascarenhas LP, et al. Tri-axial accelerometer-assessed physical activity and its association with weight status in a sample of elementary-school children. Obes Res Clin Pract. 2023 May;17(3):192–197. PMID: 37087316. doi: 10.1016/j.orcp.2023.04.003.

235. Madimenos FC, Snodgrass JJ, Blackwell AD, Liebert MA, Sugiyama LS. Physical activity in an indigenous Ecuadorian forager‐horticulturalist population as measured using accelerometry. Am J Hum Biol. 2011 Jul;23(4):488–497. PMID: 21538650. doi: 10.1002/ajhb.21163.

236. Maes I, Van Dyck D, Van Cauwenberg J, Mertens L. Age-related differences in the associations of physical environmental factors and psychosocial factors with accelerometer-assessed physical activity. Health Place. 2021 Jan;67:102492. PMID: 33316601. doi: 10.1016/j.healthplace.2020.102492.

237. Malek ME, Norman Å, Elinder LS, Patterson E, Nyberg G. Relationships between physical activity parenting practices and children’s activity measured by accelerometry with children’s activity style as a moderator—a cross sectional study. Children. 2022 Feb;9(2):248. PMID: 35204968. doi: 10.3390/children9020248.

238. Mañas A, Del Pozo-Cruz B, Guadalupe-Grau A, Marín-Puyalto J, Alfaro-Acha A, Rodríguez-Mañas L, et al. Reallocating accelerometer-assessed sedentary time to light or moderate- to vigorous-intensity physical activity reduces frailty levels in older adults: an isotemporal substitution spproach in the TSHA dtudy. J Am Med Dir Assoc. 2018 Feb;19(2):185.e1-185.e6. PMID: 29269096. doi: 10.1016/j.jamda.2017.11.003.

239. Mañas A, Pozo-Cruz BD, Rodríguez-Gómez I, Losa-Reyna J, Rodríguez-Mañas L, García-García FJ, et al. Can physical activity offset the detrimental consequences of sedentary time on frailty? a moderation analysis in 749 older adults measured with accelerometers. J Am Med Dir Assoc. 2019 May;20(5):634-638.e1. PMID: 30738823. doi: 10.1016/j.jamda.2018.12.012.

240. Manglani HR, Phansikar M, Duraney EJ, McKenna MR, Canter R, Nicholas JA, et al. Accelerometry measures of physical activity and sedentary behavior: associations with cognitive functioning in MS. Mult Scler Relat Disord. 2023 Nov;79:104963. PMID: 37690438. doi: 10.1016/j.msard.2023.104963.

241. Mannini A, Sabatini AM. Machine learning methods for classifying human physical activity from on-body accelerometers. Sensors. 2010 Feb;10(2):1154–1175. PMID: 22205862. doi: 10.3390/s100201154.

242. Manns P, Ezeugwu V, Armijo‐Olivo S, Vallance J, Healy GN. Accelerometer‐derived pattern of sedentary and physical activity time in persons with mobility disability: national health and nutrition examination survey 2003 to 2006. J Am Geriatr Soc. 2015 Jul;63(7):1314–1323. PMID: 26173621. doi: 10.1111/jgs.13490.

243. Mardini MT, Bai C, Wanigatunga AA, Saldana S, Casanova R, Manini TM. Age differences in estimating physical activity by wrist accelerometry using machine learning. Sensors. 2021 May;21(10):3352. PMID: 34065906. doi: 10.3390/s21103352.

244. Maslow AL, Colabianchi N. Youth physical activity resource use and activity measured by accelerometry. Am J Health Behav. 2011 Mar-Apr;35(2):219-227. PMID: 21204684. doi: 10.5993/ajhb.35.2.9.

245. Matabuena M, Félix P, Hammouri ZAA, Mota J, Del Pozo Cruz B. Physical activity phenotypes and mortality in older adults: a novel distributional data analysis of accelerometry in the NHANES. Aging Clin Exp Res. 2022 Dec;34(12):3107–3114. PMID: 36183279. doi: 10.1007/s40520-022-02260-3.

246. Matsuzawa R, Matsunaga A, Kutsuna T, Ishii A, Abe Y, Yoneki K, et al. Association of habitual physical activity measured by an accelerometer with high-density lipoprotein cholesterol levels in maintenance Hemodialysis patients. Sci World J. 2013 Dec;2013:780783. PMID: 24453904. doi: 10.1155/2013/780783.

247. Matthews CE, Keadle SK, Troiano RP, Kahle L, Koster A, Brychta R, et al. Accelerometer-measured dose-response for physical activity, sedentary time, and mortality in US adults. Ame J Clini Nutr. 2016 Nov;104(5):1424–1432. PMID: 27707702. doi: 10.3945/ajcn.116.135129.

248. Maylor BD, Edwardson CL, Clarke-Cornwell AM, Davies MJ, Dawkins NP, Dunstan DW, et al. Physical activity assessed by wrist and thigh worn accelerometry and associations with cardiometabolic health. Sensor. 2023 Aug;23(17):7353. PMID: 37687813. doi: 10.3390/s23177353.

249. McCracken LA, Ma JK, Voss C, Chan FH, Martin Ginis KA, West CR. Wrist accelerometry for physical activity measurement in individuals with spinal cord injury—a need for individually calibrated cut-points. Arch Phys Med Rehabil. 2018 Apr;99(4):684–689. PMID: 29222006. doi: 10.1016/j.apmr.2017.10.024.

250. McDevitt B, Connolly J, Duddy D, Doherty R, Condell J. Preliminary investigations of the validity and interinstrument reliability for classification of accelerometer physical activity cut-points against indirect caliometry in healthy adults. 2022 33rd Irish Signals and Systems Conference (ISSC); 2022 Jun 09-10; Cork, Ireland. New York: IEEE; 2022. doi: 10.1109/ISSC55427.2022.9826199.

251. McDonald L, Oguz M, Carroll R, Thakkar P, Yang F, Dhalwani N, et al. Comparison of accelerometer-derived physical activity levels between individuals with and without cancer: a UK Biobank study. Future Oncol. 2019 Nov;15(33):3763–3774. PMID: 31637942. doi: 10.2217/fon-2019-0443.

252. McGarty AM, Penpraze V, Melville CA. Calibration and cross-validation of the ActiGraph wGT3X+ accelerometer for the estimation of physical activity intensity in children with intellectual disabilities. PLoS One. 2016 Oct;11(10):e0164928. PMID: 27760219. doi: 10.1371/journal.pone.0164928.

253. McLellan G, Arthur R, Donnelly S, Buchan DS. Segmented sedentary time and physical activity patterns throughout the week from wrist-worn ActiGraph GT3X+ accelerometers among children 7–12 years old. J Sport Health Sci. 2020 Mar;9(2):179–188. PMID: 32099726. doi: 10.1016/j.jshs.2019.02.005.

254. Melin M, Hagerman I, Gonon A, Gustafsson T, Rullman E. Variability in physical activity assessed with accelerometer is an independent predictor of mortality in CHF patients. PLoS One. 2016 Apr;11(4):e0153036. PMID: 27054323. doi: 10.1371/journal.pone.0153036.

255. Memari AH, Ghaheri B, Ziaee V, Kordi R, Hafizi S, Moshayedi P. Physical activity in children and adolescents with autism assessed by triaxial accelerometry. Pediatr Obes. 2013 Apr;8(2):150–158. PMID: 23042790. doi: 10.1111/j.2047-6310.2012.00101.x.

256. Micklesfield LK, Westgate K, Smith A, Kufe CN, Mendham AE, Lindsay T, et al. Physical activity behaviors of a middle-age south african cohort as determined by integrated hip and thigh accelerometry. Med Sci Sports Exerc. 2022 Sep;54(9):1493–1505. PMID: 35658390. doi: 10.1249/MSS.0000000000002940.

257. Midorikawa T, Tanaka S, Kaneko K, Koizumi K, Ishikawa‐Takata K, Futami J, et al. Evaluation of low‐intensity physical activity by triaxial accelerometry. Obesity. 2007 Dec;15(12):3031–3038. PMID: 18198312. doi: 10.1038/oby.2007.361.

258. Migueles JH, Cadenas-Sanchez C, Alcantara JMA, Leal-Martín J, Mañas A, Ara I, et al. Calibration and cross-validation of accelerometer cut-points to classify sedentary time and physical activity from hip and non-dominant and dominant wrists in older adults. Sensors. 2021 May;21(10):3326. PMID: 34064790. doi: 10.3390/s21103326.

259. Miller NE, Strath SJ, Swartz AM, Cashin SE. Estimating absolute and relative physical activity intensity across Age via accelerometry in adults. J Aging Phys Act. 2010 Apr;18(2):158–170. PMID: 20440028. doi: 10.1123/japa.18.2.158.

260. Mossavar-Rahmani Y, Lin J, Pan S, Song RJ, Xue X, Spartano NL, et al. Characterizing longitudinal change in accelerometry-based moderate-to-vigorous physical activity in the Hispanic community health study/study of Latinos and the Framingham heart study. BMC Public Health. 2023 Aug;23(1):1614. PMID: 37620824. doi: 10.1186/s12889-023-16442-9.

261. Moy ML, Matthess K, Stolzmann K, Reilly J, Garshick E. Free-living physical activity in COPD: assessment with accelerometer and activity checklist. J Rehabil Res Dev. 2009;46(2):277–286. PMID:19533541.

262. Murabito JM, Pedley A, Massaro JM, Vasan RS, Esliger D, Blease SJ, et al. Moderate‐to‐vigorous physical activity with accelerometry is associated with visceral adipose tissue in adults. J Am Heart Assoc. 2015 Mar;4(3):e001379. PMID: 25736442. doi: 10.1161/JAHA.114.001379.

263. Nagayoshi S, Oshima Y, Ando T, Aoyama T, Nakae S, Usui C, et al. Validity of estimating physical activity intensity using a triaxial accelerometer in healthy adults and older adults. BMJ Open Sport Exerc Med. 2019 Oct;5(1):e000592. PMID: 31749982. doi: 10.1136/bmjsem-2019-000592.

264. Nakabazzi B, Wachira LM, Oyeyemi AL, Ssenyonga R, Onywera VO. Parental-perceived home and neighborhood environmental correlates of accelerometer-measured physical activity among school-going children in Uganda. PLOS Glob Public Health. 2021 Dec;1(12):e0000089. PMID: 36962124. doi: 10.1371/journal.pgph.0000089.

265. Nam S, Jeon S, Ash G, Whittemore R, Vlahov D. Racial discrimination, sedentary time, and physical activity in african americans: quantitative study combining ecological Momentary assessment and accelerometers. JMIR Form Res. 2021 Jun;5(6):e25687. PMID: 34096870. doi: 10.2196/25687.

266. Nawab KA, Storey BC, Staplin N, Walmsley R, Haynes R, Sutherland S, et al. Accelerometer-measured physical activity and functional behaviours among people on dialysis. Clin Kidney J. 2020 Aug;14(3):950–958. PMID: 33777379. doi: 10.1093/ckj/sfaa045.

267. Neil-Sztramko SE, Rafn BS, Gotay CC, Campbell KL. Determining activity count cut-points for measurement of physical activity using the Actiwatch2 accelerometer. Physiol Behav. 2017 May;173:95–100. PMID: 28108333. doi: 10.1016/j.physbeh.2017.01.026.

268. Nero H, Benka Wallén M, Franzén E, Ståhle A, Hagströmer M. Accelerometer cut points for physical activity assessment of older adults with Parkinson’s disease. PLoS One. 2015 Sep;10(9):e0135899. PMID: 26332765. doi: 10.1371/journal.pone.0135899.

269. Nguyen S, LaCroix AZ, Hayden KM, Di C, Palta P, Stefanick ML, et al. Accelerometer‐measured physical activity and sitting with incident mild cognitive impairment or probable dementia among older women. Alzheimers Dement. 2023 Jul;19(7):3041–3054. PMID: 36695426. doi: 10.1002/alz.12908.

270. Noor MHM, Salcic Z, Wang KIK. Dynamic sliding window method for physical activity recognition using a single tri-axial accelerometer. 2015 IEEE 10th Conference on Industrial Electronics and Applications (ICIEA); 2015 Nov 23; Auckland, New Zealand. New York: IEEE; 2015. doi: 10.1109/ICIEA.2015.7334092.

271. Nordstrøm M, Hansen BH, Paus B, Kolset SO. Accelerometer-determined physical activity and walking capacity in persons with down syndrome, williams syndrome and prader–willi syndrome. Res Dev Disabil. 2013 Dec;34(12):4395–4403. PMID: 24139709. doi: 10.1016/j.ridd.2013.09.021.

272. Nørgaard M, Lomholt J, Thastum M, Herlin M, Twilt M, Herlin T. Accelerometer-assessed daily physical activity in relation to pain cognition in juvenile idiopathic arthritis. Scand J Rheumatol. 2017 Jan;46(1):22–26. PMID: 27308924. doi: 10.3109/03009742.2016.1160146.

273. Nu UK, Touati T, Buddhadev S, Sun R, Smuck M, Song IHJ. Who is physically active? classification and analysis of physical activity using NHANES data. 2020 IEEE Symposium Series on Computational Intelligence (SSCI); 2021 Jan 05; Canberra, ACT, Australia. New York: IEEE; 2020. doi: 10.1109/SSCI47803.2020.9308353.

274. Ohkawara K, Oshima Y, Hikihara Y, Ishikawa-Takata K, Tabata I, Tanaka S. Real-time estimation of daily physical activity intensity by a triaxial accelerometer and a gravity-removal classification algorithm. Br J Nutr. 2011 Jun;105(11):1681–1691. PMID: 21262061. doi: 10.1017/S0007114510005441.

275. Oliver M, Schluter PJ, Schofield GM, Paterson J. Factors related to accelerometer-derived physical activity in pacific children aged 6 years. Asia Pac J Public Health. 2011 Jan;23(1):44–56. PMID: 20484244. doi: 10.1177/1010539510370992.

276. Orme MW, Steiner MC, Morgan MD, Kingsnorth AP, Esliger DW, Singh SJ, et al. 24-hour accelerometry in COPD: exploring physical activity, sedentary behavior, sleep and clinical characteristics. Int J Chron Obstruct Pulmon Dis. 2019 Feb;14:419–430. PMID: 30863042. doi: 10.2147/COPD.S183029.

277. Ortlieb S, Dias A, Gorzelniak L, Nowak D, Karrasch S, Peters A, et al. Exploring patterns of accelerometry-assessed physical activity in elderly people. Int J Behav Nutr Phys Act. 2014 Feb;11(1):28. PMID: 24575796. doi: 10.1186/1479-5868-11-28.

278. Ortlieb S, Gorzelniak L, Nowak D, Strobl R, Grill E, Thorand B, et al. Associations between multiple accelerometry-assessed physical activity parameters and selected health outcomes in elderly people – results from the KORA-age study. PLoS One. 2014 Nov;9(11):e111206. PMID: 25372399. doi: 10.1371/journal.pone.0111206.

279. Parada H, McDonald E, Bellettiere J, Evenson KR, LaMonte MJ, LaCroix AZ. Associations of accelerometer-measured physical activity and physical activity-related cancer incidence in older women: results from the WHI OPACH Study. Br J Cancer. 2020 Apr;122(9):1409–1416. PMID: 32139875. doi: 10.1038/s41416-020-0753-6.

280. Park J, Ishikawa-Takata K, Tanaka S, Mekata Y, Tabata I. Effects of walking speed and step frequency on estimation of physical activity Using accelerometers. J Physiol Anthropol. 2011;30(3):119–127. PMID: 21636955. doi: 10.2114/jpa2.30.119.

281. Parkka J, Ermes M, Antila K, Van Gils M, Manttari A, Nieminen H. Estimating intensity of physical activity: a comparison of wearable accelerometer and gyro sensors and 3 Sensor locations. 2007 29th Annual International Conference of the IEEE Engineering in Medicine and Biology Society; 2007 Oct 22; Lyon, France. New York: IEEE; 2007. PMID: 18002254. doi: 10.1109/IEMBS.2007.4352588.

282. Paschali AA, Kalantzi-Azizi A, Goodrick GK, Papadatou D, Balasubramanyam A. Accelerometer feedback to promote physical activity in adults with type 2 diabetes: a pilot study. Percept Mot Skills. 2005 Feb;100(1):61–68. PMID: 15773694. doi: 10.2466/pms.100.1.61-68.

283. Peter‐Marske KM, Evenson KR, Moore CC, Cuthbertson CC, Howard AG, Shiroma EJ, et al. Association of accelerometer‐measured physical activity and sedentary behavior with incident cardiovascular disease, myocardial infarction, and ischemic stroke: the women’s health study. J Am Heart Assoc. 2023 Apr;12(7):e028180. PMID: 36974744. doi: 10.1161/JAHA.122.028180.

284. Peters TM, Moore SC, Xiang YB, Yang G, Shu XO, Ekelund U, et al. Accelerometer-measured physical activity in Chinese adults. Am J Prev Med. 2010 Jun;38(6):583–591. PMID: 20494234. doi: 10.1016/j.amepre.2010.02.012.

285. Petersen TL, Brønd JC, Kristensen PL, Aadland E, Grøntved A, Jepsen R. Resemblance in accelerometer-assessed physical activity in families with children: the Lolland-Falster Health study. Int J Behav Nutr Phys Act. 2020 Dec;17(1):161. PMID: 33276796. doi: 10.1186/s12966-020-01067-7.

286. Pettee Gabriel K, Karvonen-Gutierrez CA, Colvin AB, Ylitalo KR, Whitaker KM, Lange-Maia BS, et al. Associations of accelerometer-determined sedentary behavior and physical activity with physical performance outcomes by race/ethnicity in older women. Prev Med Rep. 2021 May;23:101408. PMID: 34123715. doi: 10.1016/j.pmedr.2021.101408.

287. Pettee Gabriel K, Sternfeld B, Shiroma EJ, Pérez A, Cheung J, Lee IM. Bidirectional associations of accelerometer-determined sedentary behavior and physical activity with reported time in bed: Women’s Health Study. Sleep Health. 2017 Feb;3(1):49–55. PMID: 28346151. doi: 10.1016/j.sleh.2016.10.001.

288. Phan A, Askim T, Lydersen S, Indredavik B, Wethal T. Accelerometer-measured physical activity at 3 months as a predictor of symptoms of depression and anxiety 1 year after stroke: a multicentre prospective cohort study in central Norway. J Rehabil Med. 2023 Nov;55:jrm12309. PMID: 37970656. doi: 10.2340/jrm.v55.12309.

289. Plasqui G, Joosen AMCP, Kester AD, Goris AHC, Westerterp KR. Measuring free‐living energy expenditure and physical activity with triaxial accelerometry. Obes Res. 2005 Aug;13(8):1363–1369. PMID: 16129718. doi: 10.1038/oby.2005.165.

290. Pober DM, Staudenmayer J, Raphael C, Freedson PS. Development of novel techniques to classify physical activity mode using accelerometers. Med Sci Sports Exerc. 2006 Sep;38(9):1626–1634. PMID: 16960524. doi: 10.1249/01.mss.0000227542.43669.45.

291. Posis AIB, Bellettiere J, Salem RM, LaMonte MJ, Manson JE, Casanova R, et al. Associations of accelerometer-measured physical activity and sedentary time with all-cause mortality by genetic predisposition for longevity. J Aging Phys Act. 2022 Aug;31(2):265–275. PMID: 36002033. doi: 10.1123/japa.2022-0067.

292. Prioreschi A, Hodkinson B, Tikly M, McVeigh JA. Changes in physical activity measured by accelerometry following initiation of DMARD therapy in rheumatoid arthritis. Rheumatology. 2014 May;53(5):923–926. PMID: 24459221. doi: 10.1093/rheumatology/ket457.

293. Pugh ME, Buchowski MS, Robbins IM, Newman JH, Hemnes AR. Physical activity limitation as measured by accelerometry in pulmonary arterial hypertension. Chest. 2012 Dec;142(6):1391–1398. PMID: 22576635. doi: 10.1378/chest.12-0150.

294. Pulsford RM, Cortina-Borja M, Rich C, Kinnafick FE, Dezateux C, Griffiths LJ. Actigraph accelerometer-defined boundaries for sedentary behaviour and physical activity intensities in 7 year old children. PLoS One. 2011 Aug 11;6(8):e21822. PMID: 21853021. doi: 10.1371/journal.pone.0021822.

295. Qi G, Dutta D, Leroux A, Ray D, Muschelli J, Crainiceanu C, et al. Genome‐wide association studies of 27 accelerometry‐derived physical activity measurements identified novel loci and genetic mechanisms. Genet Epidemiol. 2022 Mar;46(2):122–138. PMID: 35043453. doi: 10.1002/gepi.22441.

296. Qi J, Liang HN, Chen J, Peng X, Newcombe L, Yang P. A hybrid hierarchical model for accessing physical activity recognition towards free-living environments. 2020 IEEE Intl Conf on Parallel & Distributed Processing with Applications, Big Data & Cloud Computing, Sustainable Computing & Communications, Social Computing & Networking (ISPA/BDCloud/SocialCom/SustainCom); 2020 Dec 17-19; Exeter, United Kingdom. New York: IEEE; 2020. doi: 10.1109/ISPA-BDCloud-SocialCom-SustainCom51426.2020.00199.

297. Qiu S, Xing Z. Association between accelerometer-derived physical activity and incident cardiac arrest. Europace. 2023 Dec;25(12):euad353. PMID: 38016070. doi: 10.1093/europace/euad353.

298. Rahman HA, Carrault G, Ge D, Amoud H, Prioux J, Le Faucheur A, et al. Ambulatory physical activity representation and classification using spectral distances approach. 2015 International Conference on Advances in Biomedical Engineering (ICABME); 2015 Nov 16-18; Beirut, Lebanon. New York: IEEE; 2015. doi: 10.1109/ICABME.2015.7323253.

299. Ramulu PY, Maul E, Hochberg C, Chan ES, Ferrucci L, Friedman DS. Real-world assessment of physical activity in glaucoma using an accelerometer. Ophthalmology. 2012 Jun;119(6):1159–1166. PMID: 22386950. doi: 10.1016/j.ophtha.2012.01.013.

300. Ricketts HC, Buchan DS, Steffensen F, Chaudhuri R, Baker JS, Cowan DC. Physical activity levels in asthma: relationship with disease severity, body mass index and novel accelerometer-derived metrics. J Asthma. 2023 Apr;60(4):824–834. PMID: 35876843. doi: 10.1080/02770903.2022.2102037.

301. Robertson MC, Green CE, Liao Y, Durand CP, Basen-Engquist KM. Self-efficacy and ohysical activity in overweight and obese adults participating in a worksite weight loss intervention: multistate modeling of wearable device data. Cancer Epidemiol Biomarkers Prev. 2020 Apr;29(4):769–776. PMID: 31871110. doi: 10.1158/1055-9965.EPI-19-0907.

302. Roscoe CMP, James RS, Duncan MJ. Calibration of GENEActiv accelerometer wrist cut-points for the assessment of physical activity intensity of preschool aged children. Eur J Pediatr. 2017 Aug;176(8):1093–1098. PMID: 28674825. doi: 10.1007/s00431-017-2948-2.

303. Roscoe CMP, James RS, Duncan MJ. Accelerometer-based physical activity levels differ between week and weekend days in british preschool children. J Funct Morphol Kinesiol. 2019 Sep;4(3):65. PMID: 33467380. doi: 10.3390/jfmk4030065.

304. Rowlands AV, Dempsey PC, Gillies C, Kloecker DE, Razieh C, Chudasama Y, et al. Association between accelerometer-assessed physical activity and severity of COVID-19 in UK Biobank. Mayo Clin Proc Innov Qual Outcomes. 2021 Dec;5(6):997–1007. PMID: 34430796. doi: 10.1016/j.mayocpiqo.2021.08.011.

305. Rowlands AV, Henson JJ, Coull NA, Edwardson CL, Brady E, Hall A, et al. The impact of COVID‐19 restrictions on accelerometer‐assessed physical activity and sleep in individuals with type 2 diabetes. Diabet Med. 2021 Oct;38(10):e14549. PMID: 33650112. doi: 10.1111/dme.14549.

306. Rowlands AV, Van Hees VT, Dawkins NP, Maylor BD, Plekhanova T, Henson J, et al. Accelerometer-assessed physical activity in people with type 2 diabetes: accounting for sleep when determining associations with markers of health. Sensors. 2023 Jun;23(12):5382. PMID: 37420551. doi: 10.3390/s23125382.

307. Rubin DS, Huisingh-Scheetz M, Hung A, Ward RP, Nagele P, Arena R, Hedeker D. Accuracy of physical function questions to predict moderate-vigorous physical activity as measured by hip accelerometry. Anesthesiology. 2019 Nov;131(5):992–1003. PMID: 31490293. doi: 10.1097/ALN.0000000000002911.

308. Salerno EA, Saint-Maurice PF, Wan F, Peterson LL, Park Y, Cao Y, et al. Prospective associations between accelerometry-derived physical activity and sedentary behaviors and mortality among cancer survivors. JNCI Cancer Spectr. 2023 Mar;7(2):pkad007. PMID: 36786414. doi: 10.1093/jncics/pkad007.

309. Salvo D, Torres C, Villa U, Rivera JA, Sarmiento OL, Reis RS, et al. Accelerometer-based physical activity levels among Mexican adults and their relation with sociodemographic characteristics and BMI: a cross-sectional study. Int J Behav Nutr Phys Act. 2015 Jun;12(1):79. PMID: 26088430. doi: 10.1186/s12966-015-0243-z,

310. Salway R, Foster C, De Vocht F, Tibbitts B, Emm-Collison L, House D, et al. Accelerometer-measured physical activity and sedentary time among children and their parents in the UK before and after COVID-19 lockdowns: a natural experiment. Int J Behav Nutr Phys Act. 2022 May;19(1):51. PMID: 35570265. doi: 10.1186/s12966-022-01290-4.

311. Sandroff BM, Motl RW, Kam JP, Pula JH. Accelerometer measured physical activity and the integrity of the anterior visual pathway in multiple sclerosis. Mult Scler Relat Disord. 2014 Jan;3(1):117–122. PMID: 25877982. doi: 10.1016/j.msard.2013.06.014.

312. Sasaki S, Nakamura K, Ukawa S, Okada E, Amagasa S, Inoue S, et al. Association of accelerometer-measured physical activity with kidney function in a Japanese population: the DOSANCO health study. BMC Nephrol. 2022 Jan;23(1):7. PMID: 34979979. doi: 10.1186/s12882-021-02635-0.

313. Savikangas T, Suominen TH, Alén M, Rantalainen T, Sipilä S. Changes in femoral neck bone mineral density and structural strength during a 12-month multicomponent exercise intervention among older adults – does accelerometer-measured physical activity matter? Bone. 2024 Jan;178:116951. PMID: 37913888. doi: 10.1016/j.bone.2023.116951.

314. Schumacher BT, Bellettiere J, LaMonte MJ, Evenson KR, Di C, Lee IM, et al. Accelerometer-measured daily steps, physical function, and subsequent fall risk in older women: the objective physical activity and cardiovascular disease in older women study. J Aging Phys Act. 2021 Oct;30(4):635–645. PMID: 34627127. doi: 10.1123/japa.2021-0159.

315. Schwendinger F, Wagner J, Infanger D, Schmidt-Trucksäss A, Knaier R. Methodological aspects for accelerometer-based assessment of physical activity in heart failure and health. BMC Med Res Methodol. 2021 Nov;21(1):251. PMID: 34775952. doi: 10.1186/s12874-021-01350-6.

316. Scott D, Johansson J, Gandham A, Ebeling PR, Nordstrom P, Nordstrom A. Associations of accelerometer-determined physical activity and sedentary behavior with sarcopenia and incident falls over 12 months in community-dwelling Swedish older adults. J Sport Health Sci. 2021 Sep;10(5):577–584. PMID: 34088651. doi: 10.1016/j.jshs.2020.01.006.

317. Seguin-Fowler RA, LaCroix AZ, LaMonte MJ, Liu J, Maddock JE, Rethorst CD, et al. Association of neighborhood Walk Score with accelerometer-measured physical activity varies by neighborhood socioeconomic status in older women. Prev Med Rep. 2022 Jul;29:101931. PMID: 36161128. doi: 10.1016/j.pmedr.2022.101931.

318. Serra MC, Balraj E, DiSanzo BL, Ivey FM, Hafer-Macko CE, Treuth MS, et al. Validating accelerometry as a measure of physical activity and energy expenditure in chronic stroke. Top Stroke Rehabil. 2017 Jan 2;24(1):18–23. PMID: 27322733. doi: 10.1080/10749357.2016.1183866.

319. Sevil M, Rashid M, Maloney Z, Hajizadeh I, Samadi S, Askari MR, et al. Determining physical activity characteristics from wristband data for use in automated insulin delivery systems. IEEE Sens J. 2020 Nov;20(21):12859–12870. doi: 10.1109/JSEN.2020.3000772.

320. Shadyab AH, LaMonte MJ, Kooperberg C, Reiner AP, Carty CL, Manini TM, et al. Association of accelerometer-measured physical activity with leukocyte telomere length among older women. J Gerontol A Biol Sci Med Sci. 2017 Oct;72(11):1532–1537. PMID: 28329327. doi: 10.1093/gerona/glx037.

321. Sharma B, Obeid J, DeMatteo C, Noseworthy MD, Timmons BW. New insights into accelerometer-measured habitual physical activity and sedentary time during early recovery in pediatric concussion. Pediatric Exerc Sci. 2023 Aug;1–8. PMID: 37591503. doi: 10.1123/pes.2023-0016.

322. Shim J, Fleisch E, Barata F. Precise segmentation of U.S. adults from 24-hour wearable-based physical activity profiles using machine learning clustering. 2023 IEEE 11th International Conference on Healthcare Informatics (ICHI) Houston. 2023 Jun 26-29; TX, USA. New York: IEEE; 2023. doi: 10.1109/ICHI57859.2023.00083.

323. Shin SW, Kim H, Kang YH, Kim J. Accelerometer-derived physical activity and sedentary behavior patterns among Korean adults. Phys Act Nutr. 2023 Jun 30;27(2):025–033. PMID: 37583069. doi: 10.20463/pan.2023.0015.

324. Shin YE, Choi WH, Shin TM. Physical activity recognition based on rotated acceleration data using quaternion in sedentary behavior : a preliminary study. 2014 36th Annual International Conference of the IEEE Engineering in Medicine and Biology Society; 2014 Aug 26-30; Chicago, US. New York: IEEE; 2014. doi: 10.1109/EMBC.2014.6944741

325. Shwetar Y, Huang Z, Veerubhotla A, Knezevic S, Hong E, Spungen AM, et al. Predicting physical activity intensity using raw accelerometer signals in manual wheelchair users with spinal cord injury. Spinal Cord. 2022 Feb;60(2):149–156. PMID: 34819608. doi: 10.1038/s41393-021-00728-z.

326. Si K, Cao Z, Liu Q, Yang Y, Dai Q, Yao Y, et al. Accelerometer-measured physical activity, sedentary behavior, and risk of incident pelvic organ prolapse: a prospective cohort study in the UK Biobank. Int J Behav Nutr Phys Act. 2024 Feb;21(1):12. PMID: 38308373. doi: 10.1186/s12966-024-01559-w.

327. Siddique J, Aaby D, Montag SE, Sidney S, Sternfeld B, Welch WA, et al. Individualized relative-intensity physical activity accelerometer cut points. Med Sci Sports Exerc. 2020 Feb;52(2):398–407. PMID: 31524826. doi: 10.1249/MSS.0000000000002153.

328. Silva RDM, Cabral LLP, Browne RAV, Lemos TMAM, Alves CPDL, Crochemore-Silva I, et al. Joint associations of accelerometer-measured physical activity and sedentary time with cardiometabolic risk in older adults: A cross-sectional study. Exp Gerontol. 2022 Aug;165:111839. PMID: 35609715. doi: 10.1016/j.exger.2022.111839.

329. Simón-Vicente L, Rivadeneyra-Posadas J, Soto-Célix M, Raya-González J, Castillo D, Calvo S, et al. Accelerometer cut-points for physical activity assessment in adults with mild to moderate Huntington’s disease: a cross-sectional multicentre study. Int J Environ Res Public Health. 2022 Nov;19(22):14834. PMID: 36429552. doi: 10.3390/ijerph192214834.

330. Sit CHP, Huang WY, Yu JJ, McKenzie TL. Accelerometer-assessed physical activity and sedentary time at school for children with disabilities: seasonal variation. Int J Environ Res Public Health. 2019 Aug;16(17):3163. PMID: 31480233. doi: 10.3390/ijerph16173163.

331. Sjöros T, Vähä-Ypyä H, Laine S, Garthwaite T, Lahesmaa M, Laurila SM, et al. Both sedentary time and physical activity are associated with cardiometabolic health in overweight adults in a 1 month accelerometer measurement. Sci Rep. 2020 Nov;10(1):20578. PMID: 33239818. doi: 10.1038/s41598-020-77637-3.

332. Slootmaker SM, Chinapaw MJM, Seidell JC, Van Mechelen W, Schuit AJ. Accelerometers and internet for physical activity promotion in youth? Feasibility and effectiveness of a minimal intervention [ISRCTN93896459]. Prev Med. 2010 Jul;51(1):31–36. PMID: 20380847. doi: 10.1016/j.ypmed.2010.03.015.

333. Smirnova E, Leroux A, Cao Q, Tabacu L, Zipunnikov V, Crainiceanu C, Urbanek JK. The predictive performance of objective measures of physical activity derived from accelerometry data for 5-year all-cause mortality in older adults: national health and nutritional examination survey 2003–2006. J Gerontol A Biol Sci Med Sci. 2020 Sep;75(9):1779–1785. PMID: 31504213. doi: 10.1093/gerona/glz193.

334. Smith DM, DeCaro JA, Murphy SL, Parmelee PA. Momentary reports of fatigue predict physical activity level: wrist, waist, and combined accelerometry. J Aging Health. 2020 Oct;32(9):921–925. PMID: 31319748. doi: 10.1177/0898264319863609.

335. Smith HA, Storti KL, Arena VC, Kriska AM, Gabriel KKP, Sutton-Tyrrell K, et al. Associations between accelerometer-derived physical activity and regional adiposity in young men and women: Accelerometer and Regional Adiposity. Obesity. 2013 Jun;21(6):1299–1305. PMID: 23408709. doi: 10.1002/oby.20308.

336. Smith ID, Ross LM, Gabaldon JR, Holdgate N, Pieper CF, Ning TC, et al. The relation of accelerometer-measured physical activity and serum uric acid using the national health and nutrition survey (NHANES) 2003–2004. Front Sports Act Living. 2022 Jan;3:775398. PMID: 35098119. doi: 10.3389/fspor.2021.775398.

337. Smith KE, O’Connor SM, Mason TB, Wang S, Dzubur E, Crosby RD, et al. Associations between objective physical activity and emotional eating among adiposity‐discordant siblings using ecological momentary assessment and accelerometers. Pediatr Obes. 2021 Mar;16(3):e12720. PMID: 32881329. doi: 10.1111/ijpo.12720.

338. Spartano NL, Davis-Plourde KL, Himali JJ, Andersson C, Pase MP, Maillard P, et al. Association of accelerometer-measured light-intensity physical activity with brain volume: the Framingham heart study. JAMA Netw Open. 2019 Apr;2(4):e192745. PMID: 31002329. doi: 10.1001/jamanetworkopen.2019.2745.

339. Spartano NL, Demissie S, Himali JJ, Dukes KA, Murabito JM, Vasan RS, et al. Accelerometer‐determined physical activity and cognitive function in middle‐aged and older adults from two generations of the Framingham Heart Study. Alzheimers Dement. 2019 Oct;5(1):618–626. PMID: 31660424. doi: 10.1016/j.trci.2019.08.007.

340. Spartano NL, Heffernan KS, Dumas AK, Gump BB. Accelerometer-determined physical activity and the cardiovascular response to mental stress in children. J Sci Med Sport. 2017 Jan;20(1):60–65. PMID: 27283342. doi: 10.1016/j.jsams.2016.05.008.

341. Spartano NL, Wang R, Yang Q, Chernofsky A, Murabito JM, Vasan RS, et al. Association of accelerometer-measured physical activity and sedentary time with epigenetic markers of aging. Med Sci Sports Exerc. 2023 Feb;55(2):264–272. PMID: 36107108. doi: 10.1249/MSS.0000000000003041.

342. Stamatakis E, Ahmadi MN, Friedenreich CM, Blodgett JM, Koster A, Holtermann A, et al. Vigorous intermittent lifestyle physical activity and cancer incidence among nonexercising adults: the UK Biobank accelerometry study. JAMA Oncol. 2023 Sep;9(9):1255–1259. PMID: 37498576. doi: 10.1001/jamaoncol.2023.1830.

343. Stamatakis E, Ahmadi MN, Gill JMR, Thøgersen-Ntoumani C, Gibala MJ, Doherty A, et al. Association of wearable device-measured vigorous intermittent lifestyle physical activity with mortality. Nat Med. 2022 Dec;28(12):2521–2529. PMID: 36482104. doi: 10.1038/s41591-022-02100-x.

344. Stevens ML, Crowley P, Rasmussen CL, Hallman DM, Mortensen OS, Nygård CH, et al. Accelerometer-measured physical activity at work and need for recovery: a compositional analysis of cross-sectional data. Ann Work Expo Health. 2020 Feb;64(2):138–151. PMID: 31879769. doi: 10.1093/annweh/wxz095.

345. Stewart T, Narayanan A, Hedayatrad L, Neville J, Mackay L, Duncan S. A dual-accelerometer system for classifying physical activity in children and adults. Med Sci Sports Exerc. 2018 Dec;50(12):2595–2602. PMID: 30048411. doi: 10.1249/MSS.0000000000001717.

346. Straight CR, Ward-Ritacco CL, Evans EM. Association between accelerometer-measured physical activity and muscle capacity in middle-aged postmenopausal women. Menopause. 2015 Nov;22(11):1204–1211. PMID: 25783471. doi: 10.1097/GME.0000000000000447.

347. Strain T, Wijndaele K, Dempsey PC, Sharp SJ, Pearce M, Jeon J, et al. Wearable-device-measured physical activity and future health risk. Nat Med. 2020 Sep;26(9):1385–1391. PMID: 32807930. doi: 10.1038/s41591-020-1012-3.

348. Stubbs B, Chen LJ, Chung MS, Ku PW. Physical activity ameliorates the association between sedentary behavior and cardiometabolic risk among inpatients with schizophrenia: A comparison versus controls using accelerometry. Compr Psychiatry. 2017 Apr;74:144–150. PMID: 28167327. doi: 10.1016/j.comppsych.2017.01.010.

349. Sufrinko AM, Howie EK, Elbin RJ, Collins MW, Kontos AP. A preliminary investigation of accelerometer-derived sleep and physical activity following sport-related concussion. J Head Trauma Rehabil. 2018 Sep;33(5):E64–E74. PMID: 29601343. doi: 10.1097/HTR.0000000000000387.

350. Sutin AR, Luchetti M, Stephan Y, Terracciano A. Meaning in life and accelerometer-measured physical activity: association based on 67,038 UK Biobank participants. Ment Health Phys Act. 2021 Oct;21:100412. PMID: 35340340. doi: 10.1016/j.mhpa.2021.100412.

351. Sutin AR, Stephan Y, Kekäläinen T, Luchetti M, Terracciano A. Purpose in life and accelerometer-measured physical activity among older adults. Psychol Health. 2023 Apr;1–15. PMID:37073429. doi: 10.1080/08870446.2023.2200414.

352. Sweegers MG, Buffart LM, Huijsmans RJ, Konings IR, Van Zweeden AA, Brug J, et al. From accelerometer output to physical activity intensities in breast cancer patients. J Sci Med Sport. 2020 Feb;23(2):176–181. PMID: 31537492. doi: 10.1016/j.jsams.2019.09.001.

353. Tabacu L, Ledbetter M, Leroux A, Crainiceanu C, Smirnova E. Quantifying the varying predictive value of physical activity measures obtained from wearable accelerometers on all-cause mortality over short to medium time horizons in NHANES 2003–2006. Sensors. 2020 Dec;21(1):4. PMID: 33374911. doi: 10.3390/s21010004.

354. Takae R, Hatamoto Y, Yasukata J, Kose Y, Komiyama T, Ikenaga M, et al. Association of lower-extremity muscle performance and physical activity level and intensity in middle-aged and older adults: a doubly labeled water and accelerometer study. J Nutr Health Aging. 2020 Sep;24(9):1023–1030. PMID: 33155632. doi: 10.1007/s12603-020-1514-1.

355. Tanaka C, Hikihara Y, Ando T, Oshima Y, Usui C, Ohgi Y, et al. Prediction of physical activity intensity with accelerometry in young children. Int J Environ Res Public Health. 2019 Mar;16(6):931. PMID: 30875871. doi: 10.3390/ijerph16060931.

356. Tanaka C, Shikano A, Imai N, Chong KH, Howard SJ, Tanabe K, et al. Accelerometer-measured physical activity and sedentary time among children in Japan before and during COVID-19: a cross-sectional and longitudinal analysis. Int J Environ Res Public Health. 2023 Jan;20(2):1130. PMID: 36673886. doi: 10.3390/ijerph20021130.

357. Tanaka C, Tanaka S. Daily phsysical activity in Japanese preschool children evaluated by triaxial accelerometry: the relationship between period of engagement in moderate-to-vigorous physical activity and daily step counts. J Physiol Anthropol. 2009 Nov;28(6):283–288. PMID: 20009376. doi: 10.2114/jpa2.28.283.

358. Tanaka C, Tanaka S, Kawahara J, Midorikawa T. Triaxial accelerometry for assessment of physical activity in young children. Obes. 2007 May;15(5):1233–1241. PMID: 17495200. doi: 10.1038/oby.2007.145.

359. Tanaka S, Ishikawa-Takata K, Nakae S, Sasaki S. Prediction of the physical activity level of community-dwelling older Japanese adults with a triaxial accelerometer containing a classification algorithm for ambulatory and non-ambulatory activities. Sensors. 2023 May;23(10):4960. PMID: 37430874. doi: 10.3390/s23104960.

360. Tao K, Liu W, Xiong S, Ken L, Zeng N, Peng Q, et al. Associations between self-determined motivation, accelerometer-determined physical activity, and quality of Life in Chinese college students. Int J Environ Res Public Health. 2019 Aug;16(16):2941. PMID: 31426280. doi: 10.3390/ijerph16162941.

361. Tarp J, Bugge A, Andersen LB, Sardinha LB, Ekelund U, Brage S, et al. Does adiposity mediate the relationship between physical activity and biological risk factors in youth? – A cross-sectional study from the international children’s accelerometry database (ICAD). Int J Obes. 2017 Oct;10.1038/ijo.2017.241. PMID:29087387.

362. Tarp J, Hansen BH, Fagerland MW, Steene-Johannessen J, Anderssen SA, Ekelund U. Accelerometer-measured physical activity and sedentary time in a cohort of US adults followed for up to 13 years: the influence of removing early follow-up on associations with mortality. Int J Behav Nutr Phys Act. 2020 Mar;17(1):39. PMID: 32169059. doi: 10.1186/s12966-020-00945-4.

363. Tasheva P, Kraege V, Vollenweider P, Roulet G, Méan M, Marques-Vidal P. Accelerometry assessed physical activity of older adults hospitalized with acute medical illness - an observational study. BMC Geriatr. 2020 Oct;20(1):382. PMID: 33008378. doi: 10.1186/s12877-020-01763-w.

364. Taylor RW, Williams SM, Farmer VL, Taylor BJ. Changes in physical activity over time in young children: a longitudinal study using accelerometers. PLoS One. 2013 Nov;8(11):e81567. PMID: 24282607. doi: 10.1371/journal.pone.0081567.

365. Thornton CB, Kolehmainen N, Nazarpour K. Using unsupervised machine learning to quantify physical activity from accelerometry in a diverse and rapidly changing population. PLOS Digit Health. 2023 Apr;2(4):e0000220. PMID: 37018183. doi: 10.1371/journal.pdig.0000220.

366. Thraen-Borowski KM, Gennuso KP, Cadmus-Bertram L. Accelerometer-derived physical activity and sedentary time by cancer type in the United States. PLoS One. 2017 Aug;12(8):e0182554. PMID: 28806753. doi: 10.1371/journal.pone.0182554.

367. Thralls KJ, Godbole S, Manini TM, Johnson E, Natarajan L, Kerr J. A comparison of accelerometry analysis methods for physical activity in older adult women and associations with health outcomes over time. J Sports Sci. 2019 Oct;37(20):2309–2317. PMID: 31195893. doi: 10.1080/02640414.2019.1631080.

368. Toivo K, Vähä‐Ypyä H, Kannus P, Tokola K, Alanko L, Heinonen OJ, et al. Physical activity measured by accelerometry among adolescents participating in sports clubs and non‐participating peers. Eur J Sport Sci. 2023 Jul;23(7):1426–1434. PMID: 35861140. doi: 10.1080/17461391.2022.2103740.

369. Toomey CM, Whittaker JL, Doyle-Baker PK, Emery CA. Does a history of youth sport-related knee injury still impact accelerometer-measured levels of physical activity after 3–12 years? Phys Ther Sport. 2022 May;55:90–97. PMID: 35290947. doi: 10.1016/j.ptsp.2022.03.003.

370. Trinh L, Motl RW, Roberts SA, Gibbons T, McAuley E. Estimation of physical activity intensity cut‐points using accelerometry in breast cancer survivors and age‐matched controls. Eur J Cancer Care. 2019 Sep;28(5). PMID: 31106924. doi: 10.1111/ecc.13090.

371. Trost SG, Brookes DSK, Ahmadi MN. Evaluation of wrist accelerometer cut-points for classifying physical activity intensity in youth. Front Digit Health. 2022 May;4:884307. PMID: 35585912. doi: 10.3389/fdgth.2022.884307.

372. Trumpeter NN, Lawman HG, Wilson DK, Pate RR, Van Horn M, Tate AK. Accelerometry cut points for physical activity in underserved African Americans. Int J Behav Nutr Phys Act. 2012;9(1):73. PMID: 22697280. doi: 10.1186/1479-5868-9-73.

373. Tsai L, Boyle E, Buhl SF, Kock G, Brønd JC, Visser M, et al. Associations between appetite, physical activity and sedentary behaviour from hip‐ and wrist‐worn accelerometers in community‐dwelling older adults. Geriatrics Gerontol Int. 2023 Jun;23(6):411–417. PMID: 37098733. doi: 10.1111/ggi.14588.

374. Tsunoda K, Kitano N, Kai Y, Jindo T, Uchida K, Arao T. Dose–response relationships of accelerometer‐measured sedentary behaviour and physical activity with non‐alcoholic fatty liver disease. Aliment Pharmacol Ther. 2021 Nov;54(10):1330–1339. PMID: 34633105. doi: 10.1111/apt.16631.

375. Vaara JP, Kyröläinen H, Vasankari T, Kainulainen H, Raitanen J, Kujala UM. Accelerometer-based sedentary time, physical activity, and serum metabolome in young men. Metabolites. 2022 Jul;12(8):700. PMID: 36005572. doi: 10.3390/metabo12080700.

376. Vähä-Ypyä H, Vasankari T, Husu P, Mänttäri A, Vuorimaa T, Suni J, et al. Validation of cut-points for evaluating the intensity of physical activity with accelerometry-based mean amplitude deviation (MAD). PLoS One. 2015 Aug;10(8):e0134813. PMID: 26292225. doi: 10.1371/journal.pone.0134813.

377. Valenti G, Camps SGJA, Verhoef SPM, Bonomi AG, Westerterp KR. Validating measures of free-living physical activity in overweight and obese subjects using an accelerometer. Int J Obes. 2014 Jul;38(7):1011–1014. PMID: 24166066. doi: 10.1038/ijo.2013.195.

378. Van Bakergem M, Sommer EC, Heerman WJ, Hipp JA, Barkin SL. Objective reports versus subjective perceptions of crime and their relationships to accelerometer-measured physical activity in Hispanic caretaker-child dyads. Prev Med. 2017 Feb;95:S68–S74. PMID: 27939263. doi: 10.1016/j.ypmed.2016.12.001.

379. Van Ballegooijen AJ, Van Der Ploeg HP, Visser M. Daily sedentary time and physical activity as assessed by accelerometry and their correlates in older adults. Eur Rev Aging Phys Act. 2019 Feb;16(1):3. PMID: 30820261. doi: 10.1186/s11556-019-0210-9.

380. Van Der Zee-Neuen A, Wirth W, Hösl K, Osterbrink J, Eckstein F. The association of physical activity and depression in patients with, or at risk of, osteoarthritis is captured equally well by patient reported outcomes (PROs) and accelerometer measurements - Analyses of data from the Osteoarthritis Initiative. Semin Arthritis Rheum. 2019 Dec;49(3):325–330. PMID: 31248586. doi: 10.1016/j.semarthrit.2019.05.009.

381. Van Dyck D, Barnett A, Cerin E, Conway TL, Esteban-Cornejo I, Hinckson E, et al. Associations of accelerometer measured school- and non-school based physical activity and sedentary time with body mass index: IPEN adolescent study. Int J Behav Nutr Phys Act. 2022 Jul;19(1):85. PMID: 35836235. doi: 10.1186/s12966-022-01324-x.

382. Van Dyck D, Cerin E, De Bourdeaudhuij I, Salvo D, Christiansen LB, Macfarlane D, et al. Moderating effects of age, gender and education on the associations of perceived neighborhood environment attributes with accelerometer-based physical activity: the IPEN adult study. Health Place. 2015 Nov;36:65–73. PMID: 26454247. doi: 10.1016/j.healthplace.2015.09.007.

383. Van Eijk RPA, Bakers JNE, Bunte TM, De Fockert AJ, Eijkemans MJC, Van Den Berg LH. Accelerometry for remote monitoring of physical activity in amyotrophic lateral sclerosis: a longitudinal cohort study. J Neurol. 2019 Oct;266(10):2387–2395. PMID: 31187191. doi: 10.1007/s00415-019-09427-5.

384. Van Genderen S, Boonen A, Van Der Heijde D, Heuft L, Luime J, Spoorenberg A, et al. Accelerometer quantification of physical activity and activity patterns in patients with ankylosing spondylitis and population controls. J Rheumatol. 2015 Dec;42(12):2369–2375. PMID: 26523021. doi: 10.3899/jrheum.150015.

385. Van Landingham SW, Willis JR, Vitale S, Ramulu PY. Visual field loss and accelerometer-measured physical activity in the United States. Ophthalmology. 2012 Dec;119(12):2486–2492. PMID: 22892152. doi: 10.1016/j.ophtha.2012.06.034.

386. Van Langenberg DR, Papandony MC, Gibson PR. Sleep and physical activity measured by accelerometry in Crohn’s disease. Aliment Pharmacol Ther. 2015 May;41(10):991–1004. PMID: 25783784. doi: 10.1111/apt.13160.

387. Vandercappellen EJ, Koster A, Savelberg HHCM, Eussen SJPM, Dagnelie PC, Schram MT, et al. Accelerometer-derived physical activity and sedentary time and cardiac biomarkers: the Maastricht study. Front Cardiovasc Med. 2023 Apr 28;10:1081713. PMID: 37187790. doi: 10.3389/fcvm.2023.1081713.

388. Vanhelst J, Béghin L, Turck D, Gottrand F. New validated thresholds for various intensities of physical activity in adolescents using the Actigraph accelerometer. Int J Rehabil Res. 2011 Jun;34(2):175–177. PMID: 20890210. doi: 10.1097/MRR.0b013e328340129e.

389. Vanhelst J, Hurdiel R, Mikulovic J, Bui-Xuân G, Fardy P, Theunynck D, et al. Validation of the Vivago wrist-worn accelerometer in the assessment of physical activity. BMC Public Health. 2012 Aug;12(1):690. PMID: 22913286. doi: 10.1186/1471-2458-12-690.

390. Vásquez E, Strizich G, Isasi CR, Echeverria SE, Sotres-Alvarez D, Evenson KR, et al. Is there a relationship between accelerometer-assessed physical activity and sedentary behavior and cognitive function in US Hispanic/Latino adults? the Hispanic community health study/study of Latinos (HCHS/SOL). Prev Med. 2017 Oct;103:43–48. PMID: 28765082. doi: 10.1016/j.ypmed.2017.07.024.

391. Vásquez PM, Tarraf W, Chai A, Doza A, Sotres-Alvarez D, Diaz KM, et al. Accelerometer-measured latent physical activity profiles and neurocognition among middle-aged and older Hispanic/Latino adults in the Hispanic community health study/study of Latinos (HCHS/SOL). J Gerontol B Psychol Sci Soc Sci. 2022 Dec;77(12):e263–e278. PMID: 36219450. doi: 10.1093/geronb/gbac161.

392. Vásquez PM, Durazo-Arvizu RA, Marquez DX, Argos M, Lamar M, Odoms-Young A, et al. Association of accelerometer-measured physical activity and cardiovascular health in the Hispanic community health study/study of Latinos (HCHS/SOL). Hisp Health Care Int. 2022 Mar;20(1):15–24. PMID: 33685281. doi: 10.1177/1540415320985581.

393. Verbestel V, De Henauw S, Bammann K, Barba G, Hadjigeorgiou C, Eiben G, et al. Are context-specific measures of parental-reported physical activity and sedentary behaviour associated with accelerometer data in 2–9-year-old European children? Public Health Nutr. 2015 Apr;18(5):860–868. PMID: 24887315. doi: 10.1017/S136898001400086X.

394. Verma VK, Lin WY. Machine learning-based 30-day hospital readmission predictions for COPD patients using physical activity data of daily living with accelerometer-based device. Biosensors. 2022 Aug;12(8):605. PMID: 36005000. doi: 10.3390/bios12080605.

395. Vorwerg Y, Petroff D, Kiess W, Blüher S. Physical activity in 3–6 year old children measured by SenseWear Pro®: direct accelerometry in the course of the week and relation to weight status, media consumption, and socioeconomic factors. PLoS One. 2013 Apr;8(4):e60619. PMID: 23573273. doi: 10.1371/journal.pone.0060619.

396. Wang Y, Wattelez G, Frayon S, Caillaud C, Galy O, Yacef K. ABIPA: ARIMA-based integration of accelerometer-based physical activity for adolescent weight status prediction. ACM Trans Comput Healthcare. 2023 Jan;4(1):1–19. doi: 10.1145/3561611.

397. Watts EL, Saint-Maurice PF, Doherty A, Fensom GK, Freeman JR, Gorzelitz JS, et al. Association of accelerometer-measured physical activity level with risks of hospitalization for 25 common health conditions in UK adults. JAMA Netw Open. 2023 Feb;6(2):e2256186. PMID: 36795414. doi: 10.1001/jamanetworkopen.2022.56186.

398. Weaver RG, Crimarco A, Brusseau TA, Webster CA, Burns RD, Hannon JC. Accelerometry‐derived physical activity of first through third grade children during the segmented school day. J Sch Health. 2016 Oct;86(10):726–733. PMID: 27619763. doi: 10.1111/josh.12426.

399. Wendt A, Da Silva ICM, Gonçalves H, Menezes A, Barros F, Wehrmeister FC. Short-term effect of physical activity on sleep health: a population-based study using accelerometry. J Sport Health Sci. 2022 Sep;11(5):630–638. PMID: 32422346. doi: 10.1016/j.jshs.2020.04.007.

400. Werneck AO, Jago R, Kriemler S, Andersen LB, Wedderkopp N, Northstone K, et al. Association of change in the school travel mode with changes in different physical activity intensities and sedentary time: a international children’s accelerometry database study. Prev Med. 2021 Dec;153:106862. PMID: 34710443. doi: 10.1016/j.ypmed.2021.106862.

401. Whitaker KM, Pettee Gabriel K, Buman MP, Pereira MA, Jacobs DR, Reis JP, et al. Associations of accelerometer‐measured sedentary time and physical activity with prospectively assessed cardiometabolic risk factors: the CARDIA study. J Am Heart Assoc. 2019 Jan;8(1):e010212. PMID: 30616480. doi: 10.1161/JAHA.118.010212.

402. Whitaker KM, Pettee Gabriel K, Laddu D, White DK, Sidney S, Sternfeld B, et al. Bidirectional associations of accelerometer measured sedentary behavior and physical activity with knee pain, stiffness, and physical function: the CARDIA study. Prev Med Rep. 2021 Mar;22:101348. PMID: 33816086. doi: 10.1016/j.pmedr.2021.101348

403. Whitaker KM, Xiao Q, Pettee Gabriel K, Gordon Larsen P, Jacobs DR, Sidney S, et al. Perceived and objective characteristics of the neighborhood environment are associated with accelerometer-measured sedentary time and physical activity, the CARDIA study. Prev Med. 2019 Jun;123:242–249. PMID: 30940573. doi: 10.1016/j.ypmed.2019.03.039.

404. Whitaker KM, Zhang D, Pettee Gabriel K, Ahrens M, Sternfeld B, Sidney S, et al. Longitudinal associations of midlife accelerometer determined sedentary behavior and physical activity with cognitive function: the CARDIA study. J Am Heart Assoc. 2021 Feb;10(3):e018350. PMID: 33470140. doi: 10.1161/JAHA.120.018350.

405. White T, Westgate K, Wareham NJ, Brage S. Estimation of physical activity energy expenditure during free-living from wrist accelerometry in UK adults. PLoS One. 2016 Dec 9;11(12):e0167472. PMID: 27936024. doi: 10.1371/journal.pone.0167472.

406. Willis JR, Jefferys JL, Vitale S, Ramulu PY. Visual impairment, uncorrected refractive error, and accelerometer-defined physical activity in the United States. Arch Ophthalmol. 2012 Mar;130(3):329-35. PMID: 22411662. doi: 10.1001/archopthalmol.2011.1773.

407. Wu J, Olson JL, Brunke-Reese D, Lagoa CM, Conroy DE. Wearable device adherence among insufficiently-active young adults is independent of identity and motivation for physical activity. J Behav Med. 2024 Apr;47(2):197–206. PMID: 37642938. doi: 10.1007/s10865-023-00444-4.

408. Wu Y, Goodrich JM, Dolinoy DC, Sánchez BN, Ruiz-Narváez EA, Banker M, et al. Accelerometer-measured physical activity, reproductive hormones, and DNA methylation. Med Sci Sports Exerc. 2020 Mar;52(3):598–607. PMID: 31652236. doi: 10.1249/MSS.0000000000002175.

409. Wu Z, Wang Z, Hu B, Zhang X, Zhang F, Wang H, et al. Relationships of accelerometer-based measured objective physical activity and sedentary behaviour with cognitive function: a comparative cross-sectional study of China’s elderly population. BMC Geriatr. 2020 Apr;20(1):149. PMID: 32321436. doi: 10.1186/s12877-020-01521-y.

410. Wullems JA, Verschueren SMP, Degens H, Morse CI, Onambélé GL. Performance of thigh-mounted triaxial accelerometer algorithms in objective quantification of sedentary behaviour and physical activity in older adults. PLoS One. 2017 Nov;12(11):e0188215. PMID: 29155839. doi: 10.1371/journal.pone.0188215.

411. Xie C, Bi S, Dong M, Li L, Chi S. Walking recognition method for physical activity analysis system of child based on wearable accelerometer. 2017 IEEE International Conference on Robotics and Biomimetics (ROBIO); 2017 Dec 05-08; Macau, Macao. New York: IEEE; 2017. doi: 10.1109/ROBIO.2017.8324785.

412. Xiuxin Yang, Anh Dinh, Li Chen. Implementation of a wearerable real-time system for physical activity recognition based on Naive Bayes classifier. 2010 International Conference on Bioinformatics and Biomedical Technology; 2010 Apr 16-18; Chengdu, China. New York: IEEE; 2010. doi: 10.1109/ICBBT.2010.5479000.

413. Yamaga Y, Svensson T, Chung U, Svensson AK. Association between metabolic syndrome status and daily physical activity measured by a wearable device in Japanese office workers. Int J Environ Res Public Health. 2023 Feb;20(5):4315. PMID: 36901325. doi: 10.3390/ijerph20054315.

414. Yang CH, Wang S, Wang WL, Belcher BR, Dunton GF. Day-level associations of physical activity and sedentary time in mother–child dyads across three years: a multi-wave longitudinal study using accelerometers. J Behav Med. 2022 Oct;45(5):702–715. PMID: 35753007. doi: 10.1007/s10865-022-00335-0.

415. Yang W, Li MH, Yu JJ, Wong SH, Sum RK-W, Sit CH. The associations between accelerometer-measured physical activity levels and mental health in children and adolescents with intellectual disabilities during the COVID-19 pandemic. J Autism Dev Disord. 2023 Dec;53(12):4809–4821. PMID: 36181649. doi: 10.1007/s10803-022-05777-8.

416. Yen HY, Liao Y, Huang HY. Smart wearable device users’ behavior is essential for physical activity improvement. Int J Behav Med. 2022 Jun;29(3):278–285. PMID: 34363130. doi: 10.1007/s12529-021-10013-1.

417. Yerrakalva D, Hajna S, Wijndaele K, Dempsey PC, Westgate K, Wareham N, et al. Bidirectional associations of accelerometer-assessed physical activity and sedentary time with physical function among older English adults: the EPIC-Norfolk cohort study. Eur J Ageing. 2022 Oct;19(4):1507–1517. PMID: 36506675. doi: 10.1007/s10433-022-00733-y.

418. Yi L, Mason TB, Yang CH, Chu D, Dunton GF. Longitudinal associations between neighborhood park and open space access and children’s accelerometer-assessed Measured physical activity: the evidence from the MATCH study. J Phy Act Health. 2021 Jul;18(9):1058–1066. PMID: 34198261. doi: 10.1123/jpah.2021-0177.

419. Yoshioka M, Ayabe M, Yahiro T, Higuchi H, Higaki Y, St-Amand J, et al. Long-period accelerometer monitoring shows the role of physical activity in overweight and obesity. Int J Obes. 2005 May;29(5):502–508. PMID: 15672105. doi: 10.1038/sj.ijo.0802891.

420. You Y, Chen Y, Wang X, Wei M, Zhang Q, Cao Q. Accelerometer-measured physical activity patterns are associated with phenotypic age: isotemporal substitution effects. Heliyon. 2023 Aug;9(9):e19158. PMID: 37810111. doi: 10.1016/j.heliyon.2023.e19158.

421. Yu X, Hao L, Crainiceanu C, Leroux A. Occupational determinants of physical activity at work: evidence from wearable accelerometer in 2005–2006 NHANES. SSM Popul Health. 2021 Dec;17:100989. PMID: 34977325. doi: 10.1016/j.ssmph.2021.100989.

422. Zhang S, Rowlands AV, Murray P, Hurst TL. Physical activity classification using the GENEA wrist-worn accelerometer. Med Sci Sports Exerc. 2012 Apr;44(4):742–748. PMID: 21988935. doi: 10.1249/MSS.0b013e31823bf95c.

423. Zhang X, Yang Z, Du L, Xiong C, Wang Z, Pan Y, et al. Associations of accelerometer-measured physical activity, sedentary behavior with self-reported insomnia in older women: does pattern matter? Sleep Med. 2023 Apr;104:58–63. PMID: 36906996. doi: 10.1016/j.sleep.2023.02.015.

424. Zhong Q, Zhou R, Huang YN, Chen HW, Liu HM, Huang Z, et al. The independent and joint association of accelerometer-measured physical activity and sedentary time with dementia: a cohort study in the UK Biobank. Int J Behav Nutr Phys Act. 2023 May;20(1):59. PMID: 37198574. doi: 10.1186/s12966-023-01464-8.

425. Zhou W, Veliz PT, Smith EML, Chen W, Reddy RM, Larson JL. Comparison of pre-diagnosis ohysical activity and its correlates between lung and other cancer patients: accelerometer data from the UK Biobank prospective cohort. Int J Environ Res Public Health. 2023 Jan;20(2):1001. PMID: 36673757. doi: 10.3390/ijerph20021001.

426. Zijlstra WMH, Ploegstra MJ, Vissia-Kazemier T, Roofthooft MTR, Sarvaas GDM, Bartelds B, et al. Physical activity in pediatric pulmonary arterial hypertension measured by accelerometry. A candidate clinical endpoint. Am J Respir Crit Care Med. 2017 Jul;196(2):220–227. PMID: 28178426. doi: 10.1164/rccm.201608-1576OC.

427. Zisko N, Nauman J, Sandbakk SB, Aspvik NP, Salvesen Ø, Carlsen T, et al. Absolute and relative accelerometer thresholds for determining the association between physical activity and metabolic syndrome in the older adults: the Generation-100 study. BMC Geriatr. 2017 May;17(1):109. PMID: 28511695. doi: 10.1186/s12877-017-0497-1.

428. Zlatar ZZ, Hays CC, Mestre Z, Campbell LM, Meloy MJ, Bangen KJ, et al. Dose-dependent association of accelerometer-measured physical activity and sedentary time with brain perfusion in aging. Exp Gerontol. 2019 Oct;125:110679. PMID: 31382010. doi: 10.1016/j.exger.2019.110679.

**Articles categorized in the classification study (n=75):**

1. Agiovlasitis S, Ballenger BK, Schultz EE, Du Q, Motl RW. Calibration of hip accelerometers for measuring physical activity and sedentary behaviours in adults with Down syndrome. J Intellect Disabil Res. 2023 Feb;67(2):172–181. PMID: 36514268. doi: 10.1111/jir.13002.

2. Aguiar B, Silva J, Rocha T, Carneiro S, Sousa I. Monitoring physical activity and energy expenditure with smartphones. IEEE-EMBS International Conference on Biomedical and Health Informatics (BHI). 2014 Jun 01-04; Valencia, Spain. New York: IEEE; 2014. doi: 10.1109/BHI.2014.6864451.

3. Li A, Ji L, Wang S, Wu J. Physical activity classification using a single triaxial accelerometer based on HMM. IET International Conference on Wireless Sensor Network 2010 (IET-WSN 2010). 2010 Nov 15-17; Beijing, China. London: IET; 2010. doi: 10.1049/cp.2010.1045.

4. Aittasalo M, Vähä-Ypyä H, Vasankari T, Husu P, Jussila AM, Sievänen H. Mean amplitude deviation calculated from raw acceleration data: a novel method for classifying the intensity of adolescents’ physical activity irrespective of accelerometer brand. BMC Sports Sci Med Rehabil. 2015 Aug;7(1):18. PMID: 26251724. doi: 10.1186/s13102-015-0010-0.

5. Ali H, Messina E, Bisiani R. Subject-dependent physical activity recognition model framework with a semi-supervised clustering approach. 2013 European Modelling. 2013 Nov 20-22; Symposium Manchester, United Kingdom. New York: IEEE; 2013. doi: 10.1109/EMS.2013.7.

6. Anastasopoulou P, Tansella M, Stumpp J, Shammas L, Hey S. Classification of human physical activity and energy expenditure estimation by accelerometry and barometry. 2012 Annual International Conference of the IEEE Engineering in Medicine and Biology Society. 2012 Aug 28-Sep 01; San Diego, CA. New York: IEEE; 2012. PMID: 23367406. doi: 10.1109/EMBC.2012.6347471.

7. Bammann K, Thomson NK, Albrecht BM, Buchan DS, Easton C. Generation and validation of ActiGraph GT3X+ accelerometer cut-points for assessing physical activity intensity in older adults. The OUTDOOR ACTIVE validation study. PLoS One. 2021 Jun;16(6):e0252615. PMID: 34081715. doi: 10.1371/journal.pone.0252615.

8. Banker M, Song PXK. Supervised learning of physical activity features from functional accelerometer data. IEEE J Biomed Health Inform. 2023 Dec;27(12):5710–5721.PMID: 37738184. doi: 10.1109/JBHI.2023.3318205.

9. Baque E, Sakzewski L, Trost SG, Boyd RN, Barber L. Validity of accelerometry to measure physical activity intensity in children with an acquired brain injury. Pediatr Phys Ther. 2017 Oct;29(4):322–329. PMID: 28953176. doi: 10.1097/PEP.0000000000000439.

10. Bonomi AG, Plasqui G, Goris AH, Westerterp KR. Improving assessment of daily energy expenditure by identifying types of physical activity with a single accelerometer. J Appl Physiol. 2009 Sep;107(3):655–661. PMID: 19556460. doi: 10.1152/japplphysiol.00150.2009

11. Bonomi AG, Goris AH, Yin B, Westerterp KR. Detection of type, duration, and intensity of physical activity using an accelerometer. Med Sci Sports Exerc. 2009 Sep;41(9):1770–1777. PMID: 19657292. doi: 10.1249/MSS.0b013e3181a24536.

12. Chandler JL, Brazendale K, Beets MW, Mealing BA. Classification of physical activity intensities using a wrist‐worn accelerometer in 8–12‐year‐old children. Pediatr Obes. 2016 Apr;11(2):120–127. PMID: 25893950. doi: 10.1111/ijpo.12033.

13. Crouter SE, Flynn JI, Bassett DR. Estimating physical activity in youth using a wrist accelerometer. Med Sci Sports Exerc. 2015 May;47(5):944–951. PMID: 25207928. doi: 10.1249/MSS.0000000000000502.

14. Delgado-Gonzalo R, Celka P, Renevey Ph, Dasen S, Sola J, Bertschi M, et al. Physical activity profiling: activity-specific step counting and energy expenditure models using 3D wrist acceleration. 2015 37th Annual International Conference of the IEEE Engineering in Medicine and Biology Society (EMBC); 2015 Nov 25-29; Milan, Italy. New York: IEEE; 2015. doi: 10.1109/EMBC.2015.7320271.

15. Diaz KM, Krupka DJ, Chang MJ, Kronish IM, Moise N, Goldsmith J, et al. Wrist-based cut-points for moderate- and vigorous-intensity physical activity for the Actical accelerometer in adults. J Sports Sci. 2018 Jan;36(2):206–212. PMID: 28282744. doi: 10.1080/02640414.2017.1293279.

16. Dibben GO, Gandhi MM, Taylor RS, Dalal HM, Metcalf B, Doherty P, et al. Physical activity assessment by accelerometry in people with heart failure. BMC Sports Sci Med Rehabil. 2020 Aug;12(1):47. PMID: 32817798. doi: 10.1186/s13102-020-00196-7.

17. Diniz-Sousa F, Veras L, Ribeiro JC, Boppre G, Devezas V, Santos-Sousa H, et al. Accelerometry calibration in people with class II-III obesity: energy expenditure prediction and physical activity intensity identification. Gait Posture. 2020 Feb;76:104–109. PMID: 31756665. doi: 10.1016/j.gaitpost.2019.11.008.

18. Duncan MJ, Roscoe CMP, Faghy M, Tallis J, Eyre ELJ. Estimating physical activity in children aged 8–11 years using accelerometry: contributions from fundamental movement skills and different accelerometer placements. Front Physiol. 2019 Mar;10:242. PMID: 30936837. doi: 10.3389/fphys.2019.00242.

19. Ellis K, Kerr J, Godbole S, Lanckriet G, Wing D, Marshall S. A random forest classifier for the prediction of energy expenditure and type of physical activity from wrist and hip accelerometers. Physiol Meas. 2014 Dec;35(11):2191–2203. PMID: 25340969. doi: 10.1088/0967-3334/35/11/2191.

20. Evenson KR, Herring AH, Wen F. Accelerometry-assessed latent class patterns of physical activity and sedentary behavior with mortality. Am J Prev Med. 2017 Feb;52(2):135–143. PMID: 28109457. doi: 10.1016/j.amepre.2016.10.033.

21. Evenson KR, Wen F, Herring AH, Di C, LaMonte MJ, Tinker LF, et al. Calibrating physical activity intensity for hip-worn accelerometry in women age 60 to 91years: the women’s health initiative OPACH calibration study. Prev Med Rep. 2015;2:750–756. PMID: 26527313. doi: 10.1016/j.pmedr.2015.08.021.

22. Chuang FC, Wang JS, Yang YT, Kao TP. A wearable activity sensor system and its physical activity classification scheme. The 2012 International Joint Conference on Neural Networks (IJCNN); 2012 Jun 10-15; Brisbane, Australia. New York: IEEE; 2012. doi: 10.1109/IJCNN.2012.6252581.

23. Farooq M, Sazonov E. A novel wearable device for food intake and physical activity recognition. Sensors. 2016 Jul;16(7):1067. PMID: 27409622. doi: 10.3390/s16071067.

24. Gába A, Dygrýn J, Mitáš J, Jakubec L, Frömel K. Effect of accelerometer cut-off points on the recommended level of physical activity for obesity prevention in children. PLoS One. 2016 Oct;11(10):e0164282. PMID: 27723835. doi: 10.1371/journal.pone.0164282.

25. García-Massó X, Serra-Añó P, Gonzalez LM, Ye-Lin Y, Prats-Boluda G, Garcia-Casado J. Identifying physical activity type in manual wheelchair users with spinal cord injury by means of accelerometers. Spinal Cord. 2015 Oct;53(10):772–777. PMID: 25987002. doi: 10.1038/sc.2015.81.

26. Guthrie N, Bradlyn A, Thompson SK, Yen S, Haritatos J, Dillon F, et al. Development of an accelerometer-linked online intervention system to promote physical activity in adolescents. PLoS One. 2015 May;10(5):e0128639. PMID: 26010359. doi: 10.1371/journal.pone.0128639.

27. Hager ER, Gormley CE, Latta LW, Treuth MS, Caulfield LE, Black MM. Toddler physical activity study: laboratory and community studies to evaluate accelerometer validity and correlates. BMC Public Health. 2016 Sep;16(1):936. PMID: 27600404. doi: 10.1186/s12889-016-3569-9.

28. Hernando C, Hernando C, Collado EJ, Panizo N, Martinez-Navarro I, Hernando B. Establishing cut-points for physical activity classification using triaxial accelerometer in middle-aged recreational marathoners. PLoS One. 2018 Aug;13(8):e0202815. PMID: 30157271. doi: 10.1371/journal.pone.0202815.

29. Hikihara Y, Tanaka C, Oshima Y, Ohkawara K, Ishikawa-Takata K, Tanaka S. Prediction models discriminating between nonlocomotive and locomotive activities in children using a triaxial accelerometer with a gravity-removal physical activity classification algorithm. PLoS One. 2014 Apr;9(4):e94940. PMID: 24755646. doi: 10.1371/journal.pone.0094940.

30. Hossain SS, Lazar DM, Begum M. Ordinal statistical models of physical activity levels from accelerometer data. Int J Exerc Sci. 2021 Apr;14(7):338–357. PMID:34055179.

31. Jeng B, Cederberg KLJ, Lai B, Sasaki JE, Bamman MM, Motl RW. Wrist-based accelerometer cut-points for quantifying moderate-to-vigorous intensity physical activity in Parkinson’s disease. Gait Posture. 2022 Jan;91:235–239. PMID: 34749075. doi: 10.1016/j.gaitpost.2021.10.027.

32. Kos M, Bogdan M, Glynn NW, Harezlak J. Classification of human physical activity based on raw accelerometry data via spherical coordinate transformation. Stat Med. 2020 Sep;39(22):2901–2920. PMID: 32478905. doi: 10.1002/sim.8582.

33. Kumar D, Thanikkal A, Krishnamurthy P, Chen X, Zhang P. Accelerometer-based alcohol consumption detection from physical activity. 2021 17th International Conference on Wireless and Mobile Computing, Networking and Communications (WiMob); 2021 Oct 11-13; Bologna, Italy. New York: IEEE; 2021. doi: 10.1109/WiMob52687.2021.9606257

34. Lee SY, Kim SC, Gim JA, Park SJ, Seo SH, Kim SJ, et al. Accelerometer-derived physical activity analysis of elderly osteoarthritis patients. Musculoskelet Sci Practi. 2023 Aug;66:102808. PMID: 37352763. doi: 10.1016/j.msksp.2023.102808.

35. Li L, Nakamura T. An epidemiological sleep study based on a large-scale physical activity database. 2019 IEEE 1st Global Conference on Life Sciences and Technologies (LifeTech); 2019 Mar 12-14; Osaka, Japan. New York: IEEE; 2019. doi: 10.1109/LifeTech.2019.8883989.

36. Li S, Howard JT, Sosa ET, Cordova A, Parra-Medina D, Yin Z. Calibrating wrist-worn accelerometers for physical activity assessment in preschoolers: machine learning approaches. JMIR Form Res. 2020 Aug;4(8):e16727. PMID: 32667893. doi: 10.2196/16727.

37. Li T, Zong G, Peng P, Wang S, Cheng B. Accelerometer-measured physical activity and sample-based frailty in older women: does pattern really matter? Front Public Health. 2024 Jan;11:1304279. PMID: 38332942. doi: 10.3389/fpubh.2023.1304279.

38. Lin HP, Lynk N, Moore LL, Cabral HJ, Heffernan KS, Dumas AK, et al. A pragmatic approach to the comparison of wrist-based cutpoints of physical activity intensity for the MotionWatch8 accelerometer in children. PLoS One. 2020 Jun;15(6):e0234725. PMID: 32559207. doi: 10.1371/journal.pone.0234725.

39. Long, Xi, Bin Yin, Ronald M. Single-accelerometer-based daily physical activity classification. 2009 Annual International Conference of the IEEE Engineering in Medicine and Biology Society Minneapolis; 2009 Sep 03-06; Minneapolis, MN, USA New York: IEEE; 2009. PMID: 19965261. doi: 10.1109/IEMBS.2009.5334925.

40. Mannini A, Sabatini AM. Machine learning methods for classifying human physical activity from on-body accelerometers. Sensors. 2010 Feb;10(2):1154–1175. PMID: 22205862. doi: 10.3390/s100201154.

41. Mardini MT, Bai C, Wanigatunga AA, Saldana S, Casanova R, Manini TM. Age differences in estimating physical activity by wrist accelerometry using machine learning. Sensors. 2021 May;21(10):3352. PMID: 34065906. doi: 10.3390/s21103352.

42. McCracken LA, Ma JK, Voss C, Chan FH, Martin Ginis KA, West CR. Wrist accelerometry for physical activity measurement in individuals with spinal cord injury—a need for individually calibrated cut-points. Arch Phys Med Rehabil. 2018 Apr;99(4):684–689. PMID: 29222006. doi: 10.1016/j.apmr.2017.10.024.

43. McDevitt B, Connolly J, Duddy D, Doherty R, Condell J. Preliminary investigations of the validity and interinstrument reliability for classification of accelerometer physical activity cut-points against indirect caliometry in healthy adults. 2022 33rd Irish Signals and Systems Conference (ISSC); 2022 Jun 09-10; Cork, Ireland. New York: IEEE; 2022. doi: 10.1109/ISSC55427.2022.9826199.

44. McGarty AM, Penpraze V, Melville CA. Calibration and cross-validation of the ActiGraph wGT3X+ accelerometer for the estimation of physical activity intensity in children with intellectual disabilities. PLoS One. 2016 Oct;11(10):e0164928. PMID: 27760219. doi: 10.1371/journal.pone.0164928.

45. Midorikawa T, Tanaka S, Kaneko K, Koizumi K, Ishikawa‐Takata K, Futami J, et al. Evaluation of low‐intensity physical activity by triaxial accelerometry. Obesity. 2007 Dec;15(12):3031–3038. PMID: 18198312. doi: 10.1038/oby.2007.361.

46. Migueles JH, Cadenas-Sanchez C, Alcantara JMA, Leal-Martín J, Mañas A, Ara I, et al. Calibration and cross-validation of accelerometer cut-points to classify sedentary time and physical activity from hip and non-dominant and dominant wrists in older adults. Sensors. 2021 May;21(10):3326. PMID: 34064790. doi: 10.3390/s21103326.

47. Neil-Sztramko SE, Rafn BS, Gotay CC, Campbell KL. Determining activity count cut-points for measurement of physical activity using the Actiwatch2 accelerometer. Physiol Behav. 2017 May;173:95–100. PMID: 28108333. doi: 10.1016/j.physbeh.2017.01.026.

48. Nero H, Benka Wallén M, Franzén E, Ståhle A, Hagströmer M. Accelerometer cut points for physical activity assessment of older adults with Parkinson’s disease. PLoS One. 2015 Sep;10(9):e0135899. PMID: 26332765. doi: 10.1371/journal.pone.0135899.

49. Noor MHM, Salcic Z, Wang KIK. Dynamic sliding window method for physical activity recognition using a single tri-axial accelerometer. 2015 IEEE 10th Conference on Industrial Electronics and Applications (ICIEA); 2015 Nov 23; Auckland, New Zealand. New York: IEEE; 2015. doi: 10.1109/ICIEA.2015.7334092.

50. Nu UK, Touati T, Buddhadev S, Sun R, Smuck M, Song IHJ. Who is physically active? classification and analysis of physical activity using NHANES data. 2020 IEEE Symposium Series on Computational Intelligence (SSCI); 2021 Jan 05; Canberra, ACT, Australia. New York: IEEE; 2020. doi: 10.1109/SSCI47803.2020.9308353.

51. Ohkawara K, Oshima Y, Hikihara Y, Ishikawa-Takata K, Tabata I, Tanaka S. Real-time estimation of daily physical activity intensity by a triaxial accelerometer and a gravity-removal classification algorithm. Br J Nutr. 2011 Jun;105(11):1681–1691. PMID: 21262061. doi: 10.1017/S0007114510005441.

52. Pober DM, Staudenmayer J, Raphael C, Freedson PS. Development of novel techniques to classify physical activity mode using accelerometers. Med Sci Sports Exerc. 2006 Sep;38(9):1626–1634. PMID: 16960524. doi: 10.1249/01.mss.0000227542.43669.45.

53. Pulsford RM, Cortina-Borja M, Rich C, Kinnafick FE, Dezateux C, Griffiths LJ. Actigraph accelerometer-defined boundaries for sedentary behaviour and physical activity intensities in 7 year old children. PLoS One. 2011 Aug 11;6(8):e21822. PMID: 21853021. doi: 10.1371/journal.pone.0021822.

54. Qi J, Liang HN, Chen J, Peng X, Newcombe L, Yang P. A hybrid hierarchical model for accessing physical activity recognition towards free-living environments. 2020 IEEE Intl Conf on Parallel & Distributed Processing with Applications, Big Data & Cloud Computing, Sustainable Computing & Communications, Social Computing & Networking (ISPA/BDCloud/SocialCom/SustainCom); 2020 Dec 17-19; Exeter, United Kingdom. New York: IEEE; 2020. doi: 10.1109/ISPA-BDCloud-SocialCom-SustainCom51426.2020.00199.

55. Rahman HA, Carrault G, Ge D, Amoud H, Prioux J, Le Faucheur A, et al. Ambulatory physical activity representation and classification using spectral distances approach. 2015 International Conference on Advances in Biomedical Engineering (ICABME); 2015 Nov 16-18; Beirut, Lebanon. New York: IEEE; 2015. doi: 10.1109/ICABME.2015.7323253.

56. Roscoe CMP, James RS, Duncan MJ. Calibration of GENEActiv accelerometer wrist cut-points for the assessment of physical activity intensity of preschool aged children. Eur J Pediatr. 2017 Aug;176(8):1093–1098. PMID: 28674825. doi: 10.1007/s00431-017-2948-2.

57. Rubin DS, Huisingh-Scheetz M, Hung A, Ward RP, Nagele P, Arena R, Hedeker D. Accuracy of physical function questions to predict moderate-vigorous physical activity as measured by hip accelerometry. Anesthesiology. 2019 Nov;131(5):992–1003. PMID: 31490293. doi: 10.1097/ALN.0000000000002911.

58. Serra MC, Balraj E, DiSanzo BL, Ivey FM, Hafer-Macko CE, Treuth MS, et al. Validating accelerometry as a measure of physical activity and energy expenditure in chronic stroke. Top Stroke Rehabil. 2017 Jan 2;24(1):18–23. PMID: 27322733. doi: 10.1080/10749357.2016.1183866.

59. Sevil M, Rashid M, Maloney Z, Hajizadeh I, Samadi S, Askari MR, et al. Determining physical activity characteristics from wristband data for use in automated insulin delivery systems. IEEE Sens J. 2020 Nov;20(21):12859–12870. doi: 10.1109/JSEN.2020.3000772.

60. Shim J, Fleisch E, Barata F. Precise segmentation of U.S. adults from 24-hour wearable-based physical activity profiles using machine learning clustering. 2023 IEEE 11th International Conference on Healthcare Informatics (ICHI) Houston. 2023 Jun 26-29; TX, USA. New York: IEEE; 2023. doi: 10.1109/ICHI57859.2023.00083.

61. Shin YE, Choi WH, Shin TM. Physical activity recognition based on rotated acceleration data using quaternion in sedentary behavior : a preliminary study. 2014 36th Annual International Conference of the IEEE Engineering in Medicine and Biology Society; 2014 Aug 26-30; Chicago, US. New York: IEEE; 2014. doi: 10.1109/EMBC.2014.6944741

62. Shwetar Y, Huang Z, Veerubhotla A, Knezevic S, Hong E, Spungen AM, et al. Predicting physical activity intensity using raw accelerometer signals in manual wheelchair users with spinal cord injury. Spinal Cord. 2022 Feb;60(2):149–156. PMID: 34819608. doi: 10.1038/s41393-021-00728-z.

63. Siddique J, Aaby D, Montag SE, Sidney S, Sternfeld B, Welch WA, et al. Individualized relative-intensity physical activity accelerometer cut points. Med Sci Sports Exerc. 2020 Feb;52(2):398–407. PMID: 31524826. doi: 10.1249/MSS.0000000000002153.

64. Simón-Vicente L, Rivadeneyra-Posadas J, Soto-Célix M, Raya-González J, Castillo D, Calvo S, et al. Accelerometer cut-points for physical activity assessment in adults with mild to moderate Huntington’s disease: a cross-sectional multicentre study. Int J Environ Res Public Health. 2022 Nov;19(22):14834. PMID: 36429552. doi: 10.3390/ijerph192214834.

65. Stewart T, Narayanan A, Hedayatrad L, Neville J, Mackay L, Duncan S. A dual-accelerometer system for classifying physical activity in children and adults. Med Sci Sports Exerc. 2018 Dec;50(12):2595–2602. PMID: 30048411. doi: 10.1249/MSS.0000000000001717.

66. Thornton CB, Kolehmainen N, Nazarpour K. Using unsupervised machine learning to quantify physical activity from accelerometry in a diverse and rapidly changing population. PLOS Digit Health. 2023 Apr;2(4):e0000220. PMID: 37018183. doi: 10.1371/journal.pdig.0000220.

67. Trost SG, Brookes DSK, Ahmadi MN. Evaluation of wrist accelerometer cut-points for classifying physical activity intensity in youth. Front Digit Health. 2022 May;4:884307. PMID: 35585912. doi: 10.3389/fdgth.2022.884307.

68. Trumpeter NN, Lawman HG, Wilson DK, Pate RR, Van Horn M, Tate AK. Accelerometry cut points for physical activity in underserved African Americans. Int J Behav Nutr Phys Act. 2012;9(1):73. PMID: 22697280. doi: 10.1186/1479-5868-9-73.

69. Vähä-Ypyä H, Vasankari T, Husu P, Mänttäri A, Vuorimaa T, Suni J, et al. Validation of cut-points for evaluating the intensity of physical activity with accelerometry-based mean amplitude deviation (MAD). PLoS One. 2015 Aug;10(8):e0134813. PMID: 26292225. doi: 10.1371/journal.pone.0134813.

70. Vanhelst J, Béghin L, Turck D, Gottrand F. New validated thresholds for various intensities of physical activity in adolescents using the Actigraph accelerometer. Int J Rehabil Res. 2011 Jun;34(2):175–177. PMID: 20890210. doi: 10.1097/MRR.0b013e328340129e.

71. Vanhelst J, Hurdiel R, Mikulovic J, Bui-Xuân G, Fardy P, Theunynck D, et al. Validation of the Vivago wrist-worn accelerometer in the assessment of physical activity. BMC Public Health. 2012 Aug;12(1):690. PMID: 22913286. doi: 10.1186/1471-2458-12-690.

72. Wullems JA, Verschueren SMP, Degens H, Morse CI, Onambélé GL. Performance of thigh-mounted triaxial accelerometer algorithms in objective quantification of sedentary behaviour and physical activity in older adults. PLoS One. 2017 Nov;12(11):e0188215. PMID: 29155839. doi: 10.1371/journal.pone.0188215.

73. Xie C, Bi S, Dong M, Li L, Chi S. Walking recognition method for physical activity analysis system of child based on wearable accelerometer. 2017 IEEE International Conference on Robotics and Biomimetics (ROBIO); 2017 Dec 05-08; Macau, Macao. New York: IEEE; 2017. doi: 10.1109/ROBIO.2017.8324785.

74. Xiuxin Yang, Anh Dinh, Li Chen. Implementation of a wearerable real-time system for physical activity recognition based on Naive Bayes classifier. 2010 International Conference on Bioinformatics and Biomedical Technology; 2010 Apr 16-18; Chengdu, China. New York: IEEE; 2010. doi: 10.1109/ICBBT.2010.5479000.

75. Zhang S, Rowlands AV, Murray P, Hurst TL. Physical activity classification using the GENEA wrist-worn accelerometer. Med Sci Sports Exerc. 2012 Apr;44(4):742–748. PMID: 21988935. doi: 10.1249/MSS.0b013e31823bf95c.

**Articles categorized in the association study (n=342):**

1. Aadland E, Andersen LB, Resaland GK, Kvalheim OM. Interpretation of multivariate association patterns between multicollinear physical activity accelerometry data and cardiometabolic health in children—a tutorial. Metabolites. 2019 Jul;9(7):129. PMID: 31269708. doi: 10.3390/metabo9070129.

2. Aadland E, Kvalheim OM, Anderssen SA, Resaland GK, Andersen LB. Multicollinear physical activity accelerometry data and associations to cardiometabolic health: challenges, pitfalls, and potential solutions. Int J Behav Nutr Phys Act. 2019 Dec;16(1):74. PMID: 31455305. doi: 10.1186/s12966-019-0836-z.

3. Aadland E, Kvalheim OM, Hansen BH, Kriemler S, Ried-Larsen M, Wedderkopp N, et al. The multivariate physical activity signature associated with metabolic health in children and youth: an international children’s accelerometry database (ICAD) analysis. Prev Med. 2020 Dec;141:106266. PMID:33022325. doi: 10.1016/j.ypmed.2020.106266.

4. Aadland E, Steene-Johannessen J. The use of individual cut points from treadmill walking to assess free-living moderate to vigorous physical activity in obese subjects by accelerometry: is it useful? BMC Med Res Methodol. 2012 Nov;12(1):172. PMID:23152980. doi: 10.1186/1471-2288-12-172.

5. Agarwal V, Smuck M, Shah NH. Quantifying the relative change in physical activity after total knee arthroplasty using accelerometer based measurements. AMIA Jt Summits Transl Sci Proc. 2017 Jul;2017:463–472. PMID:28815146.

6. Agbaje AO. Associations of accelerometer‐based sedentary time, light physical activity and moderate‐to‐vigorous physical activity with resting cardiac structure and function in adolescents according to sex, fat mass, lean mass, BMI, and hypertensive status. Scand Med Sci Sports. 2023 Aug;33(8):1399–1411. PMID: 37035905. doi: 10.1111/sms.14365.

7. Agbaje AO. Mediating effect of fat mass, lean mass, blood pressure and insulin resistance on the associations of accelerometer-based sedentary time and physical activity with arterial stiffness, carotid IMT and carotid elasticity in 1574 adolescents. J Hum Hypertens. 2024 Feb. PMID: 38409590. doi: 10.1038/s41371-024-00905-6.

8. Agbaje AO, Perng W, Tuomainen TP. Effects of accelerometer-based sedentary time and physical activity on DEXA-measured fat mass in 6059 children. Nat Commun. 2023 Dec;14(1):8232. PMID: 38086810. doi: 10.1038/s41467-023-43316-w.

9. Ahn HJ, Choi EK, Rhee TM, Choi J, Lee KY, Kwon S, et al. Accelerometer-derived physical activity and the risk of death, heart failure, and stroke in patients with atrial fibrillation: a prospective study from UK Biobank. Br J Sports Med. 2024 Feb;bjsports-2023-106862. PMID: 38418213. doi: 10.1136/bjsports-2023-106862.

10. Ajja R, Wikkeling-Scott LF, Brazendale K, Hijazi R, Abdulle A. Accelerometer measured physical activity patterns of children during segmented school day in Abu Dhabi. BMC Pediatr. 2021 Apr;21(1):182. PMID: 33865344. doi: 10.1186/s12887-021-02639-7.

11. Albrecht BM, Stalling I, Recke C, Bammann K. Accelerometer-assessed outdoor physical activity is associated with meteorological conditions among older adults: Cross-sectional results from the OUTDOOR ACTIVE study. PLoS One. 2020 Jan;15(1):e0228053. PMID: 31978178. doi: 10.1371/journal.pone.0228053.

12. Allen CL, Montes E, Hoang T, Romo T, Peña J, Navarro J. Can stereotype threat and lift visual messages affect subsequent physical activity? Evidence from a controlled experiment using accelerometers. Health Commun. 2023 Nov;1–12. PMID: 37941378.

13. Alsamman RA, Shousha TM, Faris ME, Abdelrahim DN, Arumugam A. Association of sociodemographic, anthropometric, and sleep quality factors with accelerometer-measured sitting and physical activity times among Emirati working women during the COVID-19 pandemic: a cross-sectional study. Womens Health. 2024 Jan-Dec;20:17455057231225539. PMID: 38279820. doi: 10.1177/17455057231225539.

14. Andersen MB, Ostenfeld EB, Fuglsang J, Møller M, Daugaard M, Ovesen PG. Maternal prepregnancy body mass index and physical activity during pregnancy assessed by accelerometer. Am J Obstet Gynecol MFM. 2020 Nov;2(4):100182. PMID: 33345908. doi: 10.1016/j.ajogmf.2020.100182.

15. Andersson C, Lyass A, Larson MG, Spartano NL, Vita JA, Benjamin EJ, et al. Physical activity measured by accelerometry and its associations with cardiac structure and vascular function in young and middle‐aged adults. J Am Heart Assoc. 2015 Mar;4(3):e001528. PMID: 25792127. doi: 10.1161/JAHA.114.001528.

16. Appelqvist-Schmidlechner K, Raitanen J, Vasankari T, Kyröläinen H, Häkkinen A, Honkanen T, et al. Relationship between accelerometer-based physical activity, sedentary behavior, and mental health in young Finnish men. Front Public Health. 2022 Feb;10:820852. PMID: 35252097. doi: 10.3389/fpubh.2022.820852.

17. Armstrong B, Covington LB, Hager ER, Black MM. Objective sleep and physical activity using 24-hour ankle-worn accelerometry among toddlers from low-income families. Sleep Health. 2019 Oct;5(5):459–465. PMID: 31171491. doi: 10.1016/j.sleh.2019.04.005.

18. Arteaga D, Donnelly T, Crum K, Markham L, Killian M, Burnette WB, et al. Assessing physical activity using accelerometers in youth with duchenne muscular dystrophy. J Neuromuscul Dis. 2020 Jun;7(3):331–342. PMID: 32417792. doi: 10.3233/JND-200478.

19. Augustin NH, Mattocks C, Cooper AR, Ness AR, Faraway JJ. Modelling fat mass as a function of weekly physical activity profiles measured by Actigraph accelerometers. Physiol Meas. 2012 Nov;33(11):1831–1839. PMID: 23110964. doi: 10.1088/0967-3334/33/11/1831.

20. Ayabe M, Kumahara H. Effect of handling breaks on estimation of heart rate responses to bouts of physical activity among young women: An accelerometer research issue. Gait Posture. 2020 Sep;81:1–6. PMID: 32645577. doi: 10.1016/j.gaitpost.2020.06.032.

21. Baldanzi G, Sayols-Baixeras S, Ekblom-Bak E, Ekblom Ö, Dekkers KF, Hammar U, et al. Accelerometer-based physical activity is associated with the gut microbiota in 8416 individuals in SCAPIS. EBioMedicine. 2024 Feb;100:104989. PMID: 38301483. doi: 10.1016/j.ebiom.2024.104989.

22. Ballin M, Antonsson O, Rosenqvist V, Nordström P, Nordström A. Association of dog ownership with accelerometer-measured physical activity and daily steps in 70-year-old individuals: a population-based cross-sectional study. BMC Public Health. 2021 Dec;21(1):2313. PMID: 34933682. doi: 10.1186/s12889-021-12401-4.

23. Bangen KJ, Calcetas AT, Thomas KR, Wierenga C, Smith CN, Bordyug M, et al. Greater accelerometer-measured physical activity is associated with better cognition and cerebrovascular health in older adults. J Int Neuropsychol Soc. 2023 Nov;29(9):859–869. PMID: 36789631. doi: 10.1017/S1355617723000140.

24. Barker J, Smith Byrne K, Doherty A, Foster C, Rahimi K, Ramakrishnan R, et al. Physical activity of UK adults with chronic disease: cross-sectional analysis of accelerometer-measured physical activity in 96 706 UK Biobank participants. Int J Epidemiol. 2019 Aug;48(4):1167-1174. PMID: 30721947. doi: 10.1093/ije/dyy294.

25. Barrett S, Begg S, O′Halloran P, Kingsley M. The effect of a physical activity coaching intervention on accelerometer-measured sedentary behaviours in insufficiently physically active ambulatory hospital patients. Int J Environ Res Public Health. 2021 May;18(11):5543. PMID: 34067292. doi: 10.3390/ijerph18115543.

26. Baumann S, Groß S, Voigt L, Ullrich A, Weymar F, Schwaneberg T, et al. Pitfalls in accelerometer‐based measurement of physical activity: the presence of reactivity in an adult population. Scand J Med Sci Sports. 2018 Mar;28(3):1056–1063. PMID: 28921747. doi: 10.1111/sms.12977.

27. Baumann S, Guertler D, Weymar F, Bahls M, Dörr M, Van Den Berg N, et al. Do accelerometer-based physical activity patterns differentially affect cardiorespiratory fitness? A growth mixture modeling approach. J Behav Med. 2020 Feb;43(1):99–107. PMID: 31190167. doi: 10.1007/s10865-019-00069-6.

28. Bayartai ME, Määttä J, Karppinen J, Oura P, Takatalo J, Auvinen J, et al. Association of accelerometer-measured physical activity, back static muscular endurance and abdominal obesity with radicular pain and non-specific low back pain. Sci Rep. 2023 May;13(1):7736. PMID: 37173344. doi: 10.1038/s41598-023-34733-4.

29. Bayartai ME, Taulaniemi A, Tokola K, Vähä-Ypyä H, Parkkari J, Husu P, et al. Role of the interaction between lumbar kinematics and accelerometer-measured physical activity in bodily pain, physical functioning and work ability among health care workers with low back pain. J Electromyogr Kinesiol. 2023 Apr;69:102744. PMID: 36680881. doi: 10.1016/j.jelekin.2023.102744.

30. Benadjaoud MA, Menai M, Van Hees VT, Zipunnikov V, Regnaux JP, Kivimäki M, et al. The association between accelerometer-assessed physical activity and respiratory function in older adults differs between smokers and non-smokers. Sci Rep. 2019 Jul;9(1):10270. PMID: 31311982. doi: 10.1038/s41598-019-46771-y.

31. Bergh IH, Van Stralen MM, Grydeland M, Bjelland M, Lien N, Andersen LF, et al. Exploring mediators of accelerometer assessed physical activity in young adolescents in the HEalth in Adolescents study – a group randomized controlled trial. BMC Public Health. 2012 Sep;12(1):814. PMID: 22995043. doi: 10.1186/1471-2458-12-814.

32. Berninger N, Knell G, Gabriel KP, Plasqui G, Crutzen R, Ten Hoor G. Bidirectional day-to-day associations of reported sleep duration with accelerometer measured physical activity and sedentary time among Dutch adolescents: an observational study. J Meas Physical Behav. 2020 Dec;3(4):304–314. PMID: 35665029. doi: 10.1123/jmpb.2020-0010.

33. Biswas A, Chen C, Prince SA, Smith PM, Mustard CA. Daily accelerometer-measured physical activity patterns and associations with cardiometabolic health among Canadian working adults. Health Rep. 2023 Mar;34(3):15-29. PMID: 36921073. doi: 10.25318/82-003-X202300300002-eng.

34. Bonomi AG, Goris AH, Yin B, Westerterp KR. Detection of type, duration, and intensity of physical activity using an accelerometer. Med Sci Sports Exerc. 2009 Sep;41(9):1770–1777. PMID: 19657292. doi: 10.1249/MSS.0b013e3181a24536.

35. Browne RAV, Macêdo GAD, Cabral LLP, Oliveira GTA, Vivas A, Fontes EB, et al. Initial impact of the COVID-19 pandemic on physical activity and sedentary behavior in hypertensive older adults: an accelerometer-based analysis. Exp Gerontol. 2020 Dec;142:111121. PMID: 33132145. doi: 10.1016/j.exger.2020.111121.

36. Carlson RH, Huebner DR, Hoarty CA, Whittington J, Haynatzki G, Balas MC, et al. Treadmill gait speeds correlate with physical activity counts measured by cell phone accelerometers. Gait Posture. 2012 Jun;36(2):241–248. PMID: 22475727. doi: 10.1016/j.gaitpost.2012.02.025.

37. Carvalho EV, Reboredo MM, Gomes EP, Teixeira DR, Roberti NC, Mendes JO, et al. Physical activity in daily life assessed by an accelerometer in kidney transplant recipients and hemodialysis patients. Transplant Proc. 2014 Jul-Aug;46(6):1713–1717. PMID: 25131019. doi: 10.1016/j.transproceed.2014.05.019.

38. Cassidy S, Fuller H, Chau J, Catt M, Bauman A, Trenell MI. Accelerometer-derived physical activity in those with cardio-metabolic disease compared to healthy adults: a UK Biobank study of 52,556 participants. Acta Diabetol. 2018 Sep;55(9):975–979. PMID: 29808390. doi: 10.1007/s00592-018-1161-8.

39. Chan JA, Bosma H, Drosinou C, Timmermans EJ, Savelberg H, Schaper N, et al. Association of perceived and objective neighborhood walkability with accelerometer‐measured physical activity and sedentary time in the Maastricht study. Scand Med Sci Sports. 2023 Nov;33(11):2313–2322. PMID: 37489093. doi: 10.1111/sms.14455.

40. Chen GC, Qi Q, Hua S, Moon JY, Spartano NL, Vasan RS, et al. Accelerometer-assessed physical activity and incident diabetes in a population covering the adult life span: the Hispanic community health study/study of Latinos. Am J Clin Nutr. 2020 Nov;112(5):1318–1327. PMID: 32910816. doi: 10.1093/ajcn/nqaa232.

41. Chen M, Landré B, Marques-Vidal P, Van Hees VT, Van Gennip ACE, Bloomberg M, et al. Identification of physical activity and sedentary behaviour dimensions that predict mortality risk in older adults: development of a machine learning model in the Whitehall II accelerometer sub-study and external validation in the CoLaus study. EClinicalMedicine. 2023 Jan;55:101773. PMID: 36568684. doi: 10.1016/j.eclinm.2022.101773.

42. Chen T, Honda T, Chen S, Narazaki K, Kumagai S. Dose–response association between accelerometer-assessed physical activity and incidence of functional disability in older Japanese adults: a 6-year prospective study. J Geront A Biol Sci Med Sci. 2020 Sep;75(9):1763–1770. PMID: 32134454. doi: 10.1093/gerona/glaa046.

43. Cheng TS, Brage S, Van Sluijs EMF, Ong KK. Pre-pubertal accelerometer-assessed physical activity and timing of puberty in British boys and girls: the Millennium cohort study. Int J Epidemiol. 2023 Oct;52(5):1316–1327. PMID: 37208864. doi: 10.1093/ije/dyad063.

44. Choe EY, He D, Sun G. Trading-off transit and non-transit physical activity among older people: evidence from longitudinal accelerometer data of a natural experiment study. J Urban Health. 2023 Apr;100(2):408–417. PMID: 36656439. doi: 10.1007/s11524-022-00709-4.

45. Cochrane SK, Chen S, Fitzgerald JD, Dodson JA, Fielding RA, King AC, et al. Association of accelerometry‐measured physical activity and cardiovascular events in mobility‐Limited older adults: the LIFE (lifestyle interventions and independence for elders) study. J Am Heart Assoc. 2017 Dec;6(12):e007215. PMID: 29197830. doi: 10.1161/JAHA.117.007215.

46. Cohn-Schwartz E, Khalaila R. Accelerometer-assessed physical activity and cognitive performance among European adults aged 50+: the mediating effects of social contacts and depressive symptoms. Healthcare. 2022 Nov;10(11):2279. PMID: 36421603. doi: 10.3390/healthcare10112279.

47. Cooper AR, Goodman A, Page AS, Sherar LB, Esliger DW, Van Sluijs EM, et al. Objectively measured physical activity and sedentary time in youth: the International children’s accelerometry database (ICAD). Int J Behav Nutr Phys Act. 2015 Sep;12(1):113. PMID: 26377803. doi: 10.1186/s12966-015-0274-5.

48. Corcoran MP, Chui KK, White DK, Reid KF, Kirn D, Nelson ME, et al. Accelerometer assessment of physical activity and its association with physical function in older adults residing at assisted care facilities. J Nutr Health Aging. 2016 Jul;20(7):752–758. PMID: 27499309. doi: 10.1007/s12603-015-0640-7.

49. Corder K, Sharp SJ, Atkin AJ, Andersen LB, Cardon G, Page A, et al. Age-related patterns of vigorous-intensity physical activity in youth: the international children’s accelerometry database. Prev Med Rep. 2016 May;4:17–22. PMID: 27413656. doi: 10.1016/j.pmedr.2016.05.006.

50. D’Silva A, Bebb G, Boyle T, Johnson ST, Vallance JK. Demographic and clinical correlates of accelerometer assessed physical activity and sedentary time in lung cancer survivors. Psychooncology. 2018 Mar;27(3):1042–1049. PMID: 29226994. doi: 10.1002/pon.4608.

51. Da Silva IC, Van Hees VT, Ramires VV, Knuth AG, Bielemann RM, Ekelund U, et al. Physical activity levels in three Brazilian birth cohorts as assessed with raw triaxial wrist accelerometry. Int J Epidemiol. 2014 Dec;43(6):1959–1968. PMID: 25361583. doi: 10.1093/ije/dyu203.

52. Da Silva SG, Evenson KR, Da Silva ICM, Mendes MA, Domingues MR, Da Silveira MF, et al. Correlates of accelerometer‐assessed physical activity in pregnancy—the 2015 Pelotas (Brazil) birth cohort study. Scand Med Sci Sports. 2018 Aug;28(8):1934–1945. PMID: 29542188. doi: 10.1111/sms.13083.

53. Dankel SJ, Loenneke JP, Loprinzi PD. Combined associations of muscle-strengthening activities and accelerometer-assessed physical activity on multimorbidity: findings from NHANES. Am J Health Promot. 2017 Jul;31(4):274–277. PMID: 26730562. doi: 10.4278/ajhp.150520-QUAN-894.

54. DasMahapatra P, Chiauzzi E, Bhalerao R, Rhodes J. Free-living physical activity monitoring in adult US patients with multiple sclerosis using a consumer wearable device. Digit Biomark. 2018 Apr;2(1):47–63. PMID: 32095756. doi: 10.1159/000488040.

55. Davoudi A, Ozrazgat-Baslanti T, Tighe PJ, Bihorac A, Rashidi P. Pain and physical activity association in Critically Ill patients. 2020 42nd Annual International Conference of the IEEE Engineering in Medicine & Biology Society (EMBC) Montreal, QC, Canada: IEEE; 2020. p. 5696–5699. doi: 10.1109/EMBC44109.2020.9176227.

56. De Vries EA, Heijenbrok-Kal MH, Van Kooten F, Giurgiu M, Ribbers GM, Van Den Berg-Emons RJG, et al. Unraveling the interplay between daily life fatigue and physical activity after subarachnoid hemorrhage: an ecological momentary assessment and accelerometry study. J Neuroeng Rehabil. 2023 Sep;20(1):127. PMID: 37752550. doi: 10.1186/s12984-023-01241-5.

57. Del Pozo Cruz B, Ahmadi M, Inan‐Eroglu E, Huang B, Stamatakis E. Prospective associations of accelerometer‐assessed physical activity with mortality and incidence of cardiovascular disease among adults with hypertension: the UK Biobank study. J Am Heart Assoc. 2022 Mar;11(6):e023290. PMID: 35253444. doi: 10.1161/JAHA.121.023290.

58. Dennison CA, Legge SE, Bracher-Smith M, Menzies G, Escott-Price V, Smith DJ, et al. Association of genetic liability for psychiatric disorders with accelerometer-assessed physical activity in the UK Biobank. PLoS One. 2021 Mar;16(3):e0249189. PMID: 33770123. doi: 10.1371/journal.pone.0249189.

59. Dijk-Huisman HCV, Senden R, Smeets MHH, Marcellis RGJ, Magdelijns FJH, Lenssen AF. The effect of a smartphone app with an accelerometer on the physical activity behavior of hospitalized patients: a randomized controlled trial. Sensors. 2023 Oct;23(21):8704. PMID: 37960404. doi: 10.3390/s23218704.

60. Do B, Zink J, Mason TB, Belcher BR, Dunton GF. Physical activity and sedentary time among mothers of school-aged children: differences in accelerometer-derived pattern metrics by demographic, employment, and household factors. Women Health Issues. 2022 Sep-Oct;32(5):490–498. PMID: 35491346. doi: 10.1016/j.whi.2022.03.005.

61. Doherty A, Jackson D, Hammerla N, Plötz T, Olivier P, Granat MH, et al. Large scale population assessment of physical activity using wrist worn accelerometers: the UK Biobank study. PLoS One 2017 Feb;12(2):e0169649. PMID: 28146576. doi: 10.1371/journal.pone.0169649.

62. Dohrn IM, Sjöström M, Kwak L, Oja P, Hagströmer M. Accelerometer-measured sedentary time and physical activity—a 15 year follow-up of mortality in a Swedish population-based cohort. J Sci Med Sport. 2018 Jul;21(7):702–707. PMID: 29128418. doi: 10.1016/j.jsams.2017.10.035.

63. Dohrn IM, Welmer AK, Hagströmer M. Accelerometry-assessed physical activity and sedentary time and associations with chronic disease and hospital visits - a prospective cohort study with 15 years follow-up. Int J Behav Nutr Phys Act. 2019 Dec;16(1):125. PMID: 31818303. doi: 10.1186/s12966-019-0878-2.

64. Domazet SL, Tarp J, Thomsen RW, Højlund K, Stidsen JV, Brønd JC, et al. Accelerometer-derived physical activity and sedentary behaviors in individuals with newly diagnosed type 2 diabetes: a cross-sectional study from the Danish nationwide DD2 cohort. Front Sports Act Living. 2023 Jan;4:1089579. PMID: 36761371. doi: 10.3389/fspor.2022.1089579.

65. Domingos C, Picó-Pérez M, Magalhães R, Moreira M, Sousa N, Pêgo JM, et al. Free-living physical activity measured with a wearable device is associated with larger hippocampus volume and greater functional connectivity in healthy older adults: an observational, cross-sectional study in northern Portugal. Front Aging Neurosci. 2021 Nov;13:729060. PMID: 34916921. doi: 10.3389/fnagi.2021.729060.

66. Dougherty EN, Randall I, Haedt-Matt AA, Pila E, Smith K, Wang S, et al. Accelerometer-based physical activity and shape and weight concerns among youth with overweight and obesity: a pilot exploratory ecological momentary assessment study. Child Obes. 2023 May. PMID: 37253094. doi: 10.1089/chi.2022.0236.

67. Douma JAJ, Verdonck-de Leeuw IM, Leemans CR, Jansen F, Langendijk JA, Baatenburg De Jong RJ, et al. Demographic, clinical and lifestyle-related correlates of accelerometer assessed physical activity and fitness in newly diagnosed patients with head and neck cancer. Acta Oncol. 2020 Mar;59(3):342–350. PMID: 31608747. doi: 10.1080/0284186X.2019.1675906.

68. Dugas LR, Kliethermes S, Plange-Rhule J, Tong L, Bovet P, Forrester TE, et al. Accelerometer-measured physical activity is not associated with two-year weight change in African-origin adults from five diverse populations. PeerJ. 2017 Jan;5:e2902. PMID: 28133575. doi: 10.7717/peerj.2902.

69. Edwards MK, Loprinzi PD. Associations between accelerometer-assessed sedentary behavior, physical activity and objectively-measured cardiorespiratory fitness with red blood cell distribution width. Int J Cardiol. 2016 Oct;221:755–758. PMID: 27428316. doi: 10.1016/j.ijcard.2016.07.137.

70. Ehakeem A, Gregson CL, Tobias JH, Lawlor DA. Age at puberty and accelerometer-measured physical activity: findings from two independent UK cohorts. Ann of Hum Biol. 2020 Jun;47(4):391–399. PMID: 32380867. doi: 10.1080/03014460.2019.1707284.

71. Ekblom-Bak E, Börjesson M, Ekblom Ö, Angerås O, Bergman F, Berntsson C, et al. Accelerometer derived physical activity and subclinical coronary and carotid atherosclerosis: cross-sectional analyses in 22 703 middle-aged men and women in the SCAPIS study. BMJ Open. 2023 Nov;13(11):e073380. PMID: 37996228. doi: 10.1136/bmjopen-2023-073380.

72. Ekblom‐Bak E, Börjesson M, Bergman F, Bergström G, Dahlin‐Almevall A, Drake I, et al. Accelerometer derived physical activity patterns in 27.890 middle‐aged adults: The SCAPIS cohort study. Scand Med Sci Sports. 2022 May;32(5):866–880. PMID: 35080270. doi: 10.1111/sms.14131.

73. Ekstedt M, Nyberg G, Ingre M, Ekblom Ö, Marcus C. Sleep, physical activity and BMI in six to ten-year-old children measured by accelerometry: a cross-sectional study. Int J Behav Nutr Phys Act. 2013 Jun;10(1):82. PMID: 23800204. doi: 10.1186/1479-5868-10-82.

74. Elhakeem A, Hannam K, Deere KC, Hartley A, Clark EM, Moss C, et al. Associations of lifetime walking and weight bearing exercise with accelerometer-measured high impact physical activity in later life. Prev Med Rep. 2017 Dec;8:183–189. PMID: 29134173. doi: 10.1016/j.pmedr.2017.10.011.

75. Evenson KR, Herring AH, Wen F. Accelerometry-assessed latent class patterns of physical activity and sedentary behavior with mortality. Am J Prev Med. 2017 Feb;52(2):135–143. PMID: 28109457. doi: 10.1016/j.amepre.2016.10.033.

76. Ezeugwu V, Klaren RE, A Hubbard E, Manns PT, Motl RW. Mobility disability and the pattern of accelerometer-derived sedentary and physical activity behaviors in people with multiple sclerosis. Prev Med Rep. 2015 Apr;2:241–246. PMID: 26844077. doi: 10.1016/j.pmedr.2015.03.007.

77. Fairclough SJ, Rowlands AV, Del Pozo Cruz B, Crotti M, Foweather L, Graves LEF, et al. Reference values for wrist-worn accelerometer physical activity metrics in England children and adolescents. Int J Behav Nutr Phys Act. 2023 Mar;20(1):35. PMID: 36964597. doi: 10.1186/s12966-023-01435-z.

78. Feinglass J, Lee J, Semanik P, Song J, Dunlop D, Chang R. The effects of daily weather on accelerometer-measured physical activity. J Phys Act Health. 2011 Sep;8(7):934–943. PMID: 21885884. doi: 10.1123/jpah.8.7.934.

79. Fernández-Verdejo R, Alcantara JMA, Galgani JE, Acosta FM, Migueles JH, Amaro-Gahete FJ, et al. Deciphering the constrained total energy expenditure model in humans by associating accelerometer-measured physical activity from wrist and hip. Sci Rep. 2021 Jun;11(1):12302. PMID: 34112912. doi: 10.1038/s41598-021-91750-x.

80. Foldager F, Jørgensen PB, Tønning LU, Petersen ET, Jakobsen SS, Vainorius D, et al. The relationship between muscle power, functional performance, accelerometer-based measurement of physical activity and patient-reported outcomes in patients with hip osteoarthritis: a cross-sectional study. Musculoskele Sci Pract. 2022 Dec;62:102678. PMID: 36335851. doi: 10.1016/j.msksp.2022.102678.

81. Foong YC, Chherawala N, Aitken D, Scott D, Winzenberg T, Jones G. Accelerometer‐determined physical activity, muscle mass, and leg strength in community‐dwelling older adults. J Cachexia Sarcopenia Muscle. 2016 Jun;7(3):275–283. PMID: 27239404. doi: 10.1002/jcsm.12065.

82. Fox FAU, Diers K, Lee H, Mayr A, Reuter M, Breteler MMB, et al. Association between accelerometer-derived physical activity measurements and brain structure: a population-based cohort study. Neurology. 2022 Sep;99(11). PMID: 35918154. doi: 10.1212/WNL.0000000000200884.

83. Francis SL, Simmering JE, Polgreen LA, Evans NJ, Hosteng KR, Carr LJ, et al. Gamifying accelerometer use increases physical activity levels of individuals pre-disposed to type II diabetes. Prev Med Rep. 2021 May;23:101426. PMID: 34178586. doi: 10.1016/j.pmedr.2021.101426.

84. Frehlich L, Turin TC, Doyle-Baker PK, Lang JJ, McCormack GR. Mediation analysis of the associations between neighbourhood walkability and greenness, accelerometer-measured physical activity, and health-related fitness in urban dwelling Canadians. Prev Med. 2024 Jan;178:107792. PMID: 38052331. doi: 10.1016/j.ypmed.2023.107792.

85. Fridolfsson J, Arvidsson D, Ekblom-Bak E, Ekblom Ö, Bergström G, Börjesson M. Accelerometer-measured absolute versus relative physical activity intensity: cross-sectional associations with cardiometabolic health in midlife. BMC Public Health. 2023 Nov;23(1):2322. PMID: 37996871. doi: 10.1186/s12889-023-17281-4.

86. Fridolfsson J, Börjesson M, Ekblom-Bak E, Ekblom Ö, Arvidsson D. Stronger association between high intensity physical activity and cardiometabolic health with improved assessment of the full intensity range using accelerometry. Sensors. 2020 Feb;20(4):1118. PMID: 32085652. doi: 10.3390/s20041118.

87. Frith E, Loprinzi PD. Accelerometer-assessed light-intensity physical activity and mortality among those with mobility limitations. Disabil Health J. 2018 Apr;11(2):298–300. PMID: 28877860. doi: 10.1016/j.dhjo.2017.08.007.

88. Full KM, Moran K, Carlson J, Godbole S, Natarajan L, Hipp A, et al. Latent profile analysis of accelerometer-measured sleep, physical activity, and sedentary time and differences in health characteristics in adult women. PLoS One, 2019 Jun;14(6):e0218595. PMID: 31247051. doi: 10.1371/journal.pone.0218595.

89. Full KM, Whitaker KM, Pettee Gabriel K, Lewis CE, Sternfeld B, Sidney S, et al. Cardiovascular risk and functional burden at midlife: Prospective associations of isotemporal reallocations of accelerometer-measured physical activity and sedentary time in the CARDIA study. Prev Med. 2021 Sep;150:106626. PMID: 34019927. doi: 10.1016/j.ypmed.2021.106626.

90. Gába A, Dygrýn J, Mitáš J, Jakubec L, Frömel K. Effect of Accelerometer Cut-Off Points on the Recommended Level of Physical Activity for Obesity Prevention in Children. PLoS One. 2016 Oct;11(10):e0164282. PMID: 27723835. doi: 10.1371/journal.pone.0164282.

91. Gába A, Mitáš J, Jakubec L. Associations between accelerometer-measured physical activity and body fatness in school-aged children. Environ Health Prev Med. 2017 Apr;22(1):43. PMID: 29165128. doi: 10.1186/s12199-017-0629-4.

92. Gao Y, Hua S, Mok Y, Salameh M, Qi Q, Chen G, et al. Joint associations of peripheral artery disease and accelerometry-based physical activity with mortality: the Hispanic community health study/study of Latinos (HCHS/SOL). Atherosclerosis. 2022 Apr;347:55–62. PMID: 35334347. doi: 10.1016/j.atherosclerosis.2022.03.008.

93 Garay JL, Barreira TV, Wang Q, Brutsaert TD. Size at birth and accelerometer‐measured physical activity or sedentary behavior in healthy term‐born adults. Am J Hum Biol. 2022 Jun;34(6):e23717. PMID: 34978113. doi: 10.1002/ajhb.23717.

94. Ghorbani S, Afshari M, Eckelt M, Dana A, Bund A. Associations between physical activity and mental health in Iranian adolescents during the COVID-19 pandemic: an accelerometer-based study. Children. 2021 Nov;8(11):1022. PMID: 34828736. doi: 10.3390/children8111022.

95. Glass NL, Bellettiere J, Jain P, LaMonte MJ, LaCroix AZ, Women’s Health Initiative. Evaluation of light physical activity measured by accelerometry and mobility disability during a 6-year follow-up in older women. JAMA Netw Open. 2021 Feb;4(2):e210005. PMID: 33620446. doi: 10.1001/jamanetworkopen.2021.0005.

96. Gråstén A, Huhtiniemi M, Hakonen H, Jaakkola T. Development of accelerometer‐based light to vigorous physical activity in fitness profiles of school‐aged children. Scand Med Sci Sports. 2021 Dec;31(12):2343–2355. PMID: 34536979. doi: 10.1111/sms.14056.

97. Gråstén A, Huhtiniemi M, Kolunsarka I, Jaakkola T. Developmental associations of accelerometer measured moderate-to-vigorous physical activity and sedentary time with cardiorespiratory fitness in schoolchildren. J Sci Med Sport. 2022 Nov;25(11):884–889. PMID: 36137921. doi: 10.1016/j.jsams.2022.08.015.

98. Graves JL, Qiao Y, Moored KD, Boudreau RM, Venditti EM, Krafty RT, et al. Profiles of accelerometry-derived physical activity are related to perceived physical fatigability in older adults. Sensor. 2021 Mar 2;21(5):1718. PMID: 33801352. doi: 10.3390/s21051718.

99. Gupta N, Dencker-Larsen S, Lund Rasmussen C, McGregor D, Rasmussen CDN, Thorsen SV, et al. The physical activity paradox revisited: a prospective study on compositional accelerometer data and long-term sickness absence. Int J Behav Nutr Phys Act. 2020 Jul;17(1):93. PMID: 32690043. doi: 10.1186/s12966-020-00988-7.

100. Gupta N, Heiden M, Aadahl M, Korshøj M, Jørgensen MB, Holtermann A. What is the effect on obesity indicators from replacing prolonged sedentary time with brief sedentary bouts, standing and different types of physical activity during working days? a cross-sectional accelerometer-based study among blue-collar workers. PLoS One. 2016 May;11(5):e0154935. PMID: 27187777. doi: 10.1371/journal.pone.0154935.

101. Gutiérrez-Hervás A, Cortés-Castell E, Juste-Ruíz M, Palazón-Bru A, Gil-Guillén V, Rizo-Baeza M. Physical activity values in two-to-seven-year-old children measured by accelerometer over five consecutives 24-hour days. Nutr Hosp. 2018 Jun 27;35(3):527-532. PMID: 29974757. doi: 10.20960/nh.1403.

102. Ha L, Wakefield CE, Diaz C, Mizrahi D, Signorelli C, Yacef K, et al. Patterns of physical activity and sedentary behavior in child and adolescent cancer survivors assessed using wrist accelerometry: a cluster analysis approach. Health Informatics J. 2023 Oct-Dec;29(4):14604582231212525. PMID: 37903362. doi: 10.1177/14604582231212525.

103. Haapala EA, Rantalainen T, Hesketh KD, Rodda CP, Duckham RL. Accelerometer-based osteogenic indices, moderate-to-vigorous and vigorous physical activity, and bone traits in adolescents. J Musculoskelet Neuronal Interact. 2022 Dec;22(4):514–523. PMID:36458389.

104. Hachenberger J, Teuber Z, Li YM, Abkai L, Wild E, Lemola S. Investigating associations between physical activity, stress experience, and affective wellbeing during an examination period using experience sampling and accelerometry. Sci Rep. 2023 May;13(1):8808. PMID: 37258597.doi: 10.1038/s41598-023-35987-8.

105. Hager ER, Gormley CE, Latta LW, Treuth MS, Caulfield LE, Black MM. Toddler physical activity study: laboratory and community studies to evaluate accelerometer validity and correlates. BMC Public Health. 2016 Sep;16(1):936. PMID: 27600404. doi: 10.1186/s12889-016-3569-9.

106. Hagströmer M, Kwak L, Oja P, Sjöström M. A 6 year longitudinal study of accelerometer-measured physical activity and sedentary time in Swedish adults. J Sci Med Sport. 2015 Sep;18(5):553–557. PMID: 25277849. doi: 10.1016/j.jsams.2014.07.012.

107. Hajna S, Brage S, Dalton A, Griffin SJ, Jones AP, Khaw KT, et al. Cross-sectional and prospective associations between active living environments and accelerometer-assessed physical activity in the EPIC-Norfolk cohort. Health Place. 2021 Jan;67:102490. PMID: 33321456. doi: 10.1016/j.healthplace.2020.102490.

108. Hale JL, Knell G, Swartz MD, Shiroma EJ, Ellis T, Lee IM, et al. Association of Parkinson’s disease status with accelerometer-derived physical activity and sedentary behavior in older women: the women’s health study (WHS). Prev Med Rep. 2023 Oct;35:102361. PMID: 37584064. doi: 10.1016/j.pmedr.2023.102361.

109. Halloway S, Arfanakis K, Wilbur J, Schoeny ME, Pressler SJ. Accelerometer physical activity is associated with greater gray matter volumes in older adults without dementia or mild cognitive impairment. J Gerontol B Psychol Sci Soc Sci. 2019 Sep;74(7):1142–1151. PMID: 29432610. doi: 10.1093/geronb/gby010.

110. Halonen JI, Pulakka A, Pentti J, Kallio M, Koskela S, Kivimäki M, et al. Cross-sectional associations of neighbourhood socioeconomic disadvantage and greenness with accelerometer-measured leisure-time physical activity in a cohort of ageing workers. BMJ Open. 2020 Aug;10(8):e038673. PMID: 32801206. doi: 10.1136/bmjopen-2020-038673.

111. Hamasaki H, Noda M, Moriyama S, Yoshikawa R, Katsuyama H, Sako A, et al. Daily physical activity assessed by a triaxial accelerometer is beneficially associated with waist circumference, serum triglycerides, and insulin resistance in Japanese patients with prediabetes or untreated early type 2 diabetes. J Diabetes Res. 2015;2015:526201. PMID: 26064983. doi: 10.1155/2015/526201.

112. Hansen BH, Kolle E, Dyrstad SM, Holme I, Anderssen SA. Accelerometer-determined physical activity in adults and older people. Med Sci Sports Exerc. 2012 Feb;44(2):266–272. PMID: 21796052. doi: 10.1249/MSS.0b013e31822cb354.

113. Hazizi AS, Aina MB, Mohd NM, Zaitun Y, Hamid JJ, Tabata I. Accelerometer-determined physical activity level among government employees in Penang, Malaysia. Malays J Nutr. 2012 Apr;18(1):57-66. PMID: 23713230.

114. Healy GN, Winkler EAH, Brakenridge CL, Reeves MM, Eakin EG. Accelerometer-derived sedentary and physical activity time in overweight/obese adults with type 2 diabetes: cross-sectional associations with cardiometabolic biomarkers. PLoS One. 2015 Mar;10(3):e0119140. PMID: 25775249. doi: 10.1371/journal.pone.0119140.

115. Heitmann KA, Løchen ML, Hopstock LA, Stylidis M, Welde B, Schirmer H, et al. Cross-sectional associations between accelerometry-measured physical activity, left atrial size, and indices of left ventricular diastolic dysfunction: the Tromsø study. Prev Med Rep. 2021 Mar;21:101290. PMID: 33425668. doi: 10.1016/j.pmedr.2020.101290.

116. Helgadóttir B, Forsell Y, Ekblom Ö. Physical activity patterns of people affected by depressive and anxiety disorders as measured by accelerometers: a cross-sectional study. PLoS One. 2015 Jan;10(1):e0115894. PMID: 25585123. doi: 10.1371/journal.pone.0115894.

117. Hesketh KR, Evenson KR, Stroo M, Clancy SM, Østbye T, Benjamin-Neelon SE. Physical activity and sedentary behavior during pregnancy and postpartum, measured using hip and wrist-worn accelerometers. Prev Med Rep. 2018 Jun;10:337–345. PMID: 29868389. doi: 10.1016/j.pmedr.2018.04.012.

118. Ho EC, Hawkley L, Dale W, Waite L, Huisingh-Scheetz M. Social capital predicts accelerometry-measured physical activity among older adults in the U.S.: a cross-sectional study in the national social life, health, and aging project. BMC Public Health. 2018 Jun;18(1):804. PMID: 29945588. doi: 10.1186/s12889-018-5664-6.

119. Hooker SA, Masters KS. Purpose in life is associated with physical activity measured by accelerometer. J Health Psychol. 2016 Jun;21(6):962–971. PMID: 25104777. doi: 10.1177/1359105314542822.

120. Hooker SP, Diaz KM, Blair SN, Colabianchi N, Hutto B, McDonnell MN, et al. Association of accelerometer-measured sedentary time and physical activity with risk of stroke among US adults. JAMA Netw Open. 2022 Jun;5(6):e2215385. PMID: 35657625. doi: 10.1001/jamanetworkopen.2022.15385.

121. Hsiao C, Wen CJ, Yen HY, Hsueh MC, Liao Y. Association between accelerometer-measured light-intensity physical activity and cognitive function in older adults. J Nutr Health Aging. 2022 Mar;26(3):230–235. PMID: 35297464. doi: 10.1007/s12603-022-1749-0.

122. Huang W, Hao L, Wu X, Yu X, Cui E, Leroux A. Gender difference in “second-shift” physical activity: New insights from analyzing accelerometry data in a nationally representative sample. SSM Popul Health. 2023 Dec;24:101536. PMID: 37927817. doi: 10.1016/j.ssmph.2023.101536.

123. Hughes RL, Pindus DM, Khan NA, Burd NA, Holscher HD. Associations between accelerometer-measured physical activity and decal microbiota in adults with overweight and obesity. Med Sci Sport Exerc. 2023 Apr;55(4):680–689. PMID: 36728974. doi: 10.1249/MSS.0000000000003096.

124. Huisingh-Scheetz M, Wroblewski K, Kocherginsky M, Huang E, Dale W, Waite L, et al. The relationship between physical activity and frailty among U.S. older adults based on hourly accelerometry data. J Gerontol A Biol Sci Med Sci. 2018 Apr;73(5):622–629. PMID: 29106478. doi: 10.1093/gerona/glx208.

125. Hyde ET, LaCroix AZ, Evenson KR, Howard AG, Anuskiewicz B, Di C, et al. Accelerometer‐measured physical activity and postmenopausal breast cancer incidence in the women’s health accelerometry collaboration. Cancer. 2023 May;129(10):1579–1590. PMID: 36812131. doi: 10.1002/cncr.34699.

126. Hylkema TH, Brouwer S, Kooijman CM, De Vries AJ, Breukelman F, Dekker H, et al. Accelerometer measured sedentary and physical activity behaviors of working patients after total knee arthroplasty, and their compensation between occupational and leisure time. J Occup Rehabil. 2021 Jun;31(2):350–359. PMID: 32946009. doi: 10.1007/s10926-020-09924-9.

127. Ikeda E, Guagliano JM, Atkin AJ, Sherar LB, Ekelund U, Hansen B, et al. Cross-sectional and longitudinal associations of active travel, organised sport and physical education with accelerometer-assessed moderate-to-vigorous physical activity in young people: the international children’s accelerometry database. Int J Behav Nutr Phys Act. 2022 Apr;19(1):41. PMID: 35366914. doi: 10.1186/s12966-022-01282-4.

128. Ishikawa-Takata K, Kaneko K, Koizumi K, Ito C. Comparison of physical activity energy expenditure in Japanese adolescents assessed by EW4800P triaxial accelerometry and the doubly labelled water method. Br J Nutr. 2013 Oct;110(7):1347–1355. PMID: 23544366. doi: 10.1017/S0007114513000603.

129. Izawa KP, Watanabe S, Hiraki K, Morio Y, Kasahara Y, Takeichi N, et al. Determination of the effectiveness of accelerometer use in the promotion of physical activity in cardiac patients: a randomized controlled trial. Arch Phys Med Rehabil. 2012 Jul;93(11):1896–1902. PMID: 22750166. doi: 10.1016/j.apmr.2012.06.015.

130. Jaeschke L, Steinbrecher A, Boeing H, Gastell S, Ahrens W, Berger K, et al. Factors associated with habitual time spent in different physical activity intensities using multiday accelerometry. Sci Rep. 2020 Jan;10(1):774. PMID: 31964962. doi: 10.1038/s41598-020-57648-w.

131. Jago R, Salway R, House D, Walker R, Emm-Collison L, Sansum K, et al. Short and medium-term effects of the COVID-19 lockdowns on child and parent accelerometer-measured physical activity and sedentary time: a natural experiment. Int J Behav Nutr Phys Act. 2023 Apr 27;20(1):42. PMID: 37101270. doi: 10.1186/s12966-023-01441-1.

132. Jain RK, Vokes T. Physical activity as measured by accelerometer in NHANES 2005–2006 is associated with better bone density and trabecular bone score in older adults. Arch Osteoporos. 2019 Mar;14(1):29. PMID: 30826896. doi: 10.1007/s11657-019-0583-4.

133. Janney CA, Fagiolini A, Swartz HA, Jakicic JM, Holleman RG, Richardson CR. Are adults with bipolar disorder active? objectively measured physical activity and sedentary behavior using accelerometry. J Affect Disord. 2014 Jan;152–154:498–504. PMID: 24095103. doi: 10.1016/j.jad.2013.09.009.

134. Jin X, Chen Y, Feng H, Zhou M, Chan JWY, Liu Y, et al. Association of accelerometer-measured sleep duration and different intensities of physical activity with incident type 2 diabetes in a population-based cohort study. J Sport Health Sci. 2024 Mar;13(2):222–232. PMID: 36871624. doi: 10.1016/j.jshs.2023.03.001.

135. Jinhyuk Kim, Nakamura T, Kikuchi H, Yoshiuchi K, Yamamoto Y. Co-variation of depressive mood and spontaneous physical activity evaluated by ecological momentary assessment in major depressive disorder. 2014 36th Annual International Conference of the IEEE Engineering in Medicine and Biology Society; 2014 Aug 26-

136. Júdice PB, Hetherington-Rauth M, Northstone K, Andersen LB, Wedderkopp N, Ekelund U, et al. Changes in physical activity and sedentary patterns on cardiometabolic outcomes in the transition to adolescence: international children’s accelerometry database 2.0. J Pediatr. 2020 Oct;225:166-173.e1. PMID: 32553870. doi: 10.1016/j.jpeds.2020.06.018.

137. Jung ME, Locke SR, Bourne JE, Beauchamp MR, Lee T, Singer J, et al. Cardiorespiratory fitness and accelerometer-determined physical activity following one year of free-living high-intensity interval training and moderate-intensity continuous training: a randomized trial. Int J Behav Nutr Phys Act. 2020 Feb;17(1):25. PMID: 32102667. doi: 10.1186/s12966-020-00933-8.

138. Junno JA, Keisu A, Niemelä M, Modarress Julin M, Korpelainen R, Jämsä T, et al. Accelerometer-measured physical activity is associated with knee breadth in middle-aged Finns – a population-based study. BMC Musculoskelet Disord. 2022 May;23(1):517. PMID: 35642051. doi: 10.1186/s12891-022-05475-7.

139. Junttila HE, Vaaramo MM, Huikari SM, Kari JT, Leinonen A, Farrahi V, et al. Association of accelerometer‐measured physical activity and midlife income: a northern Finland birth cohort 1966 study. Scand Med Sci Sports. 2023 Sep;33(9):1765–1778. PMID: 37272147. doi: 10.1111/sms.14421.

140. Jussila AM, Husu P, Vähä-Ypyä H, Tokola K, Kokko S, Sievänen H, et al. Accelerometer-measured physical activity levels and patterns vary in an age- and sex-dependent fashion among Finnish children and adolescents. Int J Environ Res Public Health. 2022 Jun;19(11):6950. PMID: 35682533. doi: 10.3390/ijerph19116950.

141. Kanai M, Izawa KP, Kobayashi M, Onishi A, Kubo H, Nozoe M, et al. Effect of accelerometer-based feedback on physical activity in hospitalized patients with ischemic stroke: a randomized controlled trial. Clin Rehabil. 2018 Aug;32(8):1047–1056. PMID: 29400070. doi: 10.1177/0269215518755841.

142. Kantomaa MT, Tikanmäki M, Kankaanpää A, Vääräsmäki M, Sipola-Leppänen M, Ekelund U, et al. Accelerometer-measured physical activity and sedentary time differ according to education level in young adults. PLoS One. 2016 Jul;11(7):e0158902. PMID: 27403958. doi: 10.1371/journal.pone.0158902.

143. Keller JL, Tian F, Fitzgerald KC, Mische L, Ritter J, Costello MG, et al. Using real-world accelerometry-derived diurnal patterns of physical activity to evaluate disability in multiple sclerosis. J Rehabil Assist Technol Eng. 2022 Jan;9:205566832110673. PMID: 35070348. doi: 10.1177/20556683211067362.

144. Ketels M, Rasmussen CL, Korshøj M, Gupta N, De Bacquer D, Holtermann A, et al. The relation between domain-specific physical behaviour and cardiorespiratory fitness: a cross-sectional compositional data analysis on the physical activity health paradox using accelerometer-assessed data. Int J Environ Res Public Health. 2020 Oct;17(21):7929. PMID: 33137943. doi: 10.3390/ijerph17217929.

145. Khurshid S, Al-Alusi MA, Churchill TW, Guseh JS, Ellinor PT. Accelerometer-derived “weekend warrior” physical activity and incident cardiovascular disease. JAMA. 2023 Jul;330(3):247. PMID: 37462704. doi: 10.1001/jama.2023.10875.

146. Khurshid S, Weng LC, Al-Alusi MA, Halford JL, Haimovich JS, Benjamin EJ, et al. Accelerometer-derived physical activity and risk of atrial fibrillation. Euro Heart J. 2021 Jul;42(25):2472–2483. PMID: 34037209. doi: 10.1093/eurheartj/ehab250.

147. Khurshid S, Weng LC, Nauffal V, Pirruccello JP, Venn RA, Al-Alusi MA, et al. Wearable accelerometer-derived physical activity and incident disease. NPJ Digit Med. 2022 Sep;5(1):131. PMID: 36056190. doi: 10.1038/s41746-022-00676-9.

148. Kijima T, Akai K, Amagasa S, Inoue S, Yamagata S, Ishibashi Y, et al. Accelerometer-measured physical activity and posture among older adults in assisted-living residences. SAGE Open Med. 2024 Jan;12:20503121231220798. PMID: 38186563. doi: 10.1177/20503121231220798.

149. Kim Y, Wijndaele K, Sharp SJ, Strain T, Pearce M, White T, et al. Specific physical activities, sedentary behaviours and sleep as long-term predictors of accelerometer-measured physical activity in 91,648 adults: a prospective cohort study. Int J Behav Nutr Phys Act. 2019 May;16(1):41. PMID: 31064403. doi: 10.1186/s12966-019-0802-9.

150. Kinnunen TI, Tennant PW, McParlin C, Poston L, Robson SC, Bell R. Agreement between pedometer and accelerometer in measuring physical activity in overweight and obese pregnant women. BMC Public Health. 2011 Jun;11(1):501. PMID: 21703033. doi: 10.1186/1471-2458-11-501.

151. Kong S, Park HY, Kang D, Lee JK, Lee G, Kwon OJ, et al. Seasonal variation in physical activity among preoperative patients with lung cancer determined using a wearable Device. J Clin Med. 2020 Jan;9(2):349. PMID: 32012720. doi: 10.3390/jcm9020349.

152. Kretzschmar M, Lin W, Nardo L, Joseph GB, Dunlop DD, Heilmeier U, et al. Association of physical activity measured by accelerometer, knee joint abnormalities, and cartilage T2 measurements obtained from 3T magnetic resonance imaging: data from the osteoarthritis initiative. Arthritis Care Res (Hoboken). 2015 Sep;67(9):1272–1280. PMID: 25777255. doi: 10.1002/acr.22586.

153. Kruisdijk F, Deenik J, Tenback D, Tak E, Beekman AJ, Van Harten P, et al. Accelerometer-measured sedentary behaviour and physical activity of inpatients with severe mental illness. Psychiatry Res. 2017 Aug;254:67–74. PMID: 28456024. doi: 10.1016/j.psychres.2017.04.035.

154. Kulinski JP, Khera A, Ayers CR, Das SR, De Lemos JA, Blair SN, et al. Association between cardiorespiratory fitness and accelerometer-derived physical activity and sedentary time in the general population. Mayo Clin Proc. 2014 Aug;89(8):1063–1071. PMID: 25012770. doi: 10.1016/j.mayocp.2014.04.019.

155. Kuritz A, Mall C, Schnitzius M, Mess F. Physical activity and sedentary behavior of children in afterschool programs: an accelerometer-based analysis in full-day and half-day elementary schools in Germany. Front Public Health. 2020 Sep;8:463. PMID: 32984249. doi: 10.3389/fpubh.2020.00463.

156. Kwon S, Andersen LB, Grøntved A, Kolle E, Cardon G, Davey R, et al. A closer look at the relationship among accelerometer-based physical activity metrics: ICAD pooled data. Int J Behav Nutr Phys Act. 2019 Apr;16(1):40. PMID: 31036032. doi: 10.1186/s12966-019-0801-x.

157. Kwon S, Janz KF, International Children’s Accelerometry Database (ICAD) Collaborators. Tracking of accelerometry-measured physical activity during childhood: ICAD pooled analysis. Int J Behav Nutr Phys Act. 2012 Jun;9:68. PMID: 22676230. doi: 10.1186/1479-5868-9-68.

158. LaCroix AZ, Bellettiere J, Rillamas-Sun E, Di C, Evenson KR, Lewis CE, et al. Association of light physical activity measured by accelerometry and incidence of coronary heart disease and cardiovascular disease in older women. JAMA Netw Open. 2019 Mar;2(3):e190419. PMID: 30874775. doi: 10.1001/jamanetworkopen.2019.0419.

159. Ladlow P, Nightingale TE, McGuigan MP, Bennett AN, Phillip R, Bilzon JLJ. Impact of anatomical placement of an accelerometer on prediction of physical activity energy expenditure in lower-limb amputees. PLoS One. 2017 Oct;12(10):e0185731. PMID: 28982199. doi: 10.1371/journal.pone.0185731.

160. LaMonte MJ, Buchner DM, Rillamas‐Sun E, Di C, Evenson KR, Bellettiere J, et al. Accelerometer‐measured physical activity and mortality in women aged 63 to 99. J Am Geriatr Soc. 2018 May;66(5):886–894. PMID: 29143320. doi: 10.1111/jgs.15201.

161. LaMonte MJ, LaCroix AZ, Nguyen S, Evenson KR, Di C, Stefanick ML, et al. Accelerometer-measured physical activity, sedentary time, and heart failure risk in women aged 63 to 99 years. JAMA Cardiol. 2024 Feb;e235692 PMID: 38381446. doi: 10.1001/jamacardio.2023.5692.

162. LaMonte MJ, Lewis CE, Buchner DM, Evenson KR, Rillamas‐Sun E, Di C, et al. Both light intensity and moderate‐to‐vigorous physical activity measured by accelerometry are favorably associated with cardiometabolic risk factors in older women: the objective physical activity and cardiovascular health (OPACH) study. J Am Heart Assoc. 2017 Oct;6(10):e007064. PMID: 29042429. doi: 10.1161/JAHA.117.007064.

163. Länsitie M, Kangas M, Jokelainen J, Venojärvi M, Vaaramo E, Härkönen P, et al. Association between accelerometer-measured physical activity, glucose metabolism, and waist circumference in older adults. Diabetes Res Clin Pract. 2021 Aug;178:108937. PMID: 34217770. doi: 10.1016/j.diabres.2021.108937.

164. Länsitie M, Kangas M, Jokelainen J, Venojärvi M, Timonen M, Keinänen-Kiukaanniemi S, et al. Cardiovascular disease risk and all-cause mortality associated with accelerometer-measured physical activity and sedentary time ‒ a prospective population-based study in older adults. BMC Geriatr. 2022 Sep;22(1):729. PMID: 36064345. doi: 10.1186/s12877-022-03414-8.

165. Lawman HG, Horn MLV, Wilson DK, Pate RR. A multilevel approach to examining time-specific effects in accelerometer-assessed physical activity. J Sci Med Sport. 2015 Nov;18(6):667–672. PMID: 25245427. doi: 10.1016/j.jsams.2014.09.003.

166. Le Cornu Q, Chen M, Van Hees V, Léger D, Fayosse A, Yerramalla MS, et al. Association of physical activity, sedentary behaviour, and daylight exposure with sleep in an ageing population: findings from the Whitehall accelerometer sub-study. Int J Behav Nutr Phys Act. 2022 Dec;19(1):144. PMID: 36494722. doi: 10.1186/s12966-022-01391-0.

167. Lee IM, Shiroma EJ, Evenson KR, Kamada M, LaCroix AZ, Buring JE. Accelerometer-measured physical activity and sedentary behavior in relation to all-cause mortality: the women’s health study. Circulation. 2018 Jan;137(2):203–205. PMID: 29109088. doi: 10.1161/CIRCULATIONAHA.117.031300.

168. Lee J, Walker ME, Gabriel KP, Vasan RS, Xanthakis V. Associations of accelerometer-measured physical activity and sedentary time with chronic kidney disease: The Framingham Heart Study. PLoS One. 2020 Jun;15(6):e0234825. PMID: 32542048. doi: 10.1371/journal.pone.0234825.

169. Lee PH. Examining non-linear associations between accelerometer-neasured physical activity, sedentary behavior, and all-cause mortality using segmented cox regression. Front Physiol. 2016 Jun;7:272. PMID: 27445859. doi: 10.3389/fphys.2016.00272.

170. Lee SY, Kim SC, Gim JA, Park SJ, Seo SH, Kim SJ, et al. Accelerometer-derived physical activity analysis of elderly osteoarthritis patients. Musculoskelet Sci Practi. 2023 Aug;66:102808. PMID: 37352763. doi: 10.1016/j.msksp.2023.102808.

171. Leininger B, Schulz C, Gao Z, Bronfort G, Evans R, Pope Z, et al. Accelerometer-determined physical activity and clinical low back pain measures in adolescents with chronic or subacute recurrent low back pain. J Orthop Sports Phys Ther. 2017 Oct;47(10):769–774. PMID: 28898136. doi: 10.2519/jospt.2017.7345.

172. Lemberg GM, Riso EM, Fjørtoft I, Kjønniksen L, Kull M, Mäestu E. School children’s Physical activity and preferred activities during outdoor recess in Estonia: using accelerometers, recess observation, and schoolyard mapping. Children. 2023 Apr;10(4):702. PMID: 37189951. doi: 10.3390/children10040702.

173. Li T, Zong G, Peng P, Wang S, Cheng B. Accelerometer-measured physical activity and sample-based frailty in older women: does pattern really matter? Front Public Health. 2024 Jan;11:1304279. PMID: 38332942. doi: 10.3389/fpubh.2023.1304279.

174. Li X, Kearney PM, Keane E, Harrington JM, Fitzgerald AP. levels and sociodemographic correlates of accelerometer-based physical activity in Irish children: a cross-sectional study. J Epidemiol Community Health. 2017 Jun;71(6):521–527. PMID: 28130391. doi: 10.1136/jech-2016-207691

175. Liang YY, Feng H, Chen Y, Jin X, Xue H, Zhou M, Ma H, Ai S, Wing YK, Geng Q, Zhang J. Joint association of physical activity and sleep duration with risk of all-cause and cause-specific mortality: a population-based cohort study using accelerometry. Eur J Prev Cardiol. 2023 Jul;30(9):832-843. PMID: 36990109. doi: 10.1093/eurjpc/zwad060.

176. Lin CY, Lin KP, Hsueh MC, Liao Y. Associations of accelerometer-measured sedentary behavior and physical activity with sleep in older adults. J Formos Med Assoc. 2023 Aug;S0929664623002966. PMID: 37586972. doi: 10.1016/j.jfma.2023.08.001.

177. Lin H, Hartley P, Forsyth F, Pilling M, Hobbs FDR, Taylor CJ, et al. Clinical and demographic correlates of accelerometer-measured physical activity in participants enrolled in the OPTIMISE HFpEF study. Eur J Cardiovasc Nurs 2022 Jan;21(1):67–75. PMID: 33837414. doi: 10.1093/eurjcn/zvab028.

178. Liu M, Gan X, Ye Z, Zhang Y, He P, Zhou C, et al. Association of accelerometer-measured physical activity intensity, sedentary time, and exercise time with incident Parkinson’s disease. NPJ Digit Med 2023 Nov;6(1):224. PMID: 38017114. doi: 10.1038/s41746-023-00969-7.

179. Liu M, Zhang Y, Zhang Y, He P, Zhou C, Ye Z, et al. Association of accelerometer-measured physical activity and its change with progression to chronic kidney disease in adults with type 2 diabetes and overweight/obesity. Br J Sports Med. 2024 Mar;58(6):313–319. PMID: 38320851. doi: 10.1136/bjsports-2023-107564.

180. Long MT, Pedley A, Massaro JM, Hoffmann U, Esliger DW, Vasan RS, et al. Hepatic steatosis is associated with lower levels of physical activity measured via accelerometry. Obesity. 2015 Jun;23(6):1259–1266. PMID: 25959049. doi: 10.1002/oby.21058.

181. Loprinzi PD. Accelerometer-determined physical activity and mortality in a national prospective cohort study of adults at high risk of a first atherosclerotic cardiovascular disease event. Int J Cardiol. 2016 Jan;202:417–418. PMID: 26432494. doi: 10.1016/j.ijcard.2015.09.061.

182. Loprinzi PD. Accelerometer-determined physical activity and all-cause mortality in a national prospective cohort study of hypertensive adults. J Hypertens. 2016 May;34(5):848–852. PMID: 26828782. doi: 10.1097/HJH.0000000000000869.

183. Loprinzi PD, Addoh O. Accelerometer-determined physical activity and all-cause mortality in a national prospective cohort study of adults post-acute stroke. Am J Health Promot 2018 Jan;32(1):24–27. PMID: 28718295. doi: 10.1177/0890117117720061.

184. Loprinzi PD, Frith E. Accelerometer-assessed physical activity and school absenteeism due to illness or injury among children and adolescents: NHANES 2003 to 2006. Am J Health Promot. 2018 Mar;32(3):571–577. PMID: 29431510. doi: 10.1177/0890117116684241.

185. Loprinzi PD, Gilham B, Cardinal BJ. Association between accelerometer-assessed physical activity and objectively measured hearing sensitivity among U.S. adults with diabetes. Res Q Exerc Sport. 2014 Sep;85(3):390–397. PMID: 25141090. doi: 10.1080/02701367.2014.930404.

186. Loprinzi PD, Joyner C. Accelerometer-determined physical activity and mortality in a national prospective cohort study: Considerations by visual acuity. Prev Med. 2016 Jun;87:18–21. PMID: 26861750. doi: 10.1016/j.ypmed.2016.02.005.

187. Loprinzi PD, Lee H, Gilham B, Cardinal BJ. Association between accelerometer-assessed physical activity and tinnitus, NHANES 2005–2006. Res Q Exerc Sport. 2013 Jun;84(2):177–185. PMID: 23930543. doi: 10.1080/02701367.2013.784840.

188. Loprinzi PD, Sheffield J, Tyo BM, Fittipaldi-Wert J. Accelerometer-determined physical activity, mobility disability, and health. Disabil Health J. 2014 Oct;7(4):419–425. PMID: 25224982. doi: 10.1016/j.dhjo.2014.05.005.

189. Loprinzi PD, Smit E, Lin FR, Gilham B, Ramulu PY. Accelerometer-assessed physical activity and objectively determined dual sensory impairment in US adults. Mayo Clinic Proc. 2013 Jul;88(7):690–696. PMID: 23751983. doi: 10.1016/j.mayocp.2013.04.008.

190. Loprinzi P, Smit E, Lee H, Crespo C, Andersen R, Blair S. The “Fit but Fat” paradigm addressed using accelerometer-determined physical activity data. N Am J Med Sci. 2014 Jul;6(7):295. PMID: 25077076. doi: 10.4103/1947-2714.136901.

191. Loyen A, Clarke-Cornwell AM, Anderssen SA, Hagströmer M, Sardinha LB, Sundquist K, et al. Sedentary time and physical activity surveillance through accelerometer pooling in four European countries. Sports Med. 2017 Jul;47(7):1421–1435. PMID: 27943147. doi: 10.1007/s40279-016-0658-y.

192. Lund K, Larsen MD, Knudsen T, Kjeldsen J, Nielsen RG, Brage S, et al. Physical activity measured by accelerometry in paediatric and young adult patients with inflammatory bowel disease. BMC Gastroenterol. 2022 Jun;22(1):290. PMID: 35672666. doi: 10.1186/s12876-022-02358-y.

193. Lund Rasmussen C, Palarea-Albaladejo J, Korshøj M, Gupta N, Nabe-Nielsen K, Holtermann A, et al. Is high aerobic workload at work associated with leisure time physical activity and sedentary behaviour among blue-collar workers? a compositional data analysis based on accelerometer data. PLoS One. 2019 Jun;14(6):e0217024. PMID: 31170169. doi: 31170169. 10.1371/journal.pone.0217024.

194. Luo M, Yu C, Del Pozo Cruz B, Chen L, Ding D. Accelerometer-measured intensity-specific physical activity, genetic risk and incident type 2 diabetes: a prospective cohort study. Br J Sports Med. 2023 Oct;57(19):1257–1264. PMID: 37277158. doi: 10.1136/bjsports-2022-106653.

195. Ma J, Kim H, Kim J. Isotemporal substitution analysis of accelerometer-derived sedentary behavior and physical activity on cardiometabolic health in Korean adults: a population-based cross-sectional study. Int J Environ Res Public Health. 2021 Oct;18(21):11102. PMID: 34769622. doi: 10.3390/ijerph182111102.

196. Machado-Rodrigues AM, Rodrigues D, Gama A, Nogueira H, Silva M-RG, Mascarenhas LP, et al. Tri-axial accelerometer-assessed physical activity and its association with weight status in a sample of elementary-school children. Obes Res Clin Pract. 2023 May;17(3):192–197. PMID: 37087316. doi: 10.1016/j.orcp.2023.04.003.

197. Madimenos FC, Snodgrass JJ, Blackwell AD, Liebert MA, Sugiyama LS. Physical activity in an indigenous Ecuadorian forager‐horticulturalist population as measured using accelerometry. Am J Hum Biol. 2011 Jul;23(4):488–497. PMID: 21538650. doi: 10.1002/ajhb.21163.

198. Maes I, Van Dyck D, Van Cauwenberg J, Mertens L. Age-related differences in the associations of physical environmental factors and psychosocial factors with accelerometer-assessed physical activity. Health Place. 2021 Jan;67:102492. PMID: 33316601. doi: 10.1016/j.healthplace.2020.102492.

199. Malek ME, Norman Å, Elinder LS, Patterson E, Nyberg G. Relationships between physical activity parenting practices and children’s activity measured by accelerometry with children’s activity style as a moderator—a cross sectional study. Children. 2022 Feb;9(2):248. PMID: 35204968. doi: 10.3390/children9020248.

200. Mañas A, Del Pozo-Cruz B, Guadalupe-Grau A, Marín-Puyalto J, Alfaro-Acha A, Rodríguez-Mañas L, et al. Reallocating accelerometer-assessed sedentary time to light or moderate- to vigorous-intensity physical activity reduces frailty levels in older adults: an isotemporal substitution spproach in the TSHA dtudy. J Am Med Dir Assoc. 2018 Feb;19(2):185.e1-185.e6. PMID: 29269096. doi: 10.1016/j.jamda.2017.11.003.

201. Mañas A, Pozo-Cruz BD, Rodríguez-Gómez I, Losa-Reyna J, Rodríguez-Mañas L, García-García FJ, et al. Can physical activity offset the detrimental consequences of sedentary time on frailty? a moderation analysis in 749 older adults measured with accelerometers. J Am Med Dir Assoc. 2019 May;20(5):634-638.e1. PMID: 30738823. doi: 10.1016/j.jamda.2018.12.012.

202. Manglani HR, Phansikar M, Duraney EJ, McKenna MR, Canter R, Nicholas JA, et al. Accelerometry measures of physical activity and sedentary behavior: Associations with cognitive functioning in MS. Mult Scler Relat Disord. 2023 Nov;79:104963. PMID: 37690438. doi: 10.1016/j.msard.2023.104963.

203. Manns P, Ezeugwu V, Armijo‐Olivo S, Vallance J, Healy GN. Accelerometer‐derived pattern of sedentary and physical activity time in persons with mobility disability: national health and nutrition examination survey 2003 to 2006. J Am Geriatr Soc. 2015 Jul;63(7):1314–1323. PMID: 26173621. doi: 10.1111/jgs.13490.

204. Maslow AL, Colabianchi N. Youth physical activity resource use and activity measured by accelerometry. Am J Health Behav. 2011 Mar-Apr;35(2):219-227. PMID: 21204684. doi: 10.5993/ajhb.35.2.9.

205. Matabuena M, Félix P, Hammouri ZAA, Mota J, Del Pozo Cruz B. Physical activity phenotypes and mortality in older adults: a novel distributional data analysis of accelerometry in the NHANES. Aging Clin Exp Res. 2022 Dec;34(12):3107–3114. PMID: 36183279. doi: 10.1007/s40520-022-02260-3.

206. Matsuzawa R, Matsunaga A, Kutsuna T, Ishii A, Abe Y, Yoneki K, et al. Association of habitual physical activity measured by an accelerometer with high-density lipoprotein cholesterol levels in maintenance Hemodialysis patients. Sci World J. 2013 Dec;2013:780783. PMID: 24453904. doi: 10.1155/2013/780783.

207. Matthews CE, Keadle SK, Troiano RP, Kahle L, Koster A, Brychta R, et al. Accelerometer-measured dose-response for physical activity, sedentary time, and mortality in US adults. Ame J Clini Nutr. 2016 Nov;104(5):1424–1432. PMID: 27707702. doi: 10.3945/ajcn.116.135129.

208. Maylor BD, Edwardson CL, Clarke-Cornwell AM, Davies MJ, Dawkins NP, Dunstan DW, et al. Physical activity assessed by wrist and thigh worn accelerometry and associations with cardiometabolic health. Sensors. 2023 Aug;23(17):7353. PMID: 37687813. doi: 10.3390/s23177353.

209. McDonald L, Oguz M, Carroll R, Thakkar P, Yang F, Dhalwani N, et al. Comparison of accelerometer-derived physical activity levels between individuals with and without cancer: a UK Biobank study. Future Oncol. 2019 Nov;15(33):3763–3774. PMID: 31637942. doi: 10.2217/fon-2019-0443.

210. McLellan G, Arthur R, Donnelly S, Buchan DS. Segmented sedentary time and physical activity patterns throughout the week from wrist-worn ActiGraph GT3X+ accelerometers among children 7–12 years old. J Sport Health Sci. 2020 Mar;9(2):179–188. PMID: 32099726. doi: 10.1016/j.jshs.2019.02.005.

211. Melin M, Hagerman I, Gonon A, Gustafsson T, Rullman E. Variability in physical activity assessed with accelerometer is an independent predictor of mortality in CHF patients. PLoS One. 2016 Apr;11(4):e0153036. PMID: 27054323. doi: 10.1371/journal.pone.0153036.

212. Memari AH, Ghaheri B, Ziaee V, Kordi R, Hafizi S, Moshayedi P. Physical activity in children and adolescents with autism assessed by triaxial accelerometry. Pediatr Obes. 2013 Apr;8(2):150–158. PMID: 23042790. doi: 10.1111/j.2047-6310.2012.00101.x.

213. Micklesfield LK, Westgate K, Smith A, Kufe CN, Mendham AE, Lindsay T, et al. Physical activity behaviors of a middle-age south african cohort as determined by integrated hip and thigh accelerometry. Med Sci Sports Exerc. 2022 Sep;54(9):1493–1505. PMID: 35658390. doi: 10.1249/MSS.0000000000002940.

214. Mossavar-Rahmani Y, Lin J, Pan S, Song RJ, Xue X, Spartano NL, et al. Characterizing longitudinal change in accelerometry-based moderate-to-vigorous physical activity in the Hispanic community health study/study of latinos and the Framingham heart study. BMC Public Health. 2023 Aug;23(1):1614. PMID: 37620824. doi: 10.1186/s12889-023-16442-9.

215. Moy ML, Matthess K, Stolzmann K, Reilly J, Garshick E. Free-living physical activity in COPD: assessment with accelerometer and activity checklist. J Rehabil Res Dev. 2009;46(2):277–286. PMID:19533541.

216. Murabito JM, Pedley A, Massaro JM, Vasan RS, Esliger D, Blease SJ, et al. Moderate‐to‐vigorous physical activity with accelerometry is associated with visceral adipose tissue in adults. J Am Heart Assoc. 2015 Mar;4(3):e001379. PMID: 25736442. doi: 10.1161/JAHA.114.001379.

217. Nakabazzi B, Wachira LM, Oyeyemi AL, Ssenyonga R, Onywera VO. Parental-perceived home and neighborhood environmental correlates of accelerometer-measured physical activity among school-going children in Uganda. PLOS Glob Public Health. 2021 Dec;1(12):e0000089. PMID: 36962124. doi: 10.1371/journal.pgph.0000089.

218. Nam S, Jeon S, Ash G, Whittemore R, Vlahov D. Racial discrimination, sedentary time, and physical activity in african americans: quantitative study combining ecological momentary assessment and accelerometers. JMIR Form Res. 2021 Jun;5(6):e25687. PMID: 34096870. doi: 10.2196/25687.

219. Nawab KA, Storey BC, Staplin N, Walmsley R, Haynes R, Sutherland S, et al. Accelerometer-measured physical activity and functional behaviours among people on dialysis. Clin Kidney J. 2020 Aug;14(3):950–958. PMID: 33777379. doi: 10.1093/ckj/sfaa045.

220. Nguyen S, LaCroix AZ, Hayden KM, Di C, Palta P, Stefanick ML, et al. Accelerometer‐measured physical activity and sitting with incident mild cognitive impairment or probable dementia among older women. Alzheimers Dement. 2023 Jul;19(7):3041–3054. PMID: 36695426. doi: 10.1002/alz.12908.

221. Nordstrøm M, Hansen BH, Paus B, Kolset SO. Accelerometer-determined physical activity and walking capacity in persons with down syndrome, williams syndrome and prader–willi syndrome. Res Dev Disabil. 2013 Dec;34(12):4395–4403. PMID: 24139709. doi: 10.1016/j.ridd.2013.09.021.

222. Nørgaard M, Lomholt J, Thastum M, Herlin M, Twilt M, Herlin T. Accelerometer-assessed daily physical activity in relation to pain cognition in juvenile idiopathic arthritis. Scand J Rheumatol. 2017 Jan;46(1):22–26. PMID: 27308924. doi: 10.3109/03009742.2016.1160146.

223. Oliver M, Schluter PJ, Schofield GM, Paterson J. Factors related to accelerometer-derived physical activity in pacific children aged 6 years. Asia Pac J Public Health. 2011 Jan;23(1):44–56. PMID: 20484244. doi: 10.1177/1010539510370992.

224. Orme MW, Steiner MC, Morgan MD, Kingsnorth AP, Esliger DW, Singh SJ, et al. 24-hour accelerometry in COPD: exploring physical activity, sedentary behavior, sleep and clinical characteristics. Int J Chron Obstruct Pulmon Dis. 2019 Feb;14:419–430. PMID: 30863042. doi: 10.2147/COPD.S183029.

225. Ortlieb S, Gorzelniak L, Nowak D, Strobl R, Grill E, Thorand B, et al. Associations between multiple accelerometry-assessed physical activity parameters and selected health outcomes in elderly people – results from the KORA-age study. PLoS One. 2014 Nov;9(11):e111206. PMID: 25372399. doi: 10.1371/journal.pone.0111206.

226. Parada H, McDonald E, Bellettiere J, Evenson KR, LaMonte MJ, LaCroix AZ. Associations of accelerometer-measured physical activity and physical activity-related cancer incidence in older women: results from the WHI OPACH Study. Br J Cancer. 2020 Apr;122(9):1409–1416. PMID: 32139875. doi: 10.1038/s41416-020-0753-6.

227. Paschali AA, Kalantzi-Azizi A, Goodrick GK, Papadatou D, Balasubramanyam A. Accelerometer feedback to promote physical activity in adults with type 2 diabetes: a pilot study. Percept Mot Skills. 2005 Feb;100(1):61–68. PMID: 15773694. doi: 10.2466/pms.100.1.61-68.

228. Peter‐Marske KM, Evenson KR, Moore CC, Cuthbertson CC, Howard AG, Shiroma EJ, et al. Association of accelerometer‐measured physical activity and sedentary behavior with incident cardiovascular disease, myocardial infarction, and ischemic stroke: the women’s health study. J Am Heart Assoc. 2023 Apr;12(7):e028180. PMID: 36974744. doi: 10.1161/JAHA.122.028180.

229. Peters TM, Moore SC, Xiang YB, Yang G, Shu XO, Ekelund U, et al. Accelerometer-measured physical activity in Chinese adults. Am J Prev Med. 2010 Jun;38(6):583–591. PMID: 20494234. doi: 10.1016/j.amepre.2010.02.012.

230. Petersen TL, Brønd JC, Kristensen PL, Aadland E, Grøntved A, Jepsen R. Resemblance in accelerometer-assessed physical activity in families with children: the Lolland-Falster health study. Int J Behav Nutr Phys Act. 2020 Dec;17(1):161. PMID: 33276796. doi: 10.1186/s12966-020-01067-7.

231. Pettee Gabriel K, Karvonen-Gutierrez CA, Colvin AB, Ylitalo KR, Whitaker KM, Lange-Maia BS, et al. Associations of accelerometer-determined sedentary behavior and physical activity with physical performance outcomes by race/ethnicity in older women. Prev Med Rep. 2021 May;23:101408. PMID: 34123715. doi: 10.1016/j.pmedr.2021.101408.

232. Pettee Gabriel K, Sternfeld B, Shiroma EJ, Pérez A, Cheung J, Lee IM. Bidirectional associations of accelerometer-determined sedentary behavior and physical activity with reported time in bed: women’s health study. Sleep Health. 2017 Feb;3(1):49–55. PMID: 28346151. doi: 10.1016/j.sleh.2016.10.001.

233. Phan A, Askim T, Lydersen S, Indredavik B, Wethal T. Accelerometer-measured physical activity at 3 months as a predictor of symptoms of depression and anxiety 1 year after stroke: a multicentre prospective cohort study in central Norway. J Rehabil Med. 2023 Nov;55:jrm12309. PMID: 37970656. doi: 10.2340/jrm.v55.12309.

234. Posis AIB, Bellettiere J, Salem RM, LaMonte MJ, Manson JE, Casanova R, et al. Associations of accelerometer-measured physical activity and sedentary time with all-cause mortality by genetic predisposition for longevity. J Aging Phys Act. 2022 Aug;31(2):265–275. PMID: 36002033. doi: 10.1123/japa.2022-0067.

235. Prioreschi A, Hodkinson B, Tikly M, McVeigh JA. Changes in physical activity measured by accelerometry following initiation of DMARD therapy in rheumatoid arthritis. Rheumatology. 2014 May;53(5):923–926. PMID: 24459221. doi: 10.1093/rheumatology/ket457.

236. Pugh ME, Buchowski MS, Robbins IM, Newman JH, Hemnes AR. Physical activity limitation as measured by accelerometry in pulmonary arterial hypertension. Chest. 2012 Dec;142(6):1391–1398. PMID: 22576635. doi: 10.1378/chest.12-0150.

237. Qi G, Dutta D, Leroux A, Ray D, Muschelli J, Crainiceanu C, et al. Genome‐wide association studies of 27 accelerometry‐derived physical activity measurements identified novel loci and genetic mechanisms. Genet Epidemiol. 2022 Mar;46(2):122–138. PMID: 35043453. doi: 10.1002/gepi.22441.

238. Qiu S, Xing Z. Association between accelerometer-derived physical activity and incident cardiac arrest. Europace. 2023 Dec;25(12):euad353. PMID: 38016070. doi: 10.1093/europace/euad353.

239. Ramulu PY, Maul E, Hochberg C, Chan ES, Ferrucci L, Friedman DS. Real-world assessment of physical activity in glaucoma using an accelerometer. Ophthalmology. 2012 Jun;119(6):1159–1166. PMID: 22386950. doi: 10.1016/j.ophtha.2012.01.013.

240. Ricketts HC, Buchan DS, Steffensen F, Chaudhuri R, Baker JS, Cowan DC. Physical activity levels in asthma: relationship with disease severity, body mass index and novel accelerometer-derived metrics. J Asthma. 2023 Apr;60(4):824–834. PMID: 35876843. doi: 10.1080/02770903.2022.2102037.

241. Robertson MC, Green CE, Liao Y, Durand CP, Basen-Engquist KM. Self-efficacy and ohysical activity in overweight and obese adults participating in a worksite weight loss intervention: multistate modeling of wearable device data. Cancer Epidemiol Biomarkers Prev. 2020 Apr;29(4):769–776. PMID: 31871110. doi: 10.1158/1055-9965.EPI-19-0907.

242. Roscoe CMP, James RS, Duncan MJ. Accelerometer-based physical activity levels differ between week and weekend days in british preschool children. J Funct Morphol Kinesiol. 2019 Sep;4(3):65. PMID: 33467380. doi: 10.3390/jfmk4030065.

243. Rowlands AV, Dempsey PC, Gillies C, Kloecker DE, Razieh C, Chudasama Y, et al. Association between accelerometer-assessed physical activity and severity of COVID-19 in UK Biobank. Mayo Clin Proc Innov Qual Outcomes. 2021 Dec;5(6):997–1007. PMID: 34430796. doi: 10.1016/j.mayocpiqo.2021.08.011.

244. Rowlands AV, Henson JJ, Coull NA, Edwardson CL, Brady E, Hall A, et al. The impact of COVID‐19 restrictions on accelerometer‐assessed physical activity and sleep in individuals with type 2 diabetes. Diabet Med. 2021 Oct;38(10):e14549. PMID: 33650112. doi: 10.1111/dme.14549.

245. Rowlands AV, Van Hees VT, Dawkins NP, Maylor BD, Plekhanova T, Henson J, et al. Accelerometer-assessed physical activity in people with type 2 diabetes: accounting for sleep when determining associations with markers of health. Sensors. 2023 Jun;23(12):5382. PMID: 37420551. doi: 10.3390/s23125382.

246. Salerno EA, Saint-Maurice PF, Wan F, Peterson LL, Park Y, Cao Y, et al. Prospective associations between accelerometry-derived physical activity and sedentary behaviors and mortality among cancer survivors. JNCI Cancer Spectr. 2023 Mar;7(2):pkad007. PMID: 36786414. doi: 10.1093/jncics/pkad007.

247. Salvo D, Torres C, Villa U, Rivera JA, Sarmiento OL, Reis RS, et al. Accelerometer-based physical activity levels among Mexican adults and their relation with sociodemographic characteristics and BMI: a cross-sectional study. Int J Behav Nutr Phys Act. 2015 Jun;12(1):79. PMID: 26088430. doi: 10.1186/s12966-015-0243-z,

248. Salway R, Foster C, De Vocht F, Tibbitts B, Emm-Collison L, House D, et al. Accelerometer-measured physical activity and sedentary time among children and their parents in the UK before and after COVID-19 lockdowns: a natural experiment. Int J Behav Nutr Phys Act. 2022 May;19(1):51. PMID: 35570265. doi: 10.1186/s12966-022-01290-4.

249. Sandroff BM, Motl RW, Kam JP, Pula JH. Accelerometer measured physical activity and the integrity of the anterior visual pathway in multiple sclerosis. Mult Scler Relat Disord. 2014 Jan;3(1):117–122. PMID: 25877982. doi: 10.1016/j.msard.2013.06.014.

250. Sasaki S, Nakamura K, Ukawa S, Okada E, Amagasa S, Inoue S, et al. Association of accelerometer-measured physical activity with kidney function in a Japanese population: the DOSANCO health study. BMC Nephrol. 2022 Jan;23(1):7. PMID: 34979979. doi: 10.1186/s12882-021-02635-0.

251. Savikangas T, Suominen TH, Alén M, Rantalainen T, Sipilä S. Changes in femoral neck bone mineral density and structural strength during a 12-month multicomponent exercise intervention among older adults – does accelerometer-measured physical activity matter? Bone. 2024 Jan;178:116951. PMID: 37913888. doi: 10.1016/j.bone.2023.116951.

252. Schumacher BT, Bellettiere J, LaMonte MJ, Evenson KR, Di C, Lee IM, et al. Accelerometer-measured daily steps, physical function, and subsequent fall risk in older women: the objective physical activity and cardiovascular disease in older women study. J Aging Phys Act. 2021 Oct;30(4):635–645. PMID: 34627127. doi: 10.1123/japa.2021-0159.

253. Schwendinger F, Wagner J, Infanger D, Schmidt-Trucksäss A, Knaier R. Methodological aspects for accelerometer-based assessment of physical activity in heart failure and health. BMC Med Res Methodol. 2021 Nov;21(1):251. PMID: 34775952. doi: 10.1186/s12874-021-01350-6.

254. Scott D, Johansson J, Gandham A, Ebeling PR, Nordstrom P, Nordstrom A. Associations of accelerometer-determined physical activity and sedentary behavior with sarcopenia and incident falls over 12 months in community-dwelling Swedish older adults. J Sport Health Sci. 2021 Sep;10(5):577–584. PMID: 34088651. doi: 10.1016/j.jshs.2020.01.006.

255. Seguin-Fowler RA, LaCroix AZ, LaMonte MJ, Liu J, Maddock JE, Rethorst CD, et al. Association of neighborhood walk score with accelerometer-measured physical activity varies by neighborhood socioeconomic status in older women. Prev Med Rep. 2022 Jul;29:101931. PMID: 36161128. doi: 10.1016/j.pmedr.2022.101931.

256. Shadyab AH, LaMonte MJ, Kooperberg C, Reiner AP, Carty CL, Manini TM, et al. Association of accelerometer-measured physical activity with leukocyte telomere length among older women. J Gerontol A Biol Sci Med Sci. 2017 Oct;72(11):1532–1537. PMID: 28329327. doi: 10.1093/gerona/glx037.

257. Sharma B, Obeid J, DeMatteo C, Noseworthy MD, Timmons BW. New insights into accelerometer-measured habitual physical activity and sedentary time during early recovery in pediatric concussion. Pediatric Exerc Sci. 2023 Aug;1–8. PMID: 37591503. doi: 10.1123/pes.2023-0016.

258. Shin SW, Kim H, Kang YH, Kim J. Accelerometer-derived physical activity and sedentary behavior patterns among Korean adults. Phys Act Nutr. 2023 Jun 30;27(2):025–033. PMID: 37583069. doi: 10.20463/pan.2023.0015.

259. Si K, Cao Z, Liu Q, Yang Y, Dai Q, Yao Y, et al. Accelerometer-measured physical activity, sedentary behavior, and risk of incident pelvic organ prolapse: a prospective cohort study in the UK Biobank. Int J Behav Nutr Phys Act. 2024 Feb;21(1):12. PMID: 38308373. doi: 10.1186/s12966-024-01559-w.

260. Silva RDM, Cabral LLP, Browne RAV, Lemos TMAM, Alves CPDL, Crochemore-Silva I, et al. Joint associations of accelerometer-measured physical activity and sedentary time with cardiometabolic risk in older adults: a cross-sectional study. Exp Gerontol. 2022 Aug;165:111839. PMID: 35609715. doi: 10.1016/j.exger.2022.111839.

261. Sit CHP, Huang WY, Yu JJ, McKenzie TL. Accelerometer-assessed physical activity and sedentary time at school for children with disabilities: seasonal variation. Int J Environ Res Public Health. 2019 Aug;16(17):3163. PMID: 31480233. doi: 10.3390/ijerph16173163.

262. Sjöros T, Vähä-Ypyä H, Laine S, Garthwaite T, Lahesmaa M, Laurila SM, et al. Both sedentary time and physical activity are associated with cardiometabolic health in overweight adults in a 1 month accelerometer measurement. Sci Rep. 2020 Nov;10(1):20578. PMID: 33239818. doi: 10.1038/s41598-020-77637-3.

263. Slootmaker SM, Chinapaw MJM, Seidell JC, Van Mechelen W, Schuit AJ. Accelerometers and Internet for physical activity promotion in youth? feasibility and effectiveness of a minimal intervention [ISRCTN93896459]. Prev Med. 2010 Jul;51(1):31–36. PMID: 20380847. doi: 10.1016/j.ypmed.2010.03.015.

264. Smirnova E, Leroux A, Cao Q, Tabacu L, Zipunnikov V, Crainiceanu C, Urbanek JK. The predictive performance of objective measures of physical activity derived from accelerometry data for 5-year all-cause mortality in older adults: national health and nutritional examination survey 2003–2006. J Gerontol A Biol Sci Med Sci. 2020 Sep;75(9):1779–1785. PMID: 31504213. doi: 10.1093/gerona/glz193.

265. Smith DM, DeCaro JA, Murphy SL, Parmelee PA. Momentary reports of fatigue predict physical activity level: wrist, waist, and combined accelerometry. J Aging Health. 2020 Oct;32(9):921–925. PMID: 31319748. doi: 10.1177/0898264319863609.

266. Smith HA, Storti KL, Arena VC, Kriska AM, Gabriel KKP, Sutton-Tyrrell K, et al. Associations between accelerometer-derived physical activity and regional adiposity in young men and women: accelerometer and regional adiposity. Obesity. 2013 Jun;21(6):1299–1305. PMID: 23408709. doi: 10.1002/oby.20308.

267. Smith ID, Ross LM, Gabaldon JR, Holdgate N, Pieper CF, Ning TC, et al. The relation of accelerometer-measured physical activity and serum uric acid using the national health and nutrition survey (NHANES) 2003–2004. Front Sports Act Living. 2022 Jan;3:775398. PMID: 35098119. doi: 10.3389/fspor.2021.775398.

268. Smith KE, O’Connor SM, Mason TB, Wang S, Dzubur E, Crosby RD, et al. Associations between objective physical activity and emotional eating among adiposity‐discordant siblings using ecological momentary assessment and accelerometers. Pediatr Obes. 2021 Mar;16(3):e12720. PMID: 32881329. doi: 10.1111/ijpo.12720.

269. Spartano NL, Davis-Plourde KL, Himali JJ, Andersson C, Pase MP, Maillard P, et al. Association of accelerometer-measured light-intensity physical activity with brain volume: the Framingham heart study. JAMA Netw Open. 2019 Apr;2(4):e192745. PMID: 31002329. doi: 10.1001/jamanetworkopen.2019.2745.

270. Spartano NL, Demissie S, Himali JJ, Dukes KA, Murabito JM, Vasan RS, et al. Accelerometer‐determined physical activity and cognitive function in middle‐aged and older adults from two generations of the Framingham heart study. Alzheimers Dement. 2019 Oct;5(1):618–626. PMID: 31660424. doi: 10.1016/j.trci.2019.08.007.

271. Spartano NL, Heffernan KS, Dumas AK, Gump BB. Accelerometer-determined physical activity and the cardiovascular response to mental stress in children. J Sci Med Sport. 2017 Jan;20(1):60–65. PMID: 27283342. doi: 10.1016/j.jsams.2016.05.008.

272. Spartano NL, Wang R, Yang Q, Chernofsky A, Murabito JM, Vasan RS, et al. Association of accelerometer-measured physical activity and sedentary time with epigenetic markers of aging. Med Sci Sports Exerc. 2023 Feb;55(2):264–272. PMID: 36107108. doi: 10.1249/MSS.0000000000003041.

273. Stamatakis E, Ahmadi MN, Friedenreich CM, Blodgett JM, Koster A, Holtermann A, et al. Vigorous intermittent lifestyle physical activity and cancer incidence among nonexercising adults: the UK Biobank accelerometry study. JAMA Oncol. 2023 Sep;9(9):1255–1259. PMID: 37498576. doi: 10.1001/jamaoncol.2023.1830.

274. Stamatakis E, Ahmadi MN, Gill JMR, Thøgersen-Ntoumani C, Gibala MJ, Doherty A, et al. Association of wearable device-measured vigorous intermittent lifestyle physical activity with mortality. Nat Med. 2022 Dec;28(12):2521–2529. PMID: 36482104. doi: 10.1038/s41591-022-02100-x.

275. Stevens ML, Crowley P, Rasmussen CL, Hallman DM, Mortensen OS, Nygård CH, et al. Accelerometer-measured physical activity at work and need for recovery: a compositional analysis of cross-sectional data. Ann Work Expo Health. 2020 Feb;64(2):138–151. PMID: 31879769. doi: 10.1093/annweh/wxz095.

276. Straight CR, Ward-Ritacco CL, Evans EM. Association between accelerometer-measured physical activity and muscle capacity in middle-aged postmenopausal women. Menopause. 2015 Nov;22(11):1204–1211. PMID: 25783471. doi: 10.1097/GME.0000000000000447.

277. Strain T, Wijndaele K, Dempsey PC, Sharp SJ, Pearce M, Jeon J, et al. Wearable-device-measured physical activity and future health risk. Nat Med. 2020 Sep;26(9):1385–1391. PMID: 32807930. doi: 10.1038/s41591-020-1012-3.

278. Stubbs B, Chen LJ, Chung MS, Ku PW. Physical activity ameliorates the association between sedentary behavior and cardiometabolic risk among inpatients with schizophrenia: a comparison versus controls using accelerometry. Compr Psychiatry. 2017 Apr;74:144–150. PMID: 28167327. doi: 10.1016/j.comppsych.2017.01.010.

279. Sufrinko AM, Howie EK, Elbin RJ, Collins MW, Kontos AP. A preliminary investigation of accelerometer-derived sleep and physical activity following sport-related concussion. J Head Trauma Rehabil. 2018 Sep;33(5):E64–E74. PMID: 29601343. doi: 10.1097/HTR.0000000000000387.

280. Sutin AR, Luchetti M, Stephan Y, Terracciano A. Meaning in life and accelerometer-measured physical activity: association based on 67,038 UK Biobank participants. Ment Health Phys Act. 2021 Oct;21:100412. PMID: 35340340. doi: 10.1016/j.mhpa.2021.100412.

281. Sutin AR, Stephan Y, Kekäläinen T, Luchetti M, Terracciano A. Purpose in life and accelerometer-measured physical activity among older adults. Psychol Health. 2023 Apr;1–15. PMID:37073429. doi: 10.1080/08870446.2023.2200414.

282. Sweegers MG, Buffart LM, Huijsmans RJ, Konings IR, Van Zweeden AA, Brug J, et al. From accelerometer output to physical activity intensities in breast cancer patients. J Sci Med Sport. 2020 Feb;23(2):176–181. PMID: 31537492. doi: 10.1016/j.jsams.2019.09.001.

283. Takae R, Hatamoto Y, Yasukata J, Kose Y, Komiyama T, Ikenaga M, et al. Association of lower-extremity muscle performance and physical activity level and intensity in middle-aged and older adults: a doubly labeled water and accelerometer study. J Nutr Health Aging. 2020 Sep;24(9):1023–1030. PMID: 33155632. doi: 10.1007/s12603-020-1514-1.

284. Tanaka C, Shikano A, Imai N, Chong KH, Howard SJ, Tanabe K, et al. Accelerometer-measured physical activity and sedentary time among children in Japan before and during COVID-19: a cross-sectional and longitudinal analysis. Int J Environ Res Public Health. 2023 Jan;20(2):1130. PMID: 36673886. doi: 10.3390/ijerph20021130.

285. Tanaka C, Tanaka S. Daily phsysical activity in Japanese preschool children evaluated by triaxial accelerometry: the relationship between period of engagement in moderate-to-vigorous physical activity and daily step counts. J Physiol Anthropol. 2009 Nov;28(6):283–288. PMID: 20009376. doi: 10.2114/jpa2.28.283.

286. Tanaka C, Tanaka S, Kawahara J, Midorikawa T. Triaxial accelerometry for assessment of physical activity in young children. Obes. 2007 May;15(5):1233–1241. PMID: 17495200. doi: 10.1038/oby.2007.145.

287. Tao K, Liu W, Xiong S, Ken L, Zeng N, Peng Q, et al. Associations between self-determined motivation, accelerometer-determined physical activity, and quality of Life in Chinese college students. Int J Environ Res Public Health. 2019 Aug;16(16):2941. PMID: 31426280. doi: 10.3390/ijerph16162941.

288. Tarp J, Bugge A, Andersen LB, Sardinha LB, Ekelund U, Brage S, et al. Does adiposity mediate the relationship between physical activity and biological risk factors in youth? – a cross-sectional study from the international children’s accelerometry database (ICAD). Int J Obes. 2017 Oct;10.1038/ijo.2017.241. PMID:29087387.

289. Tarp J, Hansen BH, Fagerland MW, Steene-Johannessen J, Anderssen SA, Ekelund U. Accelerometer-measured physical activity and sedentary time in a cohort of US adults followed for up to 13 years: the influence of removing early follow-up on associations with mortality. Int J Behav Nutr Phys Act. 2020 Mar;17(1):39. PMID: 32169059. doi: 10.1186/s12966-020-00945-4.

290. Tasheva P, Kraege V, Vollenweider P, Roulet G, Méan M, Marques-Vidal P. Accelerometry assessed physical activity of older adults hospitalized with acute medical illness - an observational study. BMC Geriatr. 2020 Oct;20(1):382. PMID: 33008378. doi: 10.1186/s12877-020-01763-w.

291. Taylor RW, Williams SM, Farmer VL, Taylor BJ. Changes in physical activity over time in young children: a longitudinal study using accelerometers. PLoS One. 2013 Nov;8(11):e81567. PMID: 24282607. doi: 10.1371/journal.pone.0081567.

292. Thraen-Borowski KM, Gennuso KP, Cadmus-Bertram L. Accelerometer-derived physical activity and sedentary time by cancer type in the United States. PLoS One. 2017 Aug;12(8):e0182554. PMID: 28806753. doi: 10.1371/journal.pone.0182554.

293. Thralls KJ, Godbole S, Manini TM, Johnson E, Natarajan L, Kerr J. A comparison of accelerometry analysis methods for physical activity in older adult women and associations with health outcomes over time. J Sports Sci. 2019 Oct;37(20):2309–2317. PMID: 31195893. doi: 10.1080/02640414.2019.1631080.

294. Toivo K, Vähä‐Ypyä H, Kannus P, Tokola K, Alanko L, Heinonen OJ, et al. Physical activity measured by accelerometry among adolescents participating in sports clubs and non‐participating peers. Eur J Sport Sci. 2023 Jul;23(7):1426–1434. PMID: 35861140. doi: 10.1080/17461391.2022.2103740.

295. Toomey CM, Whittaker JL, Doyle-Baker PK, Emery CA. Does a history of youth sport-related knee injury still impact accelerometer-measured levels of physical activity after 3–12 years? Phys Ther Sport. 2022 May;55:90–97. PMID: 35290947. doi: 10.1016/j.ptsp.2022.03.003.

296. Trinh L, Motl RW, Roberts SA, Gibbons T, McAuley E. Estimation of physical activity intensity cut‐points using accelerometry in breast cancer survivors and age‐matched controls. Eur J Cancer Care. 2019 Sep;28(5). PMID: 31106924. doi: 10.1111/ecc.13090.

297. Tsai L, Boyle E, Buhl SF, Kock G, Brønd JC, Visser M, et al. Associations between appetite, physical activity and sedentary behaviour from hip‐ and wrist‐worn accelerometers in community‐dwelling older adults. Geriatrics Gerontol Int. 2023 Jun;23(6):411–417. PMID: 37098733. doi: 10.1111/ggi.14588.

298. Tsunoda K, Kitano N, Kai Y, Jindo T, Uchida K, Arao T. Dose–response relationships of accelerometer‐measured sedentary behaviour and physical activity with non‐alcoholic fatty liver disease. Aliment Pharmacol Ther. 2021 Nov;54(10):1330–1339. PMID: 34633105. doi: 10.1111/apt.16631.

299. Vaara JP, Kyröläinen H, Vasankari T, Kainulainen H, Raitanen J, Kujala UM. Accelerometer-based sedentary time, physical activity, and serum metabolome in young men. Metabolites. 2022 Jul;12(8):700. PMID: 36005572. doi: 10.3390/metabo12080700.

300. Van Bakergem M, Sommer EC, Heerman WJ, Hipp JA, Barkin SL. Objective reports versus subjective perceptions of crime and their relationships to accelerometer-measured physical activity in Hispanic caretaker-child dyads. Prev Med. 2017 Feb;95:S68–S74. PMID: 27939263. doi: 10.1016/j.ypmed.2016.12.001.

301. Van Ballegooijen AJ, Van Der Ploeg HP, Visser M. Daily sedentary time and physical activity as assessed by accelerometry and their correlates in older adults. Eur Rev Aging Phys Act. 2019 Feb;16(1):3. PMID: 30820261. doi: 10.1186/s11556-019-0210-9.

302. Van Der Zee-Neuen A, Wirth W, Hösl K, Osterbrink J, Eckstein F. The association of physical activity and depression in patients with, or at risk of, osteoarthritis is captured equally well by patient reported outcomes (PROs) and accelerometer measurements - analyses of data from the osteoarthritis initiative. Semin Arthritis Rheum. 2019 Dec;49(3):325–330. PMID: 31248586. doi: 10.1016/j.semarthrit.2019.05.009.

303. Van Dyck D, Barnett A, Cerin E, Conway TL, Esteban-Cornejo I, Hinckson E, et al. Associations of accelerometer measured school- and non-school based physical activity and sedentary time with body mass index: IPEN adolescent study. Int J Behav Nutr Phys Act. 2022 Jul;19(1):85. PMID: 35836235. doi: 10.1186/s12966-022-01324-x.

304. Van Dyck D, Cerin E, De Bourdeaudhuij I, Salvo D, Christiansen LB, Macfarlane D, et al. Moderating effects of age, gender and education on the associations of perceived neighborhood environment attributes with accelerometer-based physical activity: the IPEN adult study. Health Place. 2015 Nov;36:65–73. PMID: 26454247. doi: 10.1016/j.healthplace.2015.09.007.

305. Van Eijk RPA, Bakers JNE, Bunte TM, De Fockert AJ, Eijkemans MJC, Van Den Berg LH. Accelerometry for remote monitoring of physical activity in amyotrophic lateral sclerosis: a longitudinal cohort study. J Neurol. 2019 Oct;266(10):2387–2395. PMID: 31187191. doi: 10.1007/s00415-019-09427-5.

306. Van Genderen S, Boonen A, Van Der Heijde D, Heuft L, Luime J, Spoorenberg A, et al. Accelerometer quantification of physical activity and activity patterns in patients with ankylosing spondylitis and population controls. J Rheumatol. 2015 Dec;42(12):2369–2375. PMID: 26523021. doi: 10.3899/jrheum.150015.

307. Van Landingham SW, Willis JR, Vitale S, Ramulu PY. Visual field loss and accelerometer-measured physical activity in the United States. Ophthalmology. 2012 Dec;119(12):2486–2492. PMID: 22892152. doi: 10.1016/j.ophtha.2012.06.034.

308. Van Langenberg DR, Papandony MC, Gibson PR. Sleep and physical activity measured by accelerometry in Crohn’s disease. Aliment Pharmacol Ther. 2015 May;41(10):991–1004. PMID: 25783784. doi: 10.1111/apt.13160.

309. Vandercappellen EJ, Koster A, Savelberg HHCM, Eussen SJPM, Dagnelie PC, Schram MT, et al. Accelerometer-derived physical activity and sedentary time and cardiac biomarkers: the Maastricht study. Front Cardiovasc Med. 2023 Apr 28;10:1081713. PMID: 37187790. doi: 10.3389/fcvm.2023.1081713.

310. Vásquez E, Strizich G, Isasi CR, Echeverria SE, Sotres-Alvarez D, Evenson KR, et al. Is there a relationship between accelerometer-assessed physical activity and sedentary behavior and cognitive function in US Hispanic/Latino adults? the Hispanic community health study/study of Latinos (HCHS/SOL). Prev Med. 2017 Oct;103:43–48. PMID: 28765082. doi: 10.1016/j.ypmed.2017.07.024.

311. Vásquez PM, Tarraf W, Chai A, Doza A, Sotres-Alvarez D, Diaz KM, et al. Accelerometer-measured latent physical activity profiles and neurocognition among middle-aged and older Hispanic/Latino adults in the Hispanic community health study/study of Latinos (HCHS/SOL). J Gerontol B Psychol Sci Soc Sci. 2022 Dec;77(12):e263–e278. PMID: 36219450. doi: 10.1093/geronb/gbac161.

312. Vásquez PM, Durazo-Arvizu RA, Marquez DX, Argos M, Lamar M, Odoms-Young A, et al. Association of accelerometer-measured physical activity and cardiovascular health in the Hispanic community health study/study of Latinos (HCHS/SOL). Hisp Health Care Int. 2022 Mar;20(1):15–24. PMID: 33685281. doi: 10.1177/1540415320985581.

313. Verbestel V, De Henauw S, Bammann K, Barba G, Hadjigeorgiou C, Eiben G, et al. Are context-specific measures of parental-reported physical activity and sedentary behaviour associated with accelerometer data in 2–9-year-old European children? Public Health Nutr. 2015 Apr;18(5):860–868. PMID: 24887315. doi: 10.1017/S136898001400086X.

314. Vorwerg Y, Petroff D, Kiess W, Blüher S. Physical activity in 3–6 year old children measured by SenseWear Pro®: direct accelerometry in the course of the week and relation to weight status, media consumption, and socioeconomic factors. PLoS One. 2013 Apr;8(4):e60619. PMID: 23573273. doi: 10.1371/journal.pone.0060619.

315. Watts EL, Saint-Maurice PF, Doherty A, Fensom GK, Freeman JR, Gorzelitz JS, et al. Association of accelerometer-measured physical activity level with risks of hospitalization for 25 common health conditions in UK adults. JAMA Netw Open. 2023 Feb;6(2):e2256186. PMID: 36795414. doi: 10.1001/jamanetworkopen.2022.56186.

316. Weaver RG, Crimarco A, Brusseau TA, Webster CA, Burns RD, Hannon JC. Accelerometry‐derived physical activity of first through third grade children during the segmented school day. J Sch Health. 2016 Oct;86(10):726–733. PMID: 27619763. doi: 10.1111/josh.12426.

317. Wendt A, Da Silva ICM, Gonçalves H, Menezes A, Barros F, Wehrmeister FC. Short-term effect of physical activity on sleep health: a population-based study using accelerometry. J Sport Health Sci. 2022 Sep;11(5):630–638. PMID: 32422346. doi: 10.1016/j.jshs.2020.04.007.

318. Werneck AO, Jago R, Kriemler S, Andersen LB, Wedderkopp N, Northstone K, et al. Association of change in the school travel mode with changes in different physical activity intensities and sedentary time: a international children’s accelerometry database study. Prev Med. 2021 Dec;153:106862. PMID: 34710443. doi: 10.1016/j.ypmed.2021.106862.

319. Whitaker KM, Pettee Gabriel K, Buman MP, Pereira MA, Jacobs DR, Reis JP, et al. Associations of accelerometer‐measured sedentary time and physical activity with prospectively assessed cardiometabolic risk factors: the CARDIA study. J Am Heart Assoc. 2019 Jan;8(1):e010212. PMID: 30616480. doi: 10.1161/JAHA.118.010212.

320. Whitaker KM, Pettee Gabriel K, Laddu D, White DK, Sidney S, Sternfeld B, et al. Bidirectional associations of accelerometer measured sedentary behavior and physical activity with knee pain, stiffness, and physical function: the CARDIA study. Prev Med Rep. 2021 Mar;22:101348. PMID: 33816086. doi: 10.1016/j.pmedr.2021.101348.

321. Whitaker KM, Xiao Q, Pettee Gabriel K, Gordon Larsen P, Jacobs DR, Sidney S, et al. Perceived and objective characteristics of the neighborhood environment are associated with accelerometer-measured sedentary time and physical activity, the CARDIA Study. Prev Med. 2019 Jun;123:242–249. PMID: 30940573. doi: 10.1016/j.ypmed.2019.03.039.

322. Whitaker KM, Zhang D, Pettee Gabriel K, Ahrens M, Sternfeld B, Sidney S, et al. Longitudinal associations of midlife accelerometer determined sedentary behavior and physical activity with cognitive function: the CARDIA study. J Am Heart Assoc. 2021 Feb;10(3):e018350. PMID: 33470140. doi: 10.1161/JAHA.120.018350.

323. White T, Westgate K, Wareham NJ, Brage S. Estimation of physical activity energy expenditure during free-living from wrist accelerometry in UK adults. PLoS One. 2016 Dec 9;11(12):e0167472. PMID: 27936024. doi: 10.1371/journal.pone.0167472.

324. Willis JR, Jefferys JL, Vitale S, Ramulu PY. Visual impairment, uncorrected refractive error, and accelerometer-defined physical activity in the United States. Arch Ophthalmol. 2012 Mar;130(3):329-35. PMID: 22411662. doi: 10.1001/archopthalmol.2011.1773.

325. Wu J, Olson JL, Brunke-Reese D, Lagoa CM, Conroy DE. Wearable device adherence among insufficiently-active young adults is independent of identity and motivation for physical activity. J Behav Med. 2024 Apr;47(2):197–206. PMID: 37642938. doi: 10.1007/s10865-023-00444-4.

326. Wu Y, Goodrich JM, Dolinoy DC, Sánchez BN, Ruiz-Narváez EA, Banker M, et al. Accelerometer-measured physical activity, reproductive hormones, and DNA methylation. Med Sci Sports Exerc. 2020 Mar;52(3):598–607. PMID: 31652236. doi: 10.1249/MSS.0000000000002175.

327. Wu Z, Wang Z, Hu B, Zhang X, Zhang F, Wang H, et al. Relationships of accelerometer-based measured objective physical activity and sedentary behaviour with cognitive function: a comparative cross-sectional study of China’s elderly population. BMC Geriatr. 2020 Apr;20(1):149. PMID: 32321436. doi: 10.1186/s12877-020-01521-y.

328. Yamaga Y, Svensson T, Chung U, Svensson AK. Association between metabolic syndrome status and daily physical activity measured by a wearable device in Japanese office workers. Int J Environ Res Public Health. 2023 Feb;20(5):4315. PMID: 36901325. doi: 10.3390/ijerph20054315.

329. Yang CH, Wang S, Wang WL, Belcher BR, Dunton GF. Day-level associations of physical activity and sedentary time in mother–child dyads across three years: a multi-wave longitudinal study using accelerometers. J Behav Med. 2022 Oct;45(5):702–715. PMID: 35753007. doi: 10.1007/s10865-022-00335-0.

330. Yang W, Li MH, Yu JJ, Wong SH, Sum RK-W, Sit CH. The associations between accelerometer-measured physical activity levels and mental health in children and adolescents with intellectual disabilities during the COVID-19 pandemic. J Autism Dev Disord. 2023 Dec;53(12):4809–4821. PMID: 36181649. doi: 10.1007/s10803-022-05777-8.

331. Yen HY, Liao Y, Huang HY. Smart wearable device users’ behavior is essential for physical activity improvement. Int J Behav Med. 2022 Jun;29(3):278–285. PMID: 34363130. doi: 10.1007/s12529-021-10013-1.

332. Yerrakalva D, Hajna S, Wijndaele K, Dempsey PC, Westgate K, Wareham N, et al. Bidirectional associations of accelerometer-assessed physical activity and sedentary time with physical function among older English adults: the EPIC-Norfolk cohort study. Eur J Ageing. 2022 Oct;19(4):1507–1517. PMID: 36506675. doi: 10.1007/s10433-022-00733-y.

333. Yi L, Mason TB, Yang CH, Chu D, Dunton GF. Longitudinal associations between neighborhood park and open space access and children’s accelerometer-assessed Measured physical activity: the evidence from the MATCH study. J Phy Act Health. 2021 Jul;18(9):1058–1066. PMID: 34198261. doi: 10.1123/jpah.2021-0177.

334. Yoshioka M, Ayabe M, Yahiro T, Higuchi H, Higaki Y, St-Amand J, et al. Long-period accelerometer monitoring shows the role of physical activity in overweight and obesity. Int J Obes. 2005 May;29(5):502–508. PMID: 15672105. doi: 10.1038/sj.ijo.0802891.

335. You Y, Chen Y, Wang X, Wei M, Zhang Q, Cao Q. Accelerometer-measured physical activity patterns are associated with phenotypic age: Isotemporal substitution effects. Heliyon. 2023 Aug;9(9):e19158. PMID: 37810111. doi: 10.1016/j.heliyon.2023.e19158.

336. Yu X, Hao L, Crainiceanu C, Leroux A. Occupational determinants of physical activity at work: evidence from wearable accelerometer in 2005–2006 NHANES. SSM Popul Health. 2021 Dec;17:100989. PMID: 34977325. doi: 10.1016/j.ssmph.2021.100989.

337. Zhang X, Yang Z, Du L, Xiong C, Wang Z, Pan Y, et al. Associations of accelerometer-measured physical activity, sedentary behavior with self-reported insomnia in older women: does pattern matter? Sleep Med. 2023 Apr;104:58–63. PMID: 36906996. doi: 10.1016/j.sleep.2023.02.015.

338. Zhong Q, Zhou R, Huang YN, Chen HW, Liu HM, Huang Z, et al. The independent and joint association of accelerometer-measured physical activity and sedentary time with dementia: a cohort study in the UK Biobank. Int J Behav Nutr Phys Act. 2023 May;20(1):59. PMID: 37198574. doi: 10.1186/s12966-023-01464-8.

339. Zhou W, Veliz PT, Smith EML, Chen W, Reddy RM, Larson JL. Comparison of pre-diagnosis ohysical activity and its correlates between lung and other cancer patients: accelerometer data from the UK Biobank prospective cohort. Int J Environ Res Public Health. 2023 Jan;20(2):1001. PMID: 36673757. doi: 10.3390/ijerph20021001.

340. Zijlstra WMH, Ploegstra MJ, Vissia-Kazemier T, Roofthooft MTR, Sarvaas GDM, Bartelds B, et al. Physical activity in pediatric pulmonary arterial hypertension measured by accelerometry. A candidate clinical endpoint. Am J Respir Crit Care Med. 2017 Jul;196(2):220–227. PMID: 28178426. doi: 10.1164/rccm.201608-1576OC.

341. Zisko N, Nauman J, Sandbakk SB, Aspvik NP, Salvesen Ø, Carlsen T, et al. Absolute and relative accelerometer thresholds for determining the association between physical activity and metabolic syndrome in the older adults: the Generation-100 study. BMC Geriatr. 2017 May;17(1):109. PMID: 28511695. doi: 10.1186/s12877-017-0497-1.

342. Zlatar ZZ, Hays CC, Mestre Z, Campbell LM, Meloy MJ, Bangen KJ, et al. Dose-dependent association of accelerometer-measured physical activity and sedentary time with brain perfusion in aging. Exp Gerontol. 2019 Oct;125:110679. PMID: 31382010. doi: 10.1016/j.exger.2019.110679.

**Articles categorized in the prediction study (n=32):**

1. Anastasopoulou P, Tansella M, Stumpp J, Shammas L, Hey S. Classification of human physical activity and energy expenditure estimation by accelerometry and barometry. 2012 Annual International Conference of the IEEE Engineering in Medicine and Biology Society. 2012 Aug 28- Sep 01; San Diego, CA. New York: IEEE; 2012. PMID: 23367406. doi: 10.1109/EMBC.2012.6347471.

2. Assah FK, Ekelund U, Brage S, Corder K, Wright A, Mbanya JC, et al. Predicting physical activity energy expenditure using accelerometry in adults from sub‐Sahara Africa. Obesity. 2009 Aug;17(8):1588–1595. PMID: 19247268. doi: 10.1038/oby.2009.39.

3. Bonomi AG, Plasqui G, Goris AH, Westerterp KR. Improving assessment of daily energy expenditure by identifying types of physical activity with a single accelerometer. J Appl Physiol. 2009 Sep;107(3):655–661. PMID: 19556460. doi: 10.1152/japplphysiol.00150.2009.

4. Chen M, Landré B, Marques-Vidal P, Van Hees VT, Van Gennip ACE, Bloomberg M, et al. Identification of physical activity and sedentary behaviour dimensions that predict mortality risk in older adults: development of a machine learning model in the Whitehall II accelerometer sub-study and external validation in the CoLaus study. EClinicalMedicine. 2023 Jan;55:101773. PMID: 36568684. doi: 10.1016/j.eclinm.2022.101773.

5. Crouter SE, Flynn JI, Bassett DR. Estimating physical activity in youth using a wrist accelerometer. Med Sci Sports Exerc. 2015 May;47(5):944–951. PMID: 25207928. doi: 10.1249/MSS.0000000000000502.

6. Crouter SE, Oody JF, Bassett DR. Estimating physical activity in youth using an ankle accelerometer. J Sports Sci. 2018 Oct;36(19):2265–2271. PMID: 29517959. doi: 10.1080/02640414.2018.1449091

7. Diniz-Sousa F, Veras L, Ribeiro JC, Boppre G, Devezas V, Santos-Sousa H, et al. Accelerometry calibration in people with class II-III obesity: Energy expenditure prediction and physical activity intensity identification. Gait Posture. 2020 Feb;76:104–109. PMID: 31756665. doi: 10.1016/j.gaitpost.2019.11.008.

8. Ellis K, Kerr J, Godbole S, Lanckriet G, Wing D, Marshall S. A random forest classifier for the prediction of energy expenditure and type of physical activity from wrist and hip accelerometers. Physiol Meas. 2014 Dec;35(11):2191–2203. PMID: 25340969. doi: 10.1088/0967-3334/35/11/2191.

9. Farooq M, Sazonov E. A novel wearable device for food intake and physical activity recognition. Sensors. 2016 Jul;16(7):1067. PMID: 27409622. doi: 10.3390/s16071067.

10. Gao N, Shao W, Salim FD. Predicting personality traits from physical activity intensity. Comput. 2019 Jul;52(7):47–56. doi: 10.1109/MC.2019.2913751.

11. Garnotel M, Bastian T, Romero-Ugalde HM, Maire A, Dugas J, Zahariev A, et al. Prior automatic posture and activity identification improves physical activity energy expenditure prediction from hip-worn triaxial accelerometry. J Appl Physiol. 2018 Mar;124(3):780–790. PMID: 29191980. doi: 10.1152/japplphysiol.00556.2017.

12. Jeran S, Steinbrecher A, Haas V, Mähler A, Boschmann M, Westerterp KR, et al. Prediction of activity-related energy expenditure under free-living conditions using accelerometer-derived physical activity. Sci Rep. 2022 Oct 4;12(1):16578. PMID: 36195647. doi: 10.1038/s41598-022-20639-0.

13. Ladlow P, Nightingale TE, McGuigan MP, Bennett AN, Phillip R, Bilzon JLJ. Impact of anatomical placement of an accelerometer on prediction of physical activity energy expenditure in lower-limb amputees. PLoS One. 2017 Oct;12(10):e0185731. PMID: 28982199. doi: 10.1371/journal.pone.0185731.

14. Mardini MT, Bai C, Wanigatunga AA, Saldana S, Casanova R, Manini TM. Age differences in estimating physical activity by wrist accelerometry using machine learning. Sensors. 2021 May;21(10):3352. PMID: 34065906. doi: 10.3390/s21103352.

15. Matabuena M, Félix P, Hammouri ZAA, Mota J, Del Pozo Cruz B. Physical activity phenotypes and mortality in older adults: a novel distributional data analysis of accelerometry in the NHANES. Aging Clin Exp Res. 2022 Dec;34(12):3107–3114. PMID: 36183279. doi: 10.1007/s40520-022-02260-3.

16. Midorikawa T, Tanaka S, Kaneko K, Koizumi K, Ishikawa‐Takata K, Futami J, et al. Evaluation of low‐intensity physical activity by triaxial accelerometry. Obesity. 2007 Dec;15(12):3031–3038. PMID: 18198312. doi: 10.1038/oby.2007.361.

17. Miller NE, Strath SJ, Swartz AM, Cashin SE. Estimating absolute and relative physical activity intensity across Age via accelerometry in adults. J Aging Phys Act. 2010 Apr;18(2):158–170. PMID: 20440028. doi: 10.1123/japa.18.2.158.

18. Nagayoshi S, Oshima Y, Ando T, Aoyama T, Nakae S, Usui C, et al. Validity of estimating physical activity intensity using a triaxial accelerometer in healthy adults and older adults. BMJ Open Sport Exerc Med. 2019 Oct;5(1):e000592. PMID: 31749982. doi: 10.1136/bmjsem-2019-000592.

19. Nam S, Jeon S, Ash G, Whittemore R, Vlahov D. Racial discrimination, sedentary time, and physical activity in african americans: quantitative study combining ecological momentary assessment and accelerometers. JMIR Form Res. 2021 Jun;5(6):e25687. PMID: 34096870. doi: 10.2196/25687.

20. Nawab KA, Storey BC, Staplin N, Walmsley R, Haynes R, Sutherland S, et al. Accelerometer-measured physical activity and functional behaviours among people on dialysis. Clin Kidney J. 2020 Aug;14(3):950–958. PMID: 33777379. doi: 10.1093/ckj/sfaa045.

21. Ortlieb S, Dias A, Gorzelniak L, Nowak D, Karrasch S, Peters A, et al. Exploring patterns of accelerometry-assessed physical activity in elderly people. Int J Behav Nutr Phys Act. 2014 Feb;11(1):28. PMID: 24575796. doi: 10.1186/1479-5868-11-28.

22. Park J, Ishikawa-Takata K, Tanaka S, Mekata Y, Tabata I. Effects of walking speed and step frequency on estimation of physical activity Using accelerometers. J Physiol Anthropol. 2011;30(3):119–127. PMID: 21636955. doi: 10.2114/jpa2.30.119.

23. Parkka J, Ermes M, Antila K, Van Gils M, Manttari A, Nieminen H. Estimating intensity of physical activity: a comparison of wearable accelerometer and gyro sensors and 3 Sensor locations. 2007 29th Annual International Conference of the IEEE Engineering in Medicine and Biology Society; 2007 Oct 22; Lyon, France. New York: IEEE; 2007. PMID: 18002254. doi: 10.1109/IEMBS.2007.4352588.

24. Plasqui G, Joosen AMCP, Kester AD, Goris AHC, Westerterp KR. Measuring free‐living energy expenditure and physical activity with triaxial accelerometry. Obes Res. 2005 Aug;13(8):1363–1369. PMID: 16129718. doi: 10.1038/oby.2005.165.

25. Smirnova E, Leroux A, Cao Q, Tabacu L, Zipunnikov V, Crainiceanu C, Urbanek JK. The predictive performance of objective measures of physical activity derived from accelerometry data for 5-year all-cause mortality in older adults: national health and nutritional examination survey 2003–2006. J Gerontol A Biol Sci Med Sci. 2020 Sep;75(9):1779–1785. PMID: 31504213. doi: 10.1093/gerona/glz193.

26. Tabacu L, Ledbetter M, Leroux A, Crainiceanu C, Smirnova E. Quantifying the varying predictive value of physical activity measures obtained from wearable accelerometers on all-cause mortality over short to medium time horizons in NHANES 2003–2006. Sensors. 2020 Dec;21(1):4. PMID: 33374911. doi: 10.3390/s21010004.

27. Tanaka C, Hikihara Y, Ando T, Oshima Y, Usui C, Ohgi Y, et al. Prediction of physical activity intensity with accelerometry in young children. Int J Environ Res Public Health. 2019 Mar;16(6):931. PMID: 30875871. doi: 10.3390/ijerph16060931.

28. Tanaka C, Tanaka S, Kawahara J, Midorikawa T. Triaxial accelerometry for assessment of physical activity in young children. Obes. 2007 May;15(5):1233–1241. PMID: 17495200. doi: 10.1038/oby.2007.145.

29. Tanaka S, Ishikawa-Takata K, Nakae S, Sasaki S. Prediction of the physical activity level of community-dwelling older Japanese adults with a triaxial accelerometer containing a classification algorithm for ambulatory and non-ambulatory activities. Sensors. 2023 May;23(10):4960. PMID: 37430874. doi: 10.3390/s23104960.

30. Valenti G, Camps SGJA, Verhoef SPM, Bonomi AG, Westerterp KR. Validating measures of free-living physical activity in overweight and obese subjects using an accelerometer. Int J Obes. 2014 Jul;38(7):1011–1014. PMID: 24166066. doi: 10.1038/ijo.2013.195.

31. Verma VK, Lin WY. Machine learning-based 30-day hospital readmission predictions for COPD patients using physical activity data of daily living with accelerometer-based device. Biosensors. 2022 Aug;12(8):605. PMID: 36005000. doi: 10.3390/bios12080605.

32. Wang Y, Wattelez G, Frayon S, Caillaud C, Galy O, Yacef K. ABIPA: ARIMA-based integration of accelerometer-based physical activity for adolescent weight status prediction. ACM Trans Comput Healthcare. 2023 Jan;4(1):1–19. doi: 10.1145/3561611.
